# Stereoselective Synthesis of Polysubstituted Spiropentanes

**DOI:** 10.1021/jacs.2c07370

**Published:** 2022-09-11

**Authors:** Yair Cohen, Dor Toledano, Ilan Marek

**Affiliations:** Schulich Faculty of Chemistry, Technion - Israel Institute of Technology, Technion City, Haifa, 32000, Israel

## Abstract

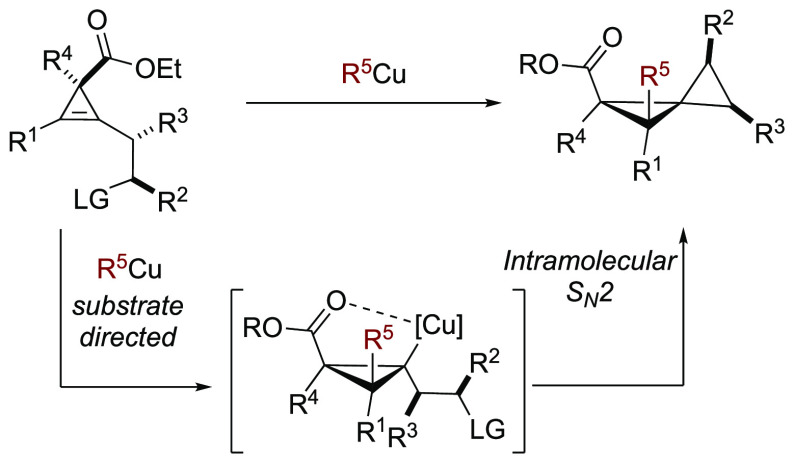

A new approach to polysubstituted spiropentanes is developed through a regio- and diastereoselective carbometalation of sp^2^-disubstituted cyclopropenes. The control of selectivity originates from a combined *syn*-facial diastereoselective carbometalation with a regio-directed addition. The regio-controlling element subsequently serves as a leaving group in an intramolecular nucleophilic substitution. This method allows the preparation of various polysubstituted spiropentanes with up to five contiguous stereocenters.

Spiropentane, the smallest spiro-connected cycloalkane, has been known since the end of the 19th century. First synthesized by Gustavson in 1896,^1^ the correct structure was proposed by Zelinsky two decades later,^2^ and spectroscopically characterized by Murray in 1944.^3^ Since then, spiropentanes have aroused the imagination of theoretical,^4^ physical,^5^ and synthetic chemists,^6^ as well as biologists as some members display interesting biological activities.^7^ The synthesis of these highly strained spirocyclic hydrocarbons have been extensively investigated,^6^ initially through 1,3-reductive dehalogenation reactions (Scheme 1a).^3a,8^ To reach a slightly higher degree of substitution, cyclopropyl sulfur ylides were added to α–β unsaturated carbonyl derivatives (Scheme 1b).^9^ Another approach is the carbene (in situ generated by metal-catalyzed decomposition of diazo compounds) addition to alkylidenecyclopropane or allene to produce spiropentanes with different substitution patterns (Scheme 1c).^10^ Using zinc carbenoid in the presence of chiral dioxaborolane ligand led to a substituted spiropentane structure with excellent enantiomeric ratios (Scheme 1d).^11^ In a real tour de force, de Meijere reported the first enantiomerically enriched unbranched [4]triangulane synthesis (Scheme 1e).^12^ Nevertheless, all reported methods mentioned above, among others,^6^ lack flexibility with a rather limited number of substitution patterns, particularly if one is interested in the formation of quaternary carbon stereocenters. In our recent study concerning the selective ring opening of spiropentane derivatives toward the formation of distant stereocenters in acyclic systems (Scheme 1f),^13^ we indeed realized the difficulty to prepare polysubstituted spiropentane with high diastereomeric and enantiomeric ratios.

**Scheme 1:**
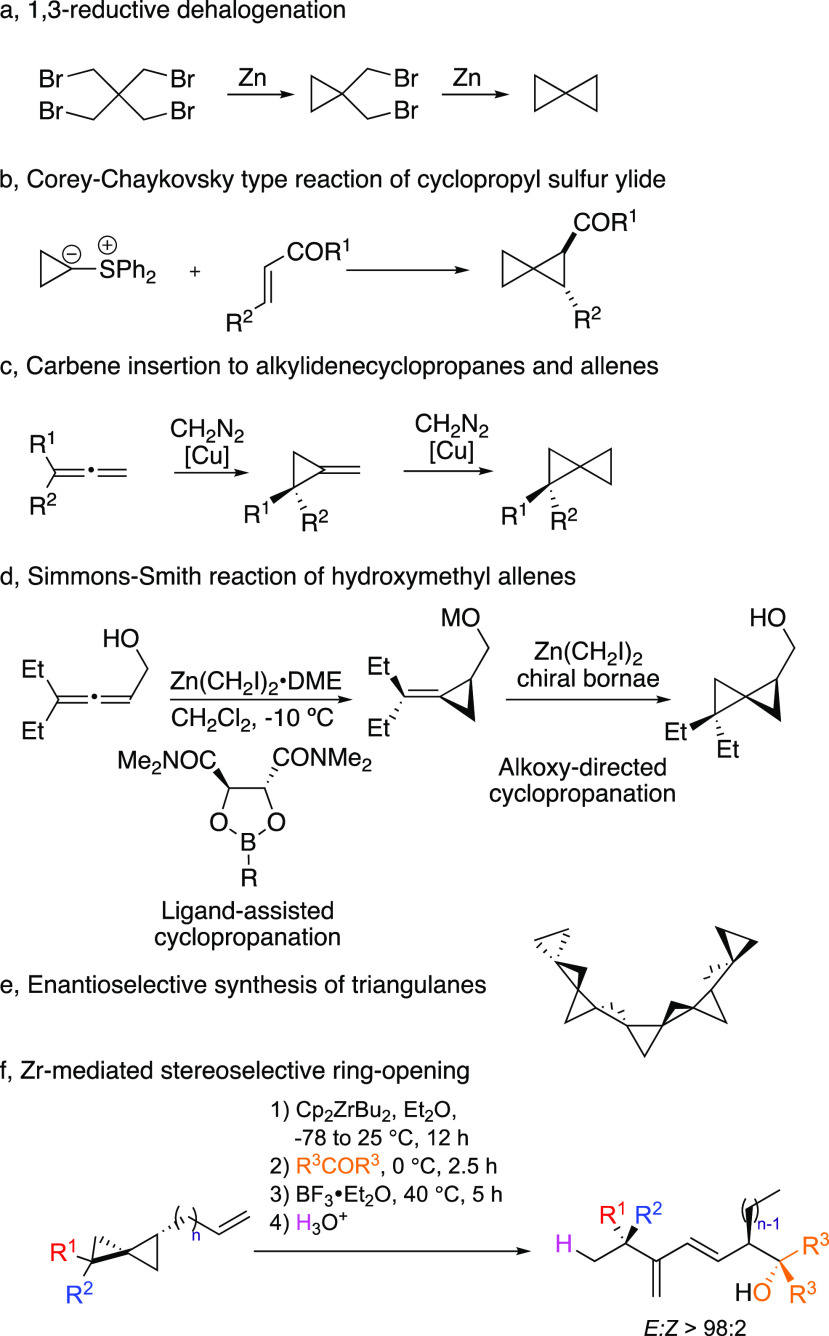
Synthetic Routes to Spiropentanes and Reactivity

Based on the limited number of synthetic approaches allowing a convergent preparation of polysubstituted spiropentanes as a single diastereomer, we decided to start a new program to handle this issue. For the past few years, we and others have developed several strategies to prepare polysubstituted cyclopropanes^14^ including the formation of *per*(hexa)-substituted cyclopropanes as a single diastereomer through regio- and diastereoselective carbometalation reactions of cyclopropenes.^15^ During this study, we have established a protocol for a regio- and diastereoselective copper-catalyzed addition of alkylmagnesium halide to electronically biased sp^2^-disubstituted cyclopropene,^15^ as well as to sp^2^-dialkylated substituted cyclopropenes where the regioselectivity is controlled by a template effect (Scheme 2a).^16^

**Scheme 2:**
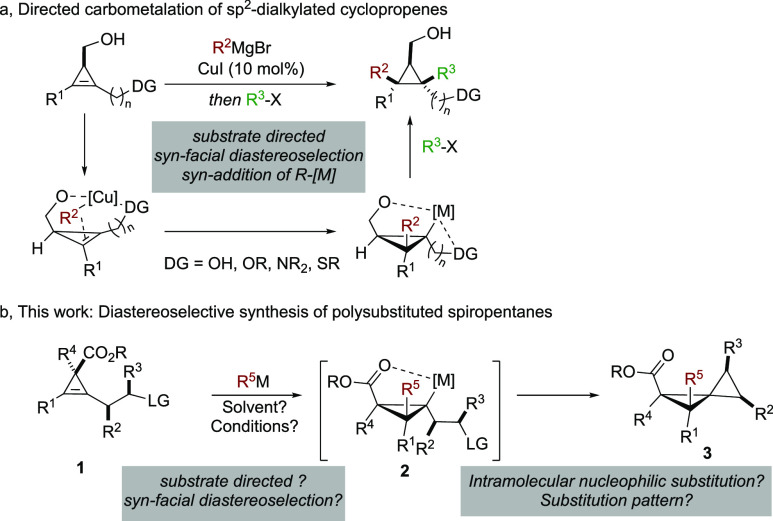
Regio- and Diastereocontrolled Carbometalation Reactions and Formation of Spiropentane

In the latter case, the organometallic nucleophile is doubly directed: (i) the alcoholate induces a *syn*-facial diastereoselectivity of the addition and (ii) a directing group tethered to one end of the double bond dictates the regioselectivity of the addition. Several directing groups (DGs) could govern the regioselectivity such as free and protected alcohols, tertiary amines, and sulfides.^16^

Based on this directing group ability to control the regioselectivity of addition to 1,2-dialkylated cyclopropenes, we envisioned the use of a functionality that not only possessed the capacity to direct the addition step but also could serve as a good leaving group.^17^ It should allow a subsequent intramolecular nucleophilic substitution reaction giving rise to the preparation of polysubstituted spiropentane derivatives (Scheme 2b). Obviously, several challenges and pitfalls need to be overcome. First and foremost, an easy and efficient preparation of all starting materials as a single diastereomer should be delineated. Then, the regioselectivity as well as the diastereoselectivity of addition should be controlled implying that the nature of the directing group/leaving group should match the *syn*-facial effect of the ester of **1** (matched pair, Scheme 3). Assuming that the carbometalation reaction will be fully regio- and diastereoselective (**1** into **2**), the subsequent step consists of an intramolecular nucleophilic substitution of a tertiary cyclopropyl metal species **2** to a secondary halide or pseudo halide that might lead to elimination reactions rather than substitution. Finally, the required substitution should proceed faster than a ring-fragmentation that is a known side-reaction of cyclopropyl metal possessing an electron withdrawing group (donor–acceptor cyclopropane,^18^Scheme 3).

**Scheme 3:**
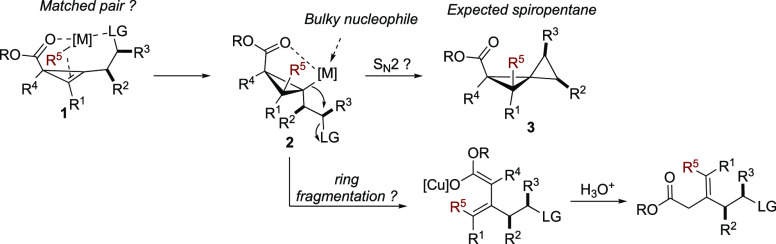
Potential Pitfalls

With these potential pitfalls in mind, we started our study by devising a synthesis of our starting materials. Cyclopropenyl esters **4** are easily obtained by a Rh-catalyzed decomposition of diazoester in the presence of an alkyne. From racemic **4**, a simple metalation at low temperature and addition of epoxides in the presence of a Lewis acid led to the equimolar amount of the two diastereomers **5a** and **5b** (see the Supporting Information).^19^ It should be noted that formally a single enantiomer as well as diastereomer could be obtained by using the enantioselective Rh-catalyzed cyclopropenation of alkynes that would provide **4** in excellent enantiomeric ratios^20^ followed by addition of enantiomerically pure epoxide. However, as the two diastereomers were easily separated by purification by column chromatography, we have initially opted to continue with racemic **4**. It is interesting to note that 6-membered ring lactone was not detected in the crude reaction mixture underlining the unfavorable cyclization process. Then, the corresponding alcohols were transformed into tosylate **1** that would serve, on one hand, as a directing template to control the regioselectivity of the carbometalation reaction and, on the other hand, as a good leaving group toward intramolecular nucleophilic substitution. The chemistry described above could be easily run on a preparatory scale (0.5 to gram scale) which makes the method attractive despite unoptimized modest yields in some cases (please note that yields for **5a** and **5b** are after separation of the two diastereomers by column chromatography and are based on a maximum 50%, Scheme 4). In some cases, the ester was also reduced into alcohols 7. The relative configuration was determined by X-ray analysis of **1a-8**, and all other configurations were assigned by analogy.

**Scheme 4:**
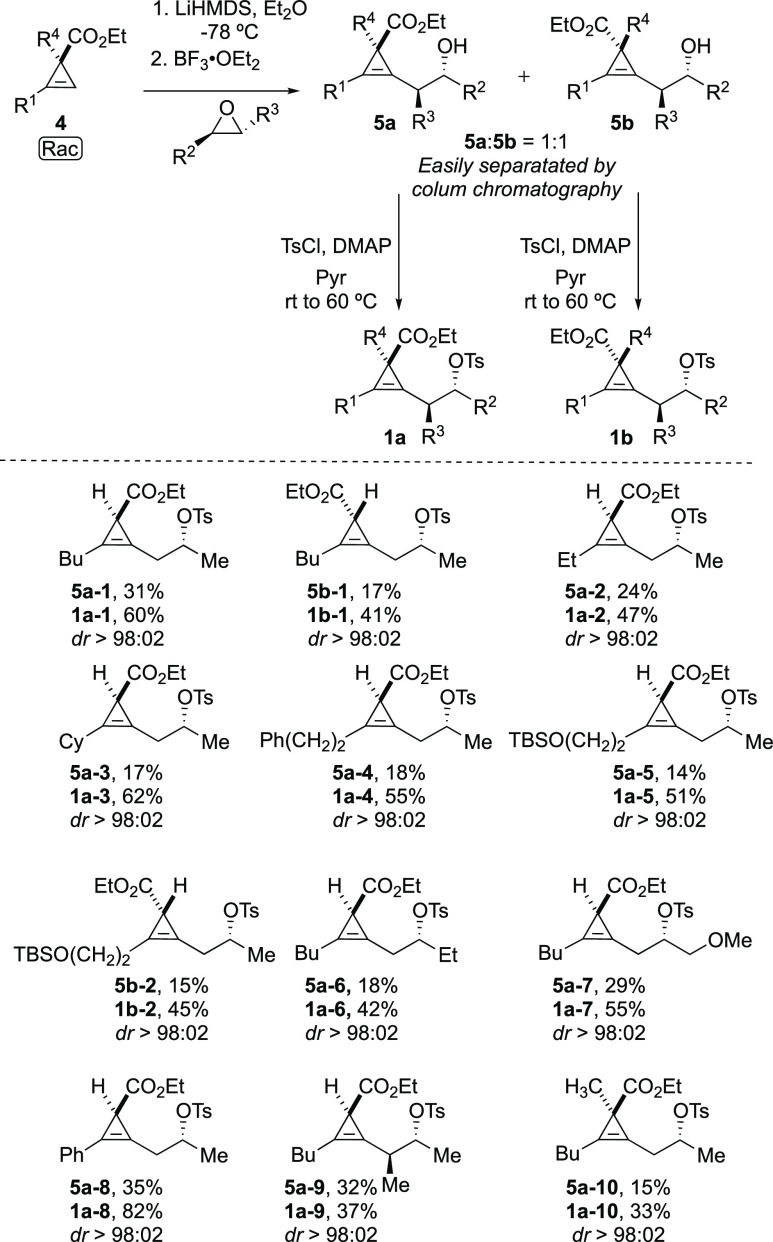
Preparation of Starting Materials

Having in hand an easy access to a large variety of starting materials **1a** and **1b**, we then turned our attention to the tandem regio- and diastereoselective carbometalation reaction followed by intramolecular nucleophilic substitution as a new route to polysubstituted spiropentanes **3**. When our model substrate **1a-1** was treated with an organocopper reagent (easily prepared by addition of 1 equiv of alkyllithium to 1 equiv of CuI)^21^ in a mixture of Et_2_O/toluene as solvent, the carbometalation reaction proceeded smoothly at low temperature. The resulting cyclopropyl copper species could not be isolated as it undergoes a rapid cyclization to provide spiropentane **3a-1** as a single diastereomer in excellent yield (Scheme 5). We were happy that no ring fragmentation of the cyclopropyl copper species was observed, owing to the relatively high covalent nature of the carbon–copper bond. The nature of the alkyl group of the organocopper can be varied as various primary and even secondary alkyl lithiums could be used as precursors of the organocopper (**3a-1** to **3a-4**, Scheme 5). It should be noted that the diastereoselectivity in the latter case is lower due to potential steric hindrance in the carbometalation step. Both diastereomers at the quaternary carbon center could be easily prepared by simply permuting the nature of the alkyl groups on the cyclopropene and organocopper (compare **3a-2** and **3a-3**, Scheme 5). Similarly, various alkyl chains R^1^ of different nature are compatible, including a phenyl substituent (**3a-5** to **3a-7**, Scheme 3). The nature of the substituents on the epoxide could be varied including with a more functionalized group (**3a-8** and **3a-9**). We were equally delighted to observe a smooth transformation of cyclopropene **1a-9** possessing two substituents on the tether. This transformation allowed the preparation of spiropentane **3a-10** possessing five contiguous stereocenters as a single diastereomer out of 8 possible isomers. The relative configuration was determined by X-ray analysis of an hydrazine-derived from **3a-7** (for details, see Supporting Information), and all the other configurations were assigned by analogy. Interestingly when the stereocenter on the cyclopropenyl ring is inverted as in cyclopropene **1b**, the overall diastereoselectivity of the process is lower. If the intramolecular nucleophilic substitution of the cyclopropyl copper intermediate occurs with pure inversion of configuration, this discrepancy could only originate from a less diastereoselective carbocupration of **1b-1** and **1b-2**, leading to **3b-1** and **3b-2** (compared to **3a-2** and **3a-6**, Scheme 5). This inconsistency suggests that some steric interactions occur in the conformer that has a chelation between the ester, the tosylate, and the organometallic species, leading to minor addition from the opposite diastereotopic face (mismatched isomer; see molecular modeling in the Supporting Information). Following the same logic, similar steric interactions should be observed for the carbometalation of the fully substituted cyclopropene **1a-10** (R^4^ ≠ H). Indeed, the diastereoselectivity of the process is slightly lower (compare **3a-11** with **3a-2**, Scheme 5). This result implies that the presence of an alkyl substituent R^4^ (for **1a-10**, R^4^ = Me) as it was for **1b** provides a certain amount of the opposite diastereomer (see molecular modeling in the Supporting Information). To avoid the formation of this minor isomer, we therefore hypothesized that a substrate devoid of this double chelation should lead to a higher diastereoselective carbometalation reaction and consequently to a better overall selectivity for the formation of spiropentane. Therefore, the secondary chloride **6a-1** was prepared as a single diastereomer by an S_N_2 reaction on the tosylate. Treatment of **6a-1** with the same organocopper and under the same experimental conditions led to the formation of a new spiropentane **3a-12** as a single diastereomer. It should be noted that this diastereomer is different from the two diastereomers of **3a-11**. This underlines the fact that the diastereoselectivity in the formation of **3a-12** is directed exclusively by the ester, without influence of the chloride on the facial diastereocontrol.

**Scheme 5:**
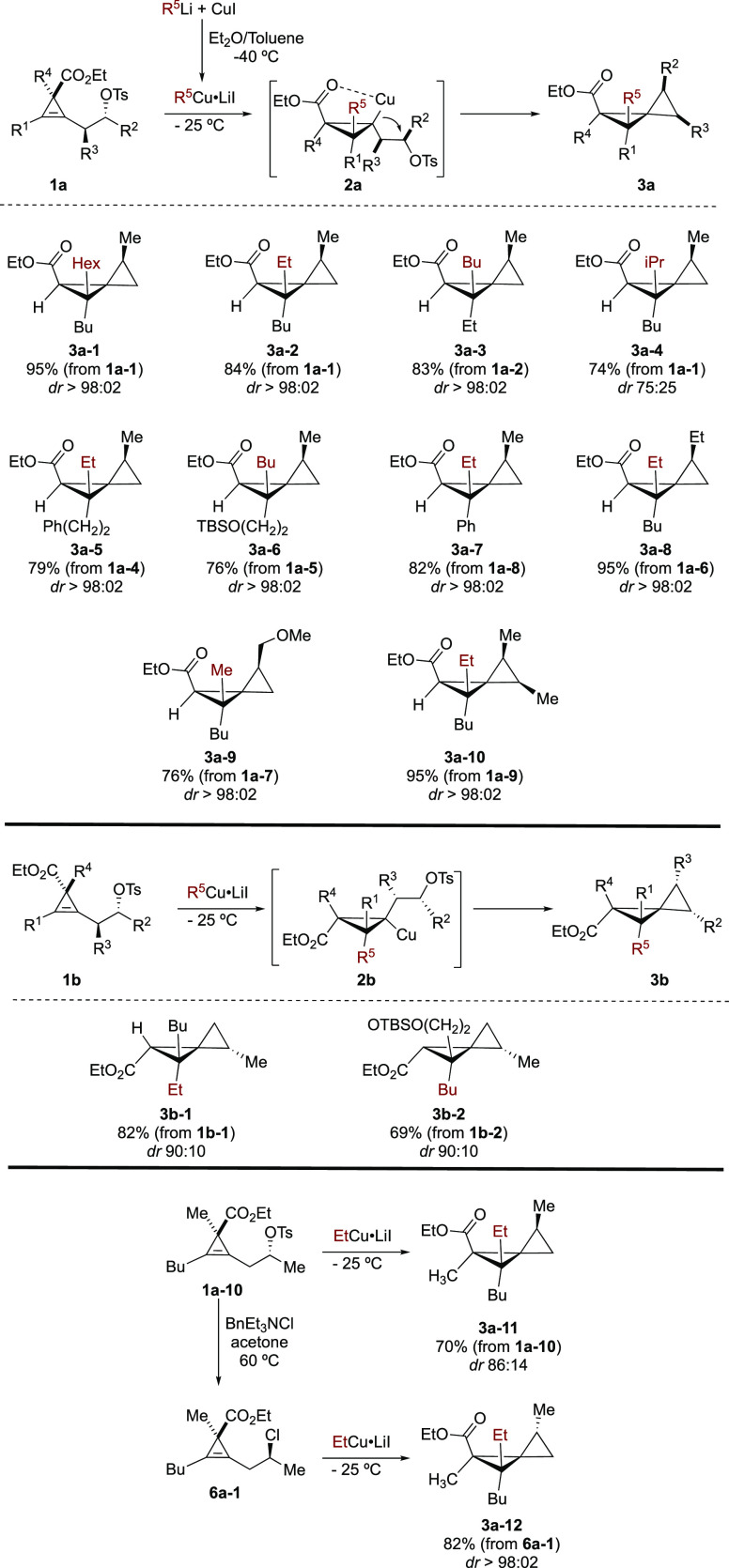
Synthesis of Spiropentanes

An alternative approach to overcome this lower diastereoselectivity would be to have a stronger directing group that would counterbalance the conformational bias of the mismatched isomer. Therefore, a simple reduction of the ester **6b-1** into the strongly chelating alcohol derivative **7b-1** was prepared as described in Scheme 6. The addition of MeCu at low temperature promotes a fully diastereoselective carbometalation reaction followed by a subsequent intramolecular nucleophilic substitution to give **8b-1** as a single diastereomer. The same stereochemical outcome was obtained by using the tosylate **7a-1** as starting material, and in all cases, a single diastereomer of the corresponding spiropentane methanols **8a-1** to **8a-3** was obtained.

**Scheme 6:**
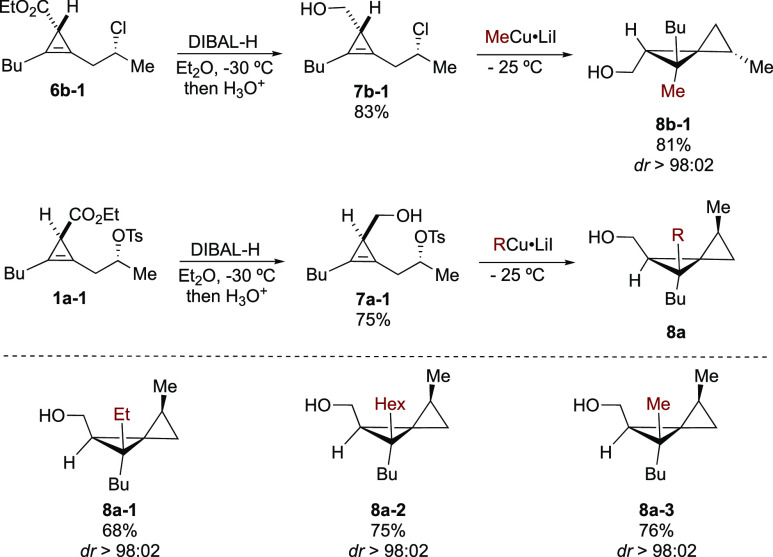
Synthesis of Spiropentane Methanols

In conclusion, by recognizing the potential of directed diastereoselective carbometalation of sp^2^-disubstituted cyclopropene, a new approach to polysubstituted spiropentanes was developed. This reaction requires the combined effect of a *syn*-facial diastereoselective carbometalation with a regio-directing group that serves as a leaving group in a subsequent intramolecular nucleophilic substitution. This method allows the preparation of diverse polysubstituted spiropentanes with up to five stereocenters including three quaternary carbon centers. Preparation of enantiopure spiropentanes are therefore easily accessible by using enantiopure epoxides during the preparation of the starting materials.

## Supporting Information Available

Experimental procedures, characterization data for all new compounds, along with copies of spectra (PDF)
