# Supplementary material for: Stereoselective Synthesis of Polysubstituted Spiropentanes
Source: J Am Chem Soc. 2022 Sep 11;144(37):16732–6. doi: 10.1021/jacs.2c07370 (PMC9501800; doi:10.1021/jacs.2c07370)

Stereoselective Synthesis of Polysubstituted Spiropentanes

Supporting Information

Yair Cohen, Dor Toledano, and Ilan Marek*

Schulich Faculty of Chemistry,
Technion - Israel Institute of Technology
Technion City
Haifa, 32000
Israel
Email: chilanm@technion.ac.il

Table of contents

1	General experimental details	S3
2	Molecular modeling for matched/mismatched isomers	S4
3	Protocols for the synthesis of cyclopropenes **5**	S8
4	Characterization of cyclopropenes **5**	S10
5	Protocols for the synthesis of tosylated cyclopropenes **1**	S14
6	Characterization of tosylated cyclopropenes **1**	S14
7	Protocols for the synthesis of chloro-cyclopropenes **6**	S19
8	Characterization of chlorinated chloro-cyclopropenes **6**	S20
9	Protocols for the synthesis of cyclopropyl methanols **7**	S21
10	Characterization of cyclopropyl methanols **7**	S22
11	Protocols for the synthesis of spiropentanes **3, 8**	S23
12	Characterization of spiropentanes **3, 8**	S24
13	Miscellaneous synthetic protocols	S30
14	Crystal data and structure refinement for compounds **1a-8**, **SI-7**	S37
15	References	S38
16	NMR spectra of starting cyclopropenes **5,1,6**	S40
17	NMR spectra of spiropentanes **3,8**	S68
18	NMR spectra of miscellaneous compounds	S86

1. General experimental details

Unless otherwise stated, reactions were conducted in a flame-dried glassware under a positive pressure of argon. Et_2O and THF were dried from Pure-Solv® Purification System (Innovative Technology©). Toluene was freshly distilled from CaH_2. All organolithium reagents were purchased from Aldrich. BF_3•EtO_2 was purchased in a septum-sealed bottle or was distilled under reduced pressure from CaH_2. All other reagents were purchased from Aldrich, Strem, Acros, and Alfa Aesar, and used as received. All sp^2-monosubstituted cyclopropenes **4** are known compounds and were synthesized according to known procedures and spectral data were compared with authentic samples. Thin-layer chromatography (TLC) was conducted with E. Merck silica gel 60 F254 pre-coated plates, (0.25 mm) and visualized by exposure to UV light (254 nm) or stained with anisaldehyde, CAM (solution of $Mo_7(NH_4)_6O_{24}$) and $(NH_4)_4Ce(SO_4)_4$), phosphomolybdic acid, or potassium permanganate. Column chromatography was performed using Fluka silica gel 60 Å (40- 63 µm, 230-400 mesh). All NMR spectra were recorded on Bruker spectrometers (AVII 300, AVIII 400, AVII500 and AVII 600), and reported relative to deuterated solvent signals and/or $SiMe_4$ as internal standard. Chemical shifts are reported in parts per million (ppm) with respect to the residual solvent signal $CDCl_3$ (^{1}H NMR: δ = 7.26; ^{13}C NMR: δ = 77.16). Peak multiplicities are reported as follows: s = singlet, bs = broad singlet, d = doublet, t = triplet, q = quartet, quin = quintet, sext = sextet, sep= septet, dd = doublet of doublets, td = triplet of doublets, ddd = doublet of doublets of doublets, m = multiplet. High-resolution mass spectra (HRMS) were obtained by the mass spectrometry facility at the Technion.

2. Molecular modeling for matched/mismatched isomers.

While the carbometalation of cyclopropenes **1a** gave outstanding diastereoselectivity, when the stereocenter on the cyclopropenyl ring is inverted as in cyclopropene **1b**, the overall diastereoselectivity of the process is lower (Scheme 1-SI, compare **3a-2** with **3b-1**, and **3a-6** with **3b-2**).

Scheme 1-SI. Different diastereocontrol from different diastereomers

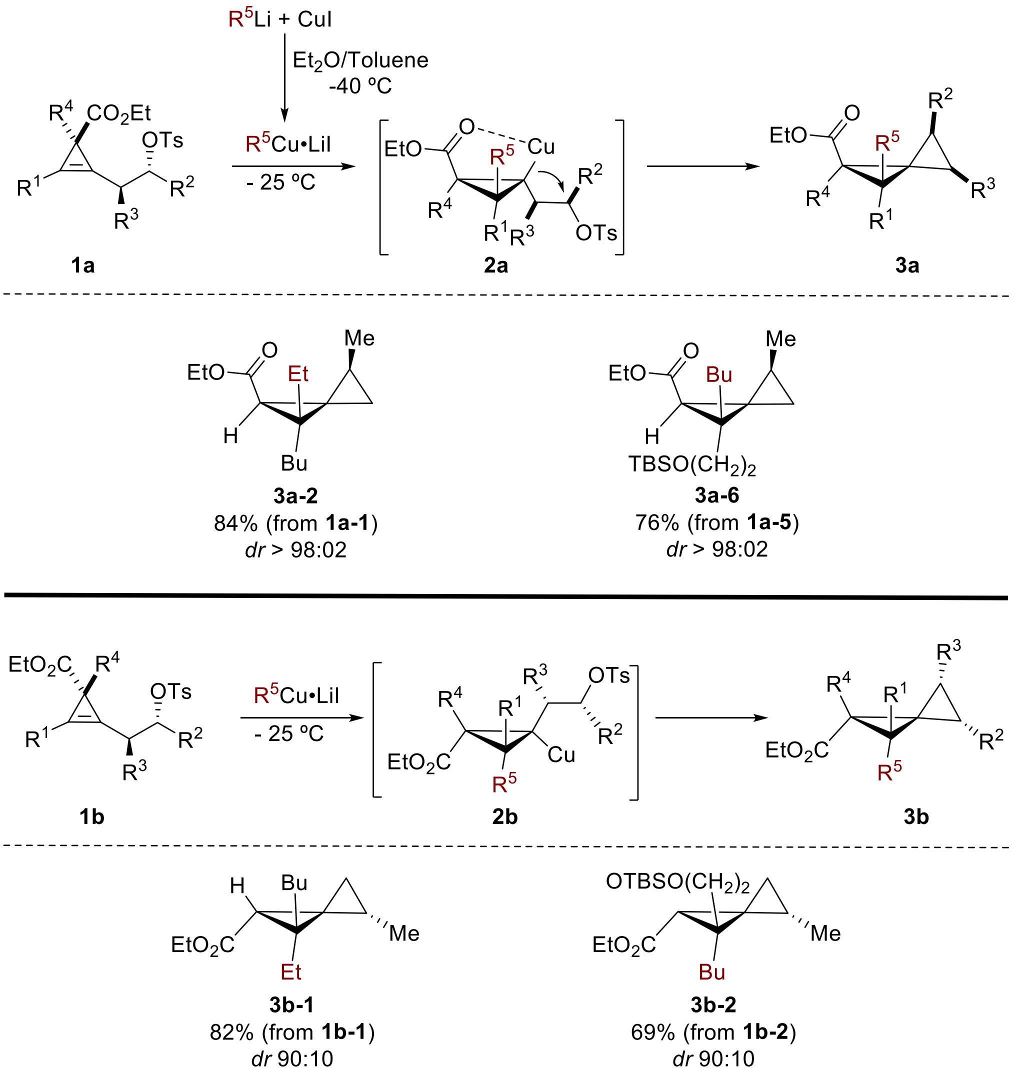

To understand this phenomenon, non-calculated molecular modeling was performed using simple Avocadro system. The modeling for each diastereomer (**1a** and **1b**) was made and different conformations were analyzed. In the first conformation (**1a**) a matched diastereofacial direction of the organocopper by the ester and the tosylate were operative (Scheme 2-SI). In the second conformation, a mismatched effect between the two directing groups appeared. For instance, for cyclopropene **1a-1,** the matched conformation brings no significant steric interactions, while the

mismatched one brings R^1 and R^2 (Scheme 2-SI, cyclopropene **1a-1** represented, R^1 = Bu, R^2 = Me) in close proximity. Therefore, the system prefers to react through the match pair leading to an excellent diastereoselectivity.

Scheme 2-SI. Molecular modeling for cyclopropene 1a-1.

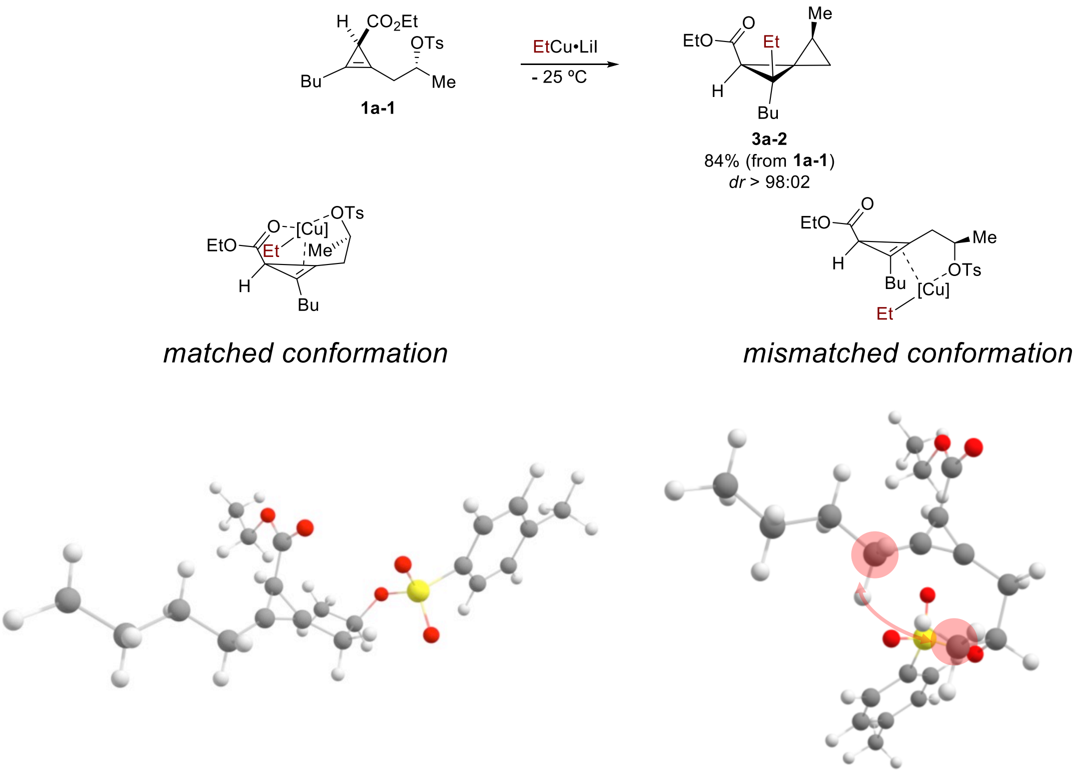

However, for cyclopropene **1b-1** the matched conformation in which the ester and the tosylate direct to the same diastereotopic face of the ring generates steric interactions between R^1 and R^2 (Scheme 3-SI). On the other hand, the mismatched conformation prevents this interaction, therefore promotes the formation of the minor diastereomer.

Scheme 3-SI. Molecular modeling for cyclopropene 1b.

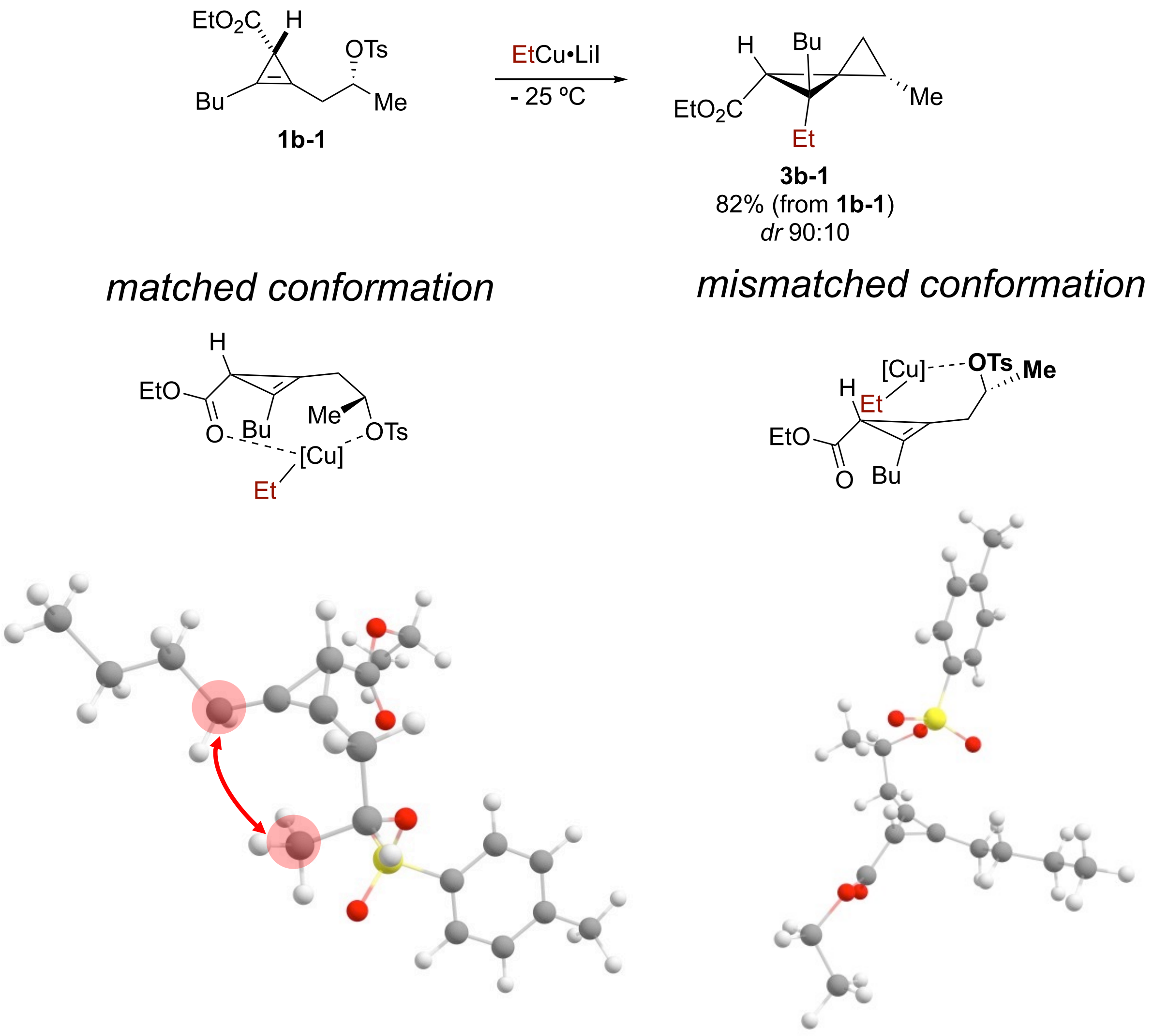

Along the same vein, similar steric interactions were observed for the carbometalation of the fully substituted cyclopropene **1a-10** ($R^4 \neq H$). Indeed, the diastereoselectivity of the process is lower (compare **3a-11** and **3a-2**, Scheme 4-SI). In the matched conformation, steric repulsion is generated between R^4 and R^2 ($R^4 = R^2 = Me$). This negative interaction hampers a complete diastereocontrol of the addition giving rise to the formation of the minor diastereomer.

Scheme 4-SI. Molecular modeling for cyclopropene 1a-10.

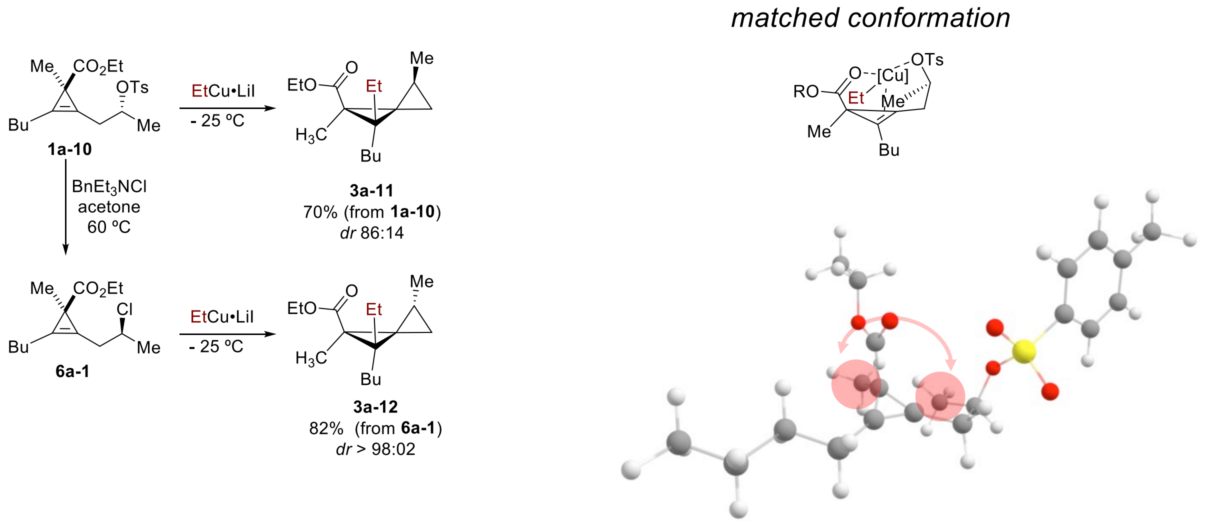

To solve this problem, we therefore considered using a directing group that would deliver a regioselective addition but would not be coordinative to compete with the ester on the facial selection. Indeed, usage of chloride **6a-1** resulted in a clean formation of spiropentane **3a-12**, with three contiguous quaternary centers as a unique diastereomer.

3. Protocols for the synthesis of cyclopropenes **5**

Procedure A: Lithiation-epoxide ring opening sequence.

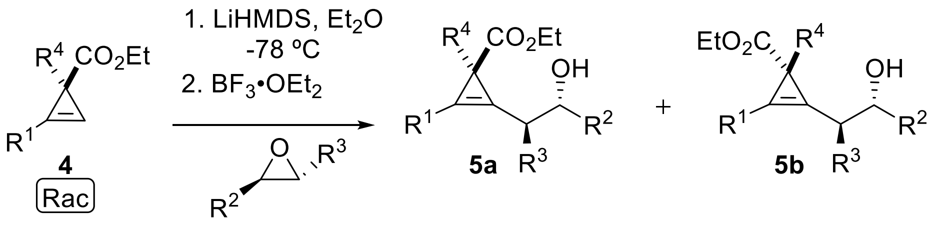

In a flame-dried flask equipped with a thermometer, a solution of cyclopropene **4** (1 equiv.) in Et_2O (0.5 M) was cooled to -80 °C. Commercially available solution of LiHMDS (1.3 equiv.) or freshly prepared from *n*BuLi and HMDS was added dropwise while maintaning the temperature below -60 °C. Once the addition was completed, the reaction mixture was stirred for 1 h still maintaning the temperature between -80 °C to -60 °C. Then, the deep red mixture was cooled again to -80 °C, and BF_3•EtO_2 was added dropwise on the side of the flask, followed by a very slow addition of the epoxide while maintaning the temperature below -60 °C. The reaction was followed by TLC analysis of hydrolyzed aliquots (EtOAc/petroleum ether 20/80, and revealed by $KMnO_4$ or *p*-anisaldehyde) and upon completion (2-4 h), the reaction was quenched with an aqueous saturated solution of NH_4Cl. The aqueous phase was extracted with Et_2O. The combined organic phase were washed with brine, dried over Na_2SO_4 and concentrated under reduced pressure. The crude reaction mixture was purified through column chromatography to provide the two diastereomeric cyclopropenes **5a** and **5b** **.** Typical R_f values (EtOAc/petroleum ether 20/80): R_f(**5a**) = 0.15 - 0.2, R_f(**5b**) = 0.3 - 0.4).

Procedure B: Deprotection of silyl ethers.

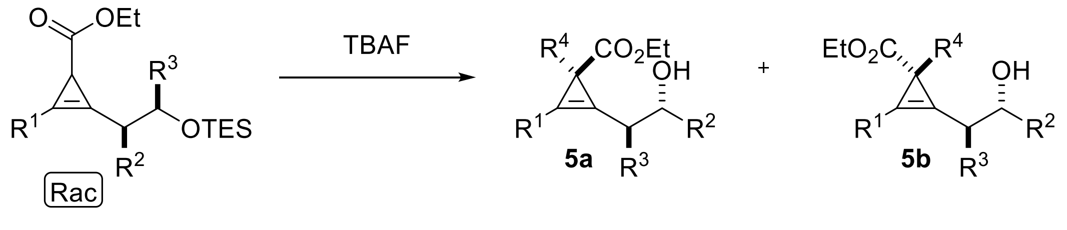

Silyl-protected alcohol (1 equiv.) was dissolved in THF (1 M) in a round bottom flask, open to air. TBAF (1 M sol. In THF, 1.2 equiv.) was added and the reaction mixture was stirred at room temperature until full consumption of the starting material followed by TLC analysis of hydrolyzed aliquots, (Et_2O, *p*-anisaldehyde/$KMnO_4$). The reaction was diluted with Et_2O and quenched with an aqueous saturated solution of NH_4Cl. The aqueous layer was extracted three times with Et_2O, and the combined organic phases were dried over Na_2SO_4, filtered, and concentrated under reduced pressure. The crude mixture

was purified through column chromatography to provide the diastereomeric cyclopropenes **5a** and **5b**. Typical R_f values (EtOAc/petroluem ether 20/80): R_f(**5a**) = 0.15 - 0.2, R_f(**5b**) = 0.3 - 0.4).

4. Characterization of cyclopropenes **5**

(R)-ethyl 2-butyl-3-((R)-2-hydroxypropyl)cycloprop-2-enecarboxylate (5a-1)

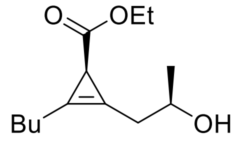

Prepared according to procedure A. **Scale, physical description, yield, mass:** 30 mmol, colorless oil, 31% yield, 2.02 g. **Flash chromatography:** 0 to 20 % EtOAc / petroleum ether. **^{1}H NMR (400 MHz, CDCl$_3$) δ:** 4.14 (qd, J = 7.2, 1.3 Hz, 2H), 4.02 (ddd, J = 7.9, 6.1, 3.8 Hz, 1H), 2.73 (dd, J = 14.9, 3.8 Hz, 1H), 2.51 – 2.38 (m, 3H), 2.12 (s, 1H), 1.53 (quin, J = 7.2 Hz, 2H), 1.43 – 1.30 (m, 2H), 1.25 (t, J = 7.1 Hz, 3H), 1.25 (d, J = 6.3 Hz, 3H), 0.90 (t, J = 7.3 Hz, 3H). **^{13}C NMR (101 MHz, CDCl$_3$) δ** 178.3, 109. 6, 102.7, 66.2, 60.6, 34.8, 29.0, 24.5, 22.7, 22.7, 22.4, 14.4, 13.8. **HRMS** (APCI): m/z calculated for $C_{13}H_{23}O_3$ $[M+H]^+$: 227.1642, found 227.1667.

(S*)-ethyl 2-butyl-3-((R*)-2-hydroxypropyl)cycloprop-2-enecarboxylate (5b-1)

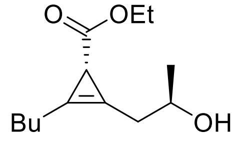

Prepared according to procedure A. **Scale, physical description, yield, mass:** 30 mmol, colorless oil, 17% yield, 1.09 g. **Flash chromatography:** 0 to 20 % EtOAc / petroleum ether. **^{1}H NMR (400 MHz, CDCl$_3$) δ:** 4.15 (q, J = 7.3 Hz, 2H), 3.93 (dqd, J = 9.0, 6.3, 2.8 Hz, 1H), 2.67 (dd, J = 14.5, 9.4 Hz, 1H), 2.49 – 2.36 (m, 3H), 2.08 (s, 1H), 1.53 (quin, J = 7.3 Hz, 2H), 1.43 – 1.29 (m, 2H), 1.27 (t, J = 7.1 Hz, 3H), 1.25 (d, J = 6.3 Hz, 3H), 0.90 (t, J = 7.3 Hz, 3H). **^{13}C NMR (101 MHz, CDCl$_3$) δ:** 178.7, 110.0, 102.0, 64.6, 60.7, 35.3, 28.9, 24.3, 22.3, 22.1, 22.1, 14.3, 13.8. **HRMS** (APCI): m/z calculated for $C_{13}H_{23}O_3$ $[M+H]^+$: 227.1642, found 227.1656.

(R)-ethyl 2-ethyl-3-((R)-2-hydroxypropyl)cycloprop-2-enecarboxylate (5a-2)

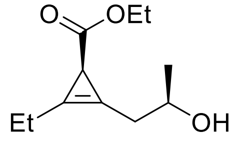

Prepared according to procedure A. **Scale, physical description, yield, mass:** 19 mmol, colorless oil, 24% yield, 916 mg. **Flash chromatography:** 0 to 20 % EtOAc / petroleum ether. **^{1}H NMR (400 MHz, CDCl$_3$) δ:** 4.14 (qd, J = 7.2, 2.4 Hz, 2H), 4.03 (s, 1H), 3.12 (s, 1H), 2.74 (ddd, J = 15.0, 3.3, 1.4 Hz, 1H), 2.45 (qd, J = 7.9, 5.8 Hz, 3H), 2.14 (s, 1H), 1.26 (t, J = 7.1 Hz, 3H), 1.25 (d, J = 6.3 Hz, 3H), 1.15 (t, J = 7.5 Hz, 3H). **^{13}C NMR (101 MHz, CDCl$_3$) δ:** 178.3, 110.6, 102.4, 66.3, 60.6, 34.7, 22.6, 18.4, 14.4, 11.5. **HRMS** (APCI): m/z calculated for $C_{11}H_{29}O_3$ $[M+H]^+$: 199.1329, found 199.1302.

(S)-ethyl 2-cyclohexyl-3-((R)-2-hydroxypropyl)cycloprop-2-enecarboxylate (5a-3)

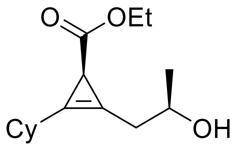

*Note: compound **5a-3** was isolated along with an unknown impurity as could be seen in ^1H NMR spectrum around 3.5 ppm.*

Prepared according to procedure A. **Scale, physical description, yield, mass:** 16 mmol, colorless oil, 17% yield, 645 mg. **Flash chromatography:** 0 to 20 % EtOAc / petroleum ether. **^{1}H NMR (400 MHz, $CDCl_3$) δ:** 4.14 (qd, J = 7.1, 1.5 Hz, 2H), 4.02 (dtd, J = 8.1, 3.7, 1.9 Hz, 1H), 2.74 (ddd, J = 14.8, 3.7, 1.2 Hz, 1H), 2.51 (s, 1H), 2.50 – 2.36 (m, 1H), 2.13 (s, 1H), 1.91 – 1.77 (m, 2H), 1.73 – 1.63 (m, 3H), 1.63 – 1.56 (m, 1H), 1.38 – 1.29 (m, 4H), 1.25 (d, *J* = 6.3 Hz, 3H), 1.25 (t, J = 7.1 Hz, 3H). **^{13}C NMR (101 MHz, $CDCl_3$) δ:** 178.5, 113.2, 101.8, 66.4, 60.6, 34.9, 34.3, 30.4, 26.0, 25.2, 22.6, 22.2, 14.4. **HRMS** (APCI): m/z calculated for $C_{15}H_{25}O_3$ $[M+H]^+$: 253.1804, found 253.1808.

(R)-ethyl 2-((R)-2-hydroxypropyl)-3-phenethylcycloprop-2-enecarboxylate (5a-4)

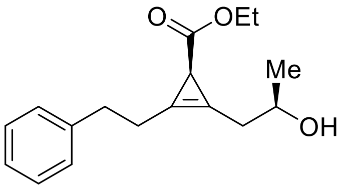

Prepared according to procedure A. **Scale, physical description, yield, mass:** 15 mmol, colorless oil, 18 % yield, 750 mg. **Flash chromatography:** 0 to 20 % EtOAc / petroleum ether. **^{1}H NMR (400 MHz, $CDCl_3$) δ:** 7.33 – 7.27 (m, 2H), 7.21 (td, J = 6.2, 1.7 Hz, 3H), 4.22 – 4.05 (m, 3H), 3.87 (s, 1H), 2.95 – 2.82 (m, 2H), 2.83 – 2.74 (m, 2H), 2.58 (ddt, J = 14.8, 3.8, 1.2 Hz, 1H), 2.44 – 2.33 (m, 1H), 2.14 (s, 1H), 1.26 (t, J = 7.1 Hz, 3H), 1.20 (d, J = 6.3 Hz, 3H). **^{13}C NMR (101 MHz, $CDCl_3$) δ** 178.1, 140.7, 128.5, 128.3, 126.3, 108.57, 103.9, 66.1, 60.6, 34.8, 33.0, 26.4, 22.8, 22.6, 14.4. **HRMS** (APCI): m/z calculated for $C_{17}H_{23}O_3$ $[M+H]^+$: 275.1957, found 275.1657.

(R)-ethyl 2-(2-((tert-butyldimethylsilyl)oxy)ethyl)-3-((R)-2-hydroxypropyl)cycloprop-2-enecarboxylate (5a-5)

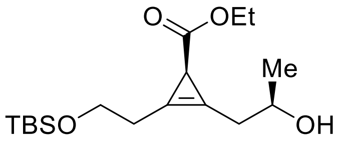

Prepared according to procedure A. **Scale, physical description, yield, mass:** 15 mmol, colorless oil, 14% yield, 707mg. **Flash chromatography:** 0 to 20 % EtOAc / petroleum ether. **^{1}H NMR (400 MHz, $CDCl_3$) δ:** 4.13 (qq, J = 6.9, 3.7 Hz, 2H), 4.02 (s, 1H), 3.82 (tt, J = 6.5, 3.4 Hz, 2H), 3.25 (d, J = 4.4 Hz, 1H), 2.77 – 2.56 (m, 2H), 2.48 (ddt, J = 15.1, 7.9, 1.2 Hz, 1H), 2.13 (s, 1H), 1.25 (t, J = 7.1 Hz, 3H), 1.25 (d, J = 6.3 Hz, 3H), 0.89 (s, 9H), 0.06 (d, J = 1.9 Hz, 6H). **^{13}C NMR (101 MHz, $CDCl_3$) δ:** 177.8, 106.6, 104.5, 66.0,

60.5, 60.5, 35.0, 28.8, 25.9, 22.7, 22.4, 18.3, 14.4, -5.28, -5.32. **HRMS** (APCI): m/z calculated for $C_{17}H_{33}O_4Si$ $[M+H]^+$: 329.2143, found 329.2136.

(S)-ethyl 2-(2-((tert-butyldimethylsilyl)oxy)ethyl)-3-((R)-2-hydroxypropyl)cycloprop-2-enecarboxylate (5b-2)

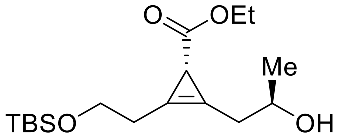

Prepared according to procedure A. **Scale, physical description, yield, mass:** 15 mmol, colorless oil, 15% yield, 740mg. **Flash chromatography:** 0 to 20 % EtOAc / petroleum ether. **^{1}H NMR (400 MHz, $CDCl_3$) δ:** 4.14 (ddq, J = 10.9, 7.0, 3.6 Hz, 2H), 3.96 (td, J = 6.2, 3.2 Hz, 1H), 3.81 (td, J = 6.5, 2.3 Hz, 2H), 3.70 (d, J = 3.7 Hz, 1H), 2.73 – 2.59 (m, 2H), 2.51 – 2.41 (m, 1H), 2.11 (s, 1H), 1.27 (t, J = 7.1 Hz, 3H), 1.25 (d, J = 6.3 Hz, 3H), 0.88 (s, 9H), 0.06 (d, J = 2.4 Hz, 6H). **^{13}C NMR (101 MHz, $CDCl_3$) δ** 178.3, 107.0, 104.0, 64.8, 60.7, 60.5, 35.4, 28.6, 25.9, 22.3, 21.9, 18.3, 14.3, -5.29, -5.31. **HRMS** (APCI): m/z calculated for $C_{17}H_{33}O_4Si$ $[M+H]^+$: 329.2143, found 329.2140.

(R)-ethyl 2-butyl-3-((R)-2-hydroxybutyl)cycloprop-2-enecarboxylate (5a-6)

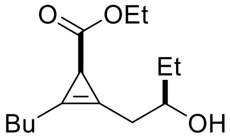

Prepared according to procedure A. **Scale, physical description, yield, mass:** 15 mmol, colorless oil, 18% yield, 660 mg. **Flash chromatography:** 0 to 20 % EtOAc / petroleum ether. **^{1}H NMR (400 MHz, $CDCl_3$) δ:** 4.22 – 4.06 (m, 3H), 3.73 (s, 1H), 2.80 – 2.70 (m, 1H), 2.52 – 2.38 (m, 3H), 2.12 (s, 1H), 1.53 (dddd, J = 12.9, 8.7, 6.1, 3.4 Hz, 4H), 1.42 – 1.32 (m, 2H), 1.25 (t, J = 7.1 Hz, 3H), 0.97 (t, J = 7.5 Hz, 3H), 0.91 (t, J = 7.3 Hz, 3H). **^{13}C NMR (101 MHz, $CDCl_3$) δ** 178.3, 109.5, 102.8, 71.4, 60.5, 32.7, 29.6, 29.0, 24.4, 22.7, 22.4, 14.4, 13.8, 10.2. **HRMS** (APCI): m/z calculated for $C_{14}H_{25}O_3$ $[M+H]^+$: 241.1804, found 241.1803.

(R)-ethyl 2-butyl-3-((S)-2-hydroxy-3-methoxypropyl)cycloprop-2-enecarboxylate (5a-7)

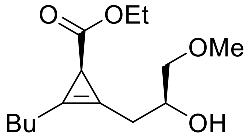

Note: an unknown impurity is present in the sample as indicated by additional ^{13}C NMR signals.

Prepared according to procedure A. **Scale, physical description, yield, mass:** 22 mmol, colorless oil, 29% yield, 1.05 g. **Flash chromatography:** 0 to 20 % EtOAc / petroleum ether. **^{1}H NMR (400 MHz, $CDCl_3$) δ:** 4.13 (qd, J = 7.1, 1.2 Hz, 2H), 3.99 (brs, 1H), 3.43 (dt, J = 10.3, 5.3 Hz, 2H), 3.39 (s, 3H), 3.21 (brs, 1H), 2.75 (dd, J = 15.1, 5.0 Hz, 1H), 2.57 (dd, J = 15.1, 7.5 Hz, 1H), 2.42 (t, J = 7.3 Hz, 2H), 2.11 (s, 1H), 1.58 – 1.48 (m, 2H), 1.39 – 1.31 (m, 2H), 1.25 (t, J = 7.1 Hz, 3H), 0.90 (t, J = 7.3 Hz, 3H). **^{13}C NMR**

(101 MHz, $CDCl_3$) δ: 178.0, 109.4, 102.2, 75.9, 68.6, 60.4, 59.2, 36.7, 29.5, 29.0, 24.4, 22.4, 14.4, 13.8. **HRMS** (APCI): m/z calculated for $C_{14}H_{25}O_4$ $[M+H]^+$: 257.1753, found 257.1769.

(S)-ethyl 2-((S)-2-hydroxy-3-methoxypropyl)-3-phenylcycloprop-2-enecarboxylate (5a-8)

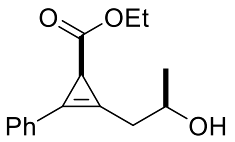

Prepared according to procedure B. **Scale, physical description, yield, mass:** 14.7 mmol, colorless oil, 35% yield, 1.25 g. **Flash chromatography:** 0 to 20 % EtOAc / petroleum ether. **^{1}H NMR (400 MHz, $CDCl_3$) δ:** 7.53 – 7.46 (m, 2H), 7.41 (tt, J = 6.5, 1.1 Hz, 2H), 7.37 – 7.31 (m, 1H), 4.26 – 4.14 (m, 3H), 3.16 (s, 1H), 3.00 (dd, J = 15.1, 3.8 Hz, 1H), 2.74 (dd, J = 15.2, 8.1 Hz, 1H), 2.53 (s, 1H), 1.34 (d, J = 6.3 Hz, 3H), 1.28 (t, J = 7.1 Hz, 3H). **^{13}C NMR (101 MHz, $CDCl_3$) δ:** 177.1, 129.5, 129.1, 128.8, 126.7, 107.6, 106.9, 66.3, 60.9, 35.7, 22.9, 22.4, 14.4. **HRMS** (APCI): m/z calculated for $C_{15}H_{17}O_3$ $[M-H]^+$: 245.1178, found 245.1210.

(R)-ethyl 2-butyl-3-((2S,3R)-3-hydroxybutan-2-yl)cycloprop-2-enecarboxylate (5a-9)

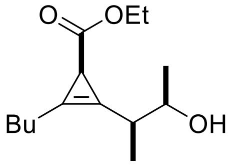

Prepared according to procedure B. **Scale, physical description, yield, mass:** 3.6 mmol, colorless oil, 32% yield, 284 mg. **Flash chromatography:** 0 to 20 % EtOAc / petroleum ether. **^{1}H NMR (400 MHz, $CDCl_3$) δ:** 4.15 (qd, J = 7.1, 2.9 Hz, 2H), 3.70 (dd, J = 12.1, 6.1 Hz, 1H), 3.02 (d, J = 6.1 Hz, 1H), 2.63 – 2.52 (m, 1H), 2.44 (ddd, J = 8.0, 6.6, 1.2 Hz, 2H), 2.16 (s, 1H), 1.59 – 1.47 (m, 2H), 1.42 – 1.30 (m, 2H), 1.33 – 1.11 (m, 9H), 0.91 (t, J = 7.3 Hz, 3H). **^{13}C NMR (101 MHz, $CDCl_3$) δ:** 178.4, 109.9, 106.8, 70.9, 60.6, 39.6, 29.2, 24.4, 23.3, 22.4, 21.1, 16.7, 14.4, 13.8. **HRMS** (APCI): m/z calculated for $C_{14}H_{25}O_3$ $[M+H]^+$: 241.1804, found 241.1807.

(R)-ethyl 2-butyl-3-((R)-2-hydroxypropyl)-1-methylcycloprop-2-enecarboxylate (5a-10)

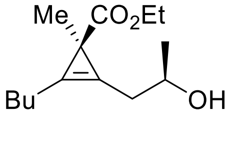

Prepared according to procedure A. **Scale, physical description, yield, mass:** 15 mmol, colorless oil, 15% yield, 560 mg. **Flash chromatography:** 0 to 20 % EtOAc / petroleum ether. **^{1}H NMR (400 MHz, $CDCl_3$) δ:** 4.11 (qd, J = 7.1, 1.5 Hz, 2H), 4.01 (s, 1H), 3.13 (d, J = 4.1 Hz, 1H), 2.75 – 2.65 (m, 1H), 2.48 – 2.35 (m, 3H), 1.57 – 1.43 (m, 2H), 1.37 (ddd, J = 9.6, 7.6, 6.1 Hz, 2H), 1.31 (s, 3H), 1.29 – 1.14 (m, 6H), 0.91 (t, J = 7.3 Hz, 3H). **^{13}C NMR (101 MHz, $CDCl_3$) δ:** 179.0, 115.2, 108.4, 66.2, 60.7, 33.9, 29.2, 26.8, 23.7, 22.7, 22.5, 19.0, 14.4, 13.8. **HRMS** (APCI): m/z calculated for $C_{14}H_{25}O_3$ $[M+H]^+$: 241.1804, found 241.1798.

5. Protocols for the synthesis of tosylated cyclopropenes **1**

Procedure C: Tosylation of secondary alcohol.

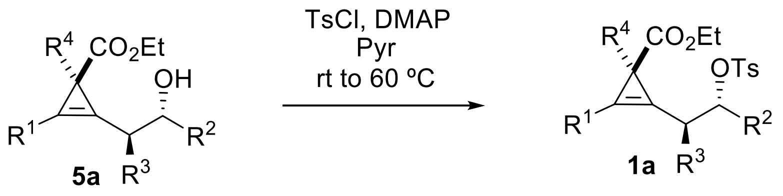

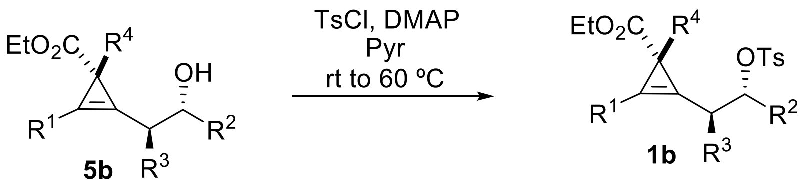

In a round bottom flask, open to air, cyclopropenes **5a** and **5b** (1equiv.) were dissolved in pyridine (0.5 M). DMAP (0.1 equiv.) and tosyl chloride (1.5 equiv.) were added sequentially. The reaction was stirred at room temperature and followed by TLC analysis of hydrolyzed aliquots (EtOAc/petroleum ether 20/80, $KMnO_4$ or *p*-anisaldehyde). When no coversion was observed, the reaction was heated to 60 °C overnight. Upon completion, the reaction mixture was poured into a solution of Et_2O/ 1 M HCl (2/1). The aqueous phase was extracted with Et_2O. The combined organic phases were washed with brine, dried over Na_2SO_4 and concentrated under reduced pressure. The crude mixture was purified through column chromatography to provide pure cyclopropenes **1a** and **1b**.

6. Characterization of tosylated cyclopropenes **1**

(R)-ethyl 2-butyl-3-((R)-2-(tosyloxy)propyl)cycloprop-2-enecarboxylate (1a-1)

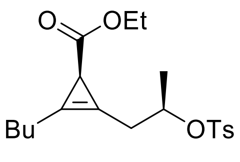

Prepared according to procedure C. **Scale, physical description, yield, mass:** 4.5 mmol, light yellow oil, 60% yield, 964 mg. **Flash chromatography:** 0 to 15 % EtOAc / petroleum ether. **^{1}H NMR (400 MHz, $CDCl_3$) δ:** 7.77 (d, J = 8.3 Hz, 2H), 7.33 (d, J = 8.1 Hz, 2H), 4.78 (dq, J = 12.8, 6.3 Hz, 1H), 4.08 (q, J = 7.1 Hz, 2H), 2.79 – 2.62 (m, 2H), 2.44 (s, 3H), 2.38 (t, J = 7.3 Hz, 2H), 1.93 (s, 1H), 1.48 (dd, J = 8.9, 7.0 Hz, 2H), 1.33 (m, 3H), 1.33 (d, J = 6.3 Hz, 3H), 1.22 (t, J = 7.1 Hz, 3H), 0.89 (t, J = 7.3 Hz, 3H). **^{13}C NMR (101 MHz, $CDCl_3$) δ**: 176.3, 144.7, 134.3, 129.8, 127.7, 109.8, 100.6, 77.0, 60.0, 32.0, 28.8, 24.2, 22.4, 22.0, 21.7, 20.6, 14.4, 13.8. **HRMS** (APCI): m/z calculated for $C_{20}H_{29}O_5S$ $[M+H]^+$: 381.1730, found 381.1745.

(S)-ethyl 2-butyl-3-((R)-2-(tosyloxy)propyl)cycloprop-2-enecarboxylate (1b-1)

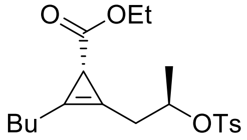

Prepared according to procedure C. **Scale, physical description, yield, mass:** 2.3 mmol, colorless oil, 41% yield, 360 mg. **Flash chromatography:** 0 to 15 % EtOAc / petroleum ether. **^{1}H NMR (400 MHz, $CDCl_3$) δ:** 7.79 (d, J = 8.4 Hz, 2H), 7.33 (d, J = 7.9 Hz, 2H), 4.81 (td, J = 6.6, 5.3 Hz, 1H), 4.08 (q, J = 7.1 Hz, 2H), 2.75 – 2.66 (m, 2H), 2.44 (s, 3H), 2.39 (t, J = 7.3 Hz, 1H), 2.02 (s, 1H), 1.56 – 1.42 (m, 2H), 1.37 – 1.31 (m, 2H), 1.29 (d, J = 6.3 Hz, 3H), 1.22 (t, J = 7.1 Hz, 3H), 0.89 (t, J = 7.3 Hz, 3H). **^{13}C NMR (101 MHz, $CDCl_3$) δ:** 176.2, 144.7, 134.4, 129.8, 127.7, 109.8, 100.7, 77.2, 60.0, 31.9, 28.8, 24.2, 22.4, 22.3, 21.7, 20.5, 14.4, 13.8. **HRMS** (APCI): m/z calculated for $C_{20}H_{29}O_5S$ $[M+H]^+$: 381.1730, found 381.1740.

(R)-ethyl 2-ethyl-3-((R)-2-(tosyloxy)propyl)cycloprop-2-enecarboxylate (1a-2)

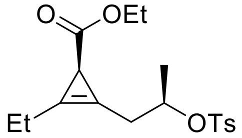

Prepared according to procedure C. **Scale, physical description, yield, mass:** 4.6 mmol, light yellow oil, 47% yield, 755 mg. **Flash chromatography:** 0 to 15 % EtOAc / petroleum ether. **^{1}H NMR (400 MHz, $CDCl_3$) δ:** 7.77 (d, J = 8.3 Hz, 2H), 7.33 (d, J = 7.8 Hz, 2H), 4.77 (pd, J = 6.4, 5.1 Hz, 1H), 4.08 (q, J = 7.1 Hz, 2H), 2.76 (ddt, J = 15.9, 5.3, 1.3 Hz, 1H), 2.66 (ddt, J = 15.8, 6.8, 1.5 Hz, 1H), 2.44 (s, 3H), 2.43 – 2.35 (m, 2H), 1.94 (s, 1H), 1.33 (d, J = 6.3 Hz, 3H), 1.22 (t, J = 7.1 Hz, 3H), 1.09 (t, J = 7.5 Hz, 3H). **^{13}C NMR (101 MHz, $CDCl_3$) δ**: 176.4, 144.7, 134.3, 129.8, 127.7, 110.8, 100.2, 77.0 60.0, 31.9, 21.9, 21.7, 20.6, 18.2, 14.4, 11.3. **HRMS** (APCI): m/z calculated for $C_{18}H_{25}O_5S$ $[M+H]^+$: 353.1417, found 353.1446.

(S)-ethyl 2-cyclohexyl-3-((R)-2-(tosyloxy)propyl)cycloprop-2-enecarboxylate (1a-3)

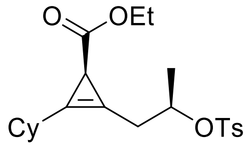

*Note: compound **1a-3** was isolated along with an unknown impurity as shown in ^{1}H NMR spectrum around 2.5 ppm.*

Prepared according to procedure C. **Scale, physical description, yield, mass:** 2.7 mmol, light yellow oil, 62% yield, 680 mg. **Flash chromatography:** 0 to 15% EtOAc / petroleum ether. **^{1}H NMR (400 MHz, $CDCl_3$) δ:** 7.77 (d, J = 8.3 Hz, 2H), 7.33 (d, J = 7.9 Hz, 2H), 4.82 – 4.70 (m, 1H), 4.07 (q, J = 7.1 Hz, 2H), 2.77 (ddd, J = 15.5, 5.2, 1.2 Hz, 1H), 2.67 (ddd, J = 15.4, 7.1, 1.3 Hz, 1H), 2.44 (s, 3H), 1.94 (s, 1H), 1.82 – 1.70 (m, 2H), 1.68 – 1.54 (m, 5H), 1.33 (d, J = 6.3 Hz, 3H), 1.31 – 1.24 (m, 4H), 1.21 (t, J = 7.1 Hz, 3H). **^{13}C NMR (101 MHz, $CDCl_3$) δ:** 176.5, 144.7, 134.3, 129.8, 127.7, 113.5, 99.5, 77.0, 56.0, 34.1, 32.0,

30.3, 30.2, 25.9, 25.3, 25.2, 21.7, 21.5, 20.6, 14.4. **HRMS** (APCI): m/z calculated for $C_{22}H_{31}O_5S$ [M+H]$^+$: 407.1892, found 407.1888.

(R)-ethyl 2-phenethyl-3-((R)-2-(tosyloxy)propyl)cycloprop-2-enecarboxylate (1a-4)

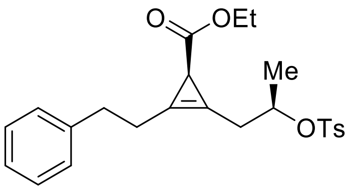

Prepared according to procedure C. **Scale, physical description, yield, mass:** 2.7 mmol, light yellow oil, 55% yield, 61 mg. **Flash chromatography:** 0 to 15% EtOAc / petroleum ether. **^{1}H NMR (400 MHz, CDCl$_3$) δ:** 7.76 (d, J = 8.3 Hz, 2H), 7.36 – 7.27 (m, 3H), 7.24 – 7.14 (m, 4H), 4.72 (td, J = 6.6, 5.2 Hz, 1H), 4.08 (q, J = 7.1 Hz, 2H), 2.90 – 2.70 (m, 4H), 2.67 (dd, J = 15.8, 5.1 Hz, 1H), 2.63 – 2.54 (m, 1H), 1.24 (d, J = 6.3 Hz, 3H), 1.22 (t, J = 7.1 Hz, 3H). **^{13}C NMR (101 MHz, CDCl$_3$) δ**: 176.1, 144.6, 140.7, 134.3, 129.8, 128.5, 128.3, 127.7, 126.2, 109.0, 101.6, 60.1, 32.7, 31.9, 26.2, 22.1, 21.6, 20.5, 14.4. **HRMS** (APCI): m/z calculated for $C_{24}H_{29}O_5S$ [M+H]$^+$: 429.1730, found 429.1742.

(R)-ethyl 2-(2-((tert-butyldimethylsilyl)oxy)ethyl)-3-((R)-2-(tosyloxy)propyl)cycloprop-2-enecarboxylate (1a-5)

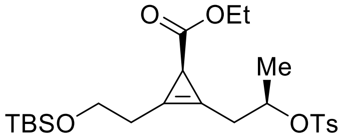

Prepared according to procedure C. **Scale, physical description, yield, mass:** 2.3 mmol, colorless oil, 51% yield, 559 mg. **Flash chromatography:** 0 to 15 % EtOAc / petroleum ether. **^{1}H NMR (400 MHz, CDCl$_3$) δ:** 7.78 (d, J = 8.3 Hz, 2H), 7.33 (d, 2H), 4.78 (ddd, J = 6.9, 6.2, 4.9 Hz, 1H), 4.08 (q, J = 7.1 Hz, 2H), 3.74 (t, J = 6.8 Hz, 2H), 2.81 – 2.55 (m, 4H), 2.44 (s, 3H), 1.97 (s, 1H), 1.32 (d, J = 6.3 Hz, 3H), 1.22 (t, J = 7.1 Hz, 3H), 0.87 (s, 9H), 0.04 (d, J = 1.5 Hz, 6H). **^{13}C NMR (101 MHz, CDCl$_3$) δ**: 176.1, 144.7, 134.3, 129.8, 127.7, 107.2, 102.3, 76.9, 60.2, 60.1, 32.0, 28.4, 25.9, 21.8, 21.7, 20.6, 18.2, 14.4, -5.32, -5.34**. HRMS:** (APCI): m/z calculated for $C_{24}H_{39}O_6SSi$ [M+H]+: 483.2231, found 483.2214.

(S)-ethyl 2-(2-((tert-butyldimethylsilyl)oxy)ethyl)-3-((R)-2-(tosyloxy)propyl)cycloprop-2-enecarboxylate (1b-2)

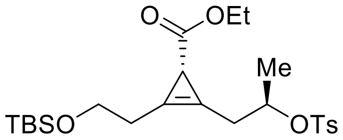

Prepared according to procedure C. **Scale, physical description, yield, mass:** 2.2 mmol, light yellow oil, 45% yield, 487 mg. **Flash chromatography:** 0 to 15 % EtOAc / petroleum ether. **^{1}H NMR (400 MHz, CDCl$_3$) δ:** 7.79 (d, J = 8.4 Hz, 2H), 7.33 (d, J = 7.9 Hz, 2H), 4.82 (td, J = 6.7, 5.2 Hz, 1H), 4.08 (q, J = 7.1 Hz, 2H), 3.74 (td, J = 6.7, 1.7 Hz, 2H), 2.73 (ddd, J = 5.0, 3.2, 1.4 Hz, 2H), 2.68 – 2.55 (m, 2H), 2.44 (s,

3H), 2.05 (s, 1H), 1.29 (d, J = 6.3 Hz, 3H), 1.22 (t, J = 7.1 Hz, 3H), 0.87 (s, 9H), 0.04 (d, J = 1.5 Hz, 6H). **^{13}C NMR (101 MHz, CDCl$_3$) δ**: 176.0, 144.7, 134.4, 129.8, 127.7, 107.2, 102.3, 60.3, 60.1, 31.9, 28.4, 25.9, 22.0, 21.7, 20.5, 18.2, 14.4, -5.32, -5.34. **HRMS** (APCI): m/z calculated for $C_{24}H_{39}O_6SSi$ [M+H]+: 483.2231, found 483.2209.

(R)-ethyl 2-butyl-3-((R)-2-(tosyloxy)butyl)cycloprop-2-enecarboxylate (1a-6)

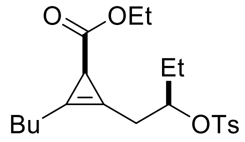

Prepared according to procedure C. **Scale, physical description, yield, mass:** 2.7 mmol, colorless oil, 42% yield, 448 mg. **Flash chromatography:** 0 to 15 % EtOAc / petroleum ether. **^{1}H NMR (400 MHz, CDCl$_3$) δ:** 7.78 (d, J = 8.3 Hz, 2H), 7.32 (d, 2H), 4.63 (tt, J = 6.6, 5.2 Hz, 1H), 4.09 (q, J = 7.1 Hz, 2H), 2.73 (dq, J = 4.7, 1.5 Hz, 2H), 2.44 (s, 3H), 2.41 – 2.30 (m, 2H), 1.96 (s, 1H), 1.77 – 1.61 (m, 2H), 1.47 (dtd, J = 8.8, 7.2, 5.6 Hz, 2H), 1.39 – 1.27 (m, 2H), 1.22 (t, J = 7.1 Hz, 3H), 0.89 (t, J = 7.3 Hz, 3H), 0.83 (t, J = 7.4 Hz, 3H). **^{13}C NMR (101 MHz, CDCl$_3$) δ**: 176.4, 144.6, 134.3, 129.8, 127.7, 109.7, 100.6, 81.7, 60.0, 29.5, 28.8, 27.0, 24.2, 22.4, 22.0, 21.7, 14.4, 13.8, 9.2. **HRMS** (APCI): m/z calculated for $C_{11}H_{31}O_5S$ $[M+H]^+$: 395.1892, found 395.1913.

(R)-ethyl 2-butyl-3-((S)-3-methoxy-2-(tosyloxy)propyl)cycloprop-2-enecarboxylate (1a-7)

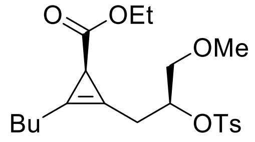

Prepared according to procedure C. **Scale, physical description, yield, mass:** 4.5 mmol, light yellow oil, 55% yield, 1.02 g. **Flash chromatography:** 0 to 15 % EtOAc / petroleum ether. **^{1}H NMR (400 MHz, CDCl$_3$) δ:** 7.78 (d, J = 8.4 Hz, 2H), 7.32 (d, J = 8.1 Hz, 2H), 4.81 – 4.69 (m, 1H), 4.09 (q, J = 7.1 Hz, 2H), 3.53 (dd, J = 10.9, 5.1 Hz, 1H), 3.47 (dd, J = 10.9, 4.2 Hz, 1H), 3.24 (s, 3H), 2.85 (dd, J = 15.7, 7.1 Hz, 1H), 2.77 (dd, J = 15.8, 5.3 Hz, 1H), 2.44 (s, 3H), 2.37 (t, J = 7.3 Hz, 2H), 1.94 (s, 1H), 1.48 (dd, J = 8.7, 7.2 Hz, 2H), 1.33 (dt, J = 8.1, 7.1 Hz, 2H), 1.23 (t, J = 7.1 Hz, 3H), 0.89 (t, J = 7.2 Hz, 3H). **^{13}C NMR (101 MHz, CDCl$_3$) δ**: 176.3, 144.7, 134.1, 129.7, 127.9, 110.1, 100.4, 78.1, 72.5, 60.1, 59.2, 28.8, 27.0, 24.2, 22.4, 22.0, 21.7, 14.4, 13.8. **HRMS** (APCI): m/z calculated for $C_{21}H_{31}O_6S$ $[M+H]^+$: 411.1841, found 411.1836.

(S)-ethyl 2-phenyl-3-((R)-2-(tosyloxy)propyl)cycloprop-2-enecarboxylate (1a-8)

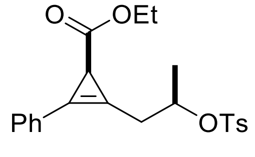

Prepared according to procedure C. **Scale, physical description, yield, mass:** 4.2 mmol, colorless solid, 82% yield, 1.32 g. **Flash chromatography:** 0 to 15 % EtOAc / petroleum ether. **^{1}H NMR (400 MHz,**

CDCl₃) δ: 7.75 (d, J = 8.4 Hz, 2H), 7.47 – 7.33 (m, 5H), 7.26 (d, J = 7.9 Hz, 2H), 4.97 – 4.85 (m, 1H), 4.20 – 4.03 (m, 2H), 3.02 (dd, J = 16.0, 5.1 Hz, 1H), 2.93 (dd, J = 16.0, 6.4 Hz, 1H), 2.41 (s, 3H), 2.26 (s, 1H), 1.44 (d, J = 6.3 Hz, 3H), 1.22 (t, J = 7.1 Hz, 3H). **^{13}C NMR (101 MHz, $CDCl_3$) δ**: 175.3, 144.7, 134.1, 129.8, 129.6, 129.2, 128.7, 127.7, 126.4, 108.0, 104.2, 76.8, 60.4, 32.7, 21.8, 21.7, 20.9, 14.4. **HRMS** (APCI): m/z calculated for $C_{22}H_{25}O_5S$ $[M+H]^+$: 401.1417, found 401.1430. **CCDC**: 2172800.

(R)-ethyl 2-butyl-3-((2S,3R)-3-(tosyloxy)butan-2-yl)cycloprop-2-enecarboxylate (1a-9)

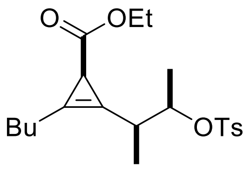

Prepared according to procedure C. **Scale, physical description, yield, mass:** 1.2 mmol, light yellow oil, 37% yield, 171 mg. **Flash chromatography:** 0 to 15 % EtOAc / petroleum ether. **^{1}H NMR (400 MHz, $CDCl_3$) δ:** 7.78 (d, J = 8.3 Hz, 2H), 7.33 (d, J = 8.0 Hz, 2H), 4.71 (qd, J = 6.4, 4.0 Hz, 1H), 4.07 (q, J = 7.1 Hz, 2H), 3.08 – 2.78 (m, 1H), 2.44 (s, 3H), 2.38 (td, J = 7.4, 1.2 Hz, 2H), 1.97 (s, 1H), 1.54 – 1.41 (m, 2H), 1.39 – 1.28 (m, 2H), 1.26 (d, J = 6.4 Hz, 3H), 1.21 (t, J = 7.1 Hz, 3H), 1.09 (d, J = 7.0 Hz, 3H), 0.89 (t, J = 7.3 Hz, 3H). **^{13}C NMR (101 MHz, $CDCl_3$) δ**: 176.5, 144.6, 134.4, 129.8, 127.7, 108.8, 105.2, 80.5, 60.0, 36.1, 28.9, 24.2, 22.4, 21.8, 21.7, 17.5, 14.4, 13.8 13.2. **HRMS** (APCI): m/z calculated for $C_{21}H_{31}O_5S$ $[M+H]^+$: 395.2071, found 395.1913.

(R)-ethyl 2-butyl-1-methyl-3-((R)-2-(tosyloxy)propyl)cycloprop-2-enecarboxylate (1a-10)

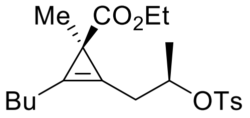

Prepared according to procedure C. **Scale, physical description, yield, mass:** 1.9 mmol, light yellow oil, 33% yield, 250 mg. **Flash chromatography:** 0 to 15 % EtOAc / petroleum ether. **^{1}H NMR (400 MHz, $CDCl_3$) δ:** 7.78 (d, J = 8.3 Hz, 2H), 7.33 (d, J = 7.7 Hz, 2H), 4.76 (td, J = 6.4, 5.4 Hz, 1H), 4.03 (q, J = 7.1 Hz, 2H), 2.67 (dt, J = 5.2, 1.4 Hz, 2H), 2.44 (s, 3H), 2.36 (tt, J = 7.4, 1.3 Hz, 2H), 1.51 – 1.41 (m, 2H), 1.40 – 1.30 (m, 2H), 1.31 (d, J = 6.3 Hz, 3H), 1.24 (s, 3H), 1.17 (t, J = 7.1 Hz, 3H), 0.89 (t, J = 7.3 Hz, 3H). **^{13}C NMR (101 MHz, $CDCl_3$) δ**: 177.4, 144.7, 134.4, 129.8, 127.7, 115.3, 106.0, 77.1, 60.1, 31.3, 29.0, 26.0, 23.7, 22.5, 21.7, 20.4, 19.1, 14.4, 13.8. **HRMS** (APCI): m/z calculated for $C_{21}H_{31}O_5S$ $[M+H]^+$: 395.1892, found 395.1880.

7. Protocols for the synthesis of chloro-cyclopropenes **6**

Procedure D: S_N2 displacement.

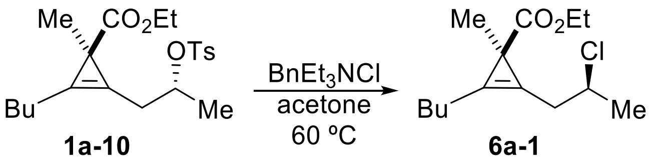

In a round bottom flask, open to air, cyclopropene **1a-10** (1 equiv.) was dissolved in acetone (0.5 M). $BnEt_3NCl$ (2 equiv.) was added at room temperature. The reaction was warmed and stirred at 60 °C and followed by TLC analysis of hydrolyzed aliquots (EtOAc/petroleum ether 20/80, $KMnO_4$ or *p*-anisaldehyde). Upon completion, the reaction mixture was poured into a solution of Et_2O/ 1 M HCl (2/1) mixture. The aqueous phase was extracted with Et_2O. The combined organic phases were washed with brine, dried over Na_2SO_4 and concentrated under reduced pressure. The crude reaction mixture was purified through column chromatography to give pure cyclopropene **6a-1**.

Procedure E: $SOCl_2$ chlorination of secondary alcohol.

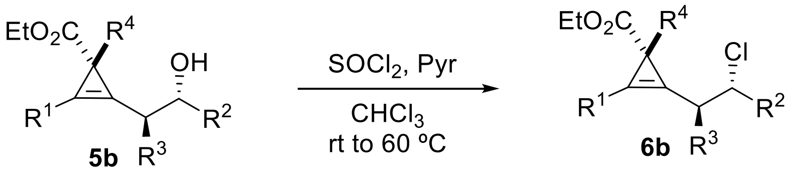

Under argon atmosphere, cyclopropene **5b** (1 equiv.) was dissolved in chloroform (0.5 M). Pyridine (2 equiv.) and thionyl chloride (2 equiv.) were added at 0 °C. The reaction was stirred at 60 °C and followed by TLC analysis of hydrolyzed aliquots (EtOAc/petroleum ether 20/80, $KMnO_4$ or *p*-anisaldehyde). Upon completion, the reaction mixture was poured into a solution of Et_2O/ 1 M HCl (2/1) mixture. The aqueous phase was extracted with Et_2O. The combined organic phase were washed with brine, dried over Na_2SO_4 and concentrated under reduced pressure. The crude mixture was purified through column chromatography to give pure cyclopropene **6b**.

8. Characterization of chlorinated chloro-cyclopropenes **6**

(R)-ethyl 2-butyl-3-((S)-2-chloropropyl)-1-methylcycloprop-2-enecarboxylate (6a-1)

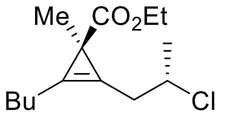

Prepared according to procedure D. **Scale, physical description, yield, mass:** 0.25 mmol, colorless oil, 54 % yield, 35 mg. **Flash chromatography:** 0 to 5 % EtOAc / petroleum ether. **^{1}H NMR (400 MHz, $CDCl_3$) δ:** 4.17 (h, J = 6.5 Hz, 1H), 4.04 (q, J = 7.1 Hz, 2H), 2.80 (ddt, J = 6.2, 2.9, 1.5 Hz, 2H), 2.40 (tt, J = 7.5, 1.5 Hz, 2H), 1.54 (d, J = 6.6 Hz, 3H), 1.52 – 1.42 (m, 2H), 1.41 – 1.32 (m, 2H), 1.30 (s, 3H), 1.18 (t, J = 7.1 Hz, 3H), 0.89 (t, J = 7.3 Hz, 3H). **^{13}C NMR (101 MHz, $CDCl_3$) δ**: 176.5, 113.5, 106.8, 59.2, 54.0, 34.1, 28.1, 25.4, 23.9, 22.7, 21.4, 18.2, 13.4, 12.7. **HRMS** (APCI): m/z calculated for $C_{14}H_{24}ClO_2$ $[M+H]^+$: 259.1459, found 259.1460.

(S)-ethyl 2-butyl-3-((R)-2-chloropropyl)cycloprop-2-enecarboxylate (6b-1)

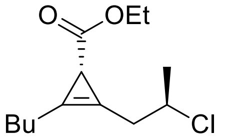

Prepared according to procedure E. **Scale, physical description, yield, mass:** 4.4 mmol, brown oil, 45% yield, 492 mg. **Flash chromatography:** 0 to 5 % EtOAc / petroleum ether. **^{1}H NMR (400 MHz, $CDCl_3$) δ:** 4.19 (h, J = 6.5 Hz, 1H), 4.11 (q, J = 7.1 Hz, 2H), 2.94 – 2.77 (m, 2H), 2.45 (t, J = 7.3 Hz, 2H), 2.11 (s, 1H), 1.58 (d, J = 6.6 Hz, 3H), 1.64 – 1.45 (m, 2H), 1.43 – 1.30 (m, 2H), 1.24 (t, J = 7.1 Hz, 3H), 0.91 (t, J = 7.3 Hz, 3H). **^{13}C NMR (101 MHz, $CDCl_3$) δ**: 176.6, 109.0, 102.5, 60.0, 54.9, 35.6, 28.9, 25.0, 24.3, 22.4, 22.2, 14.4, 13.8. **HRMS** (APCI): m/z calculated for $C_{13}H_{22}ClO_2$ $[M+H]^+$: 245.1303, found 245.1319.

9. Protocols for the synthesis of cyclopropyl methanols **7**

Procedure F: DIBAL reduction of the esters **6b-1** and **1a-1**.

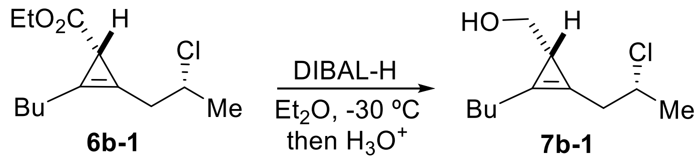

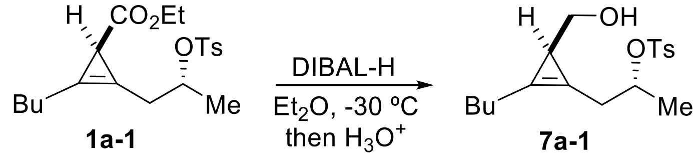

In a flame dried three necked flask equipped with an addition funnel, cyclopropenyl esters (1 equiv.) were dissolved in Et_2O (1 M) and cooled to -30 °C. DIBAL-H (2.2 equiv., 1 M in hexane) was added dropwise from the addition funnel. The reaction mixture was stirred at -30 °C until full consumption of the cyclopropenyl esters (followed by TLC, 15% EtOAc/ petroleum ether, p-anisaldehyde/$KMnO_4$, ca. 1 h). DIBAL-H like most aluminum compound reacts violently with air and water. Therefore, a careful hydrolysis should be performed at low temperature. At -30 °C, the reaction was therefore diluted with a solution of Et_2O mixed with an aqueous solution of 1 M HCl (40%). The mixture was stirred until two separated phases appeared. The aqueous layer was extracted three times with Et_2O, and the combined organic phases were dried over Na_2SO_4, filtered, and concentrated under reduced pressure. Crude mixture was then purified by flash chromatography using EtOAc / petroleum ether as eluent.

10. Characterization of cyclopropyl methanols **7**

((S)-2-butyl-3-((R)-2-chloropropyl)cycloprop-2-en-1-yl)methanol (7b-1)

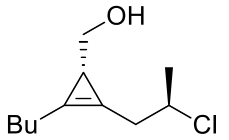

Prepared according to procedure F. **Scale, physical description, yield, mass:** 2 mmol, yellow oil, 83% yield, 339 mg. **Flash chromatography:** 0 to 15 % EtOAc / petroleum ether. **^{1}H NMR (400 MHz, $CDCl_3$) δ:** 4.23 (h, J = 6.5 Hz, 1H), 3.60 (dd, J = 10.7, 4.1 Hz, 1H), 3.50 (dd, J = 10.7, 4.7 Hz, 1H), 2.86 (d, J = 6.4 Hz, 2H), 2.44 (t, J = 7.4 Hz, 2H), 1.66 (t, J = 4.4 Hz, 1H), 1.57 (d, J = 6.6 Hz, 3H), 1.55 – 1.48 (m, 2H), 1.43 – 1.29 (m, 2H), 0.92 (t, J = 7.3 Hz, 3H). **^{13}C NMR (101 MHz, $CDCl_3$) δ**: 117.5, 110.6, 68.6, 56.0, 36.6, 29.6, 25.4, 25.1, 22.8, 22.5, 13.9. **HRMS** (APCI): m/z calculated for $C_{11}H_{18}ClO$ $[M-H]^+$: 201.1041, found 201.1036.

(R)-1-((R)-2-butyl-3-(hydroxymethyl)cycloprop-1-en-1-yl)propan-2-yl 4-methylbenzenesulfonate (7a-1)

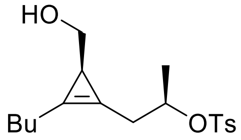

Prepared according to procedure F. **Scale, physical description, yield, mass:** 5 mmol, colorless oil, 75% yield, 1.31 g. **Flash chromatography:** 0 to 15 % EtOAc / petroleum ether. **^{1}H NMR (400 MHz, $CDCl_3$) δ:** 7.78 (d, J = 8.3 Hz, 2H), 7.36 – 7.30 (m, 2H), 4.85 (h, J = 6.1 Hz, 1H), 3.55 (dd, J = 10.7, 4.1 Hz, 1H), 3.40 (ddd, J = 10.8, 4.9, 0.7 Hz, 1H), 2.73 (d, J = 5.7 Hz, 2H), 2.44 (s, 3H), 2.38 (t, J = 7.4 Hz, 2H), 1.53 (t, J = 4.5 Hz, 1H), 1.48 (dd, J = 10.3, 4.8 Hz, 2H), 1.39 – 1.23 (m, 2H), 1.30 (d, J = 6.3 Hz, 3H), 0.90 (t, J = 7.3 Hz, 3H). **^{13}C NMR (101 MHz, $CDCl_3$) δ**: 144.7, 134.4, 129.8, 127.7, 118.1, 108.9, 77.9, 77.3, 68.5, 32.8, 29.5, 25.3, 22.53, 22.52, 21.7, 20.6, 13.8. **HRMS** (APCI): m/z calculated for $C_{18}H_{25}O_4S$ $[M+H]^+$: 337.1747, found 337.1502.

11. Protocols for the synthesis of spiropentanes **3, 8**

Procedure G: Carbometalation – intramolecular nucleophilic substitution sequence.

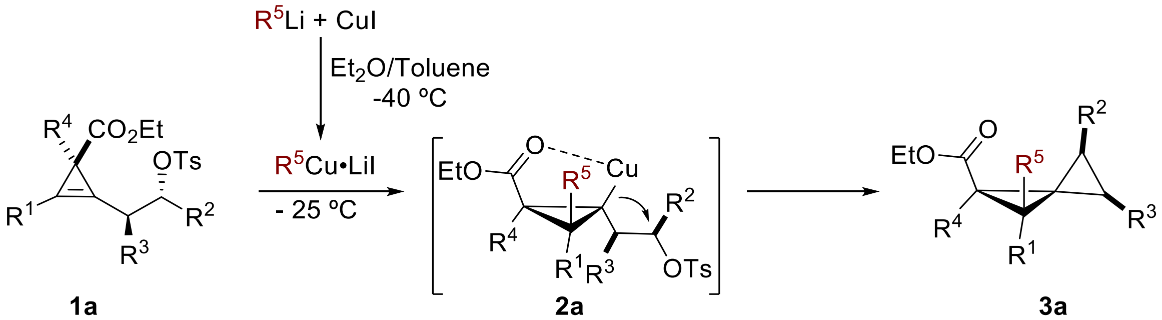

In a flame-dried 3-neck flask equiped with a thermometer, CuI (3 equiv.) was added to a 1:1 mixture of Et_2O:toluene (15 mL:15 mL). This mixture was cooled down to -40 °C and organolithium reagent (3 equiv.) was added dropwise maintaining the temperature between -40 °C to -30 °C. The mixture was stirred for 1 hour at the same temperature. The formation of the reactive organocopper reagent was indicated by the formation of a clear brown solution that might evolve with time into a more greenish solution. Cyclopropene **1** (1 equiv., 0.5 mmol) in 5 mL of Et_2O or 5 mL of toluene was added and the reaction mixture was stirred at -25 °C. The reaction was followed by TLC analysis of hydrolyzed aliquots (EtOAc/petroleum ether 20/80, $KMnO_4$ or *p*-anisaldehyde) and upon completion, was quenched with 20 mL of a 1:1 mixture of aqueous saturated solution of NH_4Cl and aqueous NH_4OH (25%). The aqueous phase was extracted with Et_2O. The combined organic phases were washed with brine, dried over Na_2SO_4 and concentrated under reduced pressure. The crude mixture was purified through column chromatography to give the pure spiropentanes **3, 8**.

12. Characterization of spiropentanes **3, 8**

(1R,2S,3R,4S)-ethyl 2-butyl-2-hexyl-4-methylspiro[2.2]pentane-1-carboxylate (3a-1)

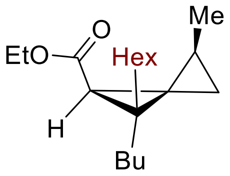

Prepared according to procedure G. **Scale, physical description, yield, mass:** 0.5 mmol, colorless oil, 95% yield, 140 mg. **Flash chromatography:** 0 to 3 % EtOAc / petroleum ether. **^{1}H NMR (400 MHz, $CDCl_3$) δ:** 4.09 – 4.01 (m, 1H), 4.01 – 3.89 (m, 1H), 1.79 (ddd, J = 13.0, 11.6, 4.5 Hz, 1H), 1.70 – 1.50 (m, 2H), 1.49 (s, 1H), 1.35 – 1.14 (m, 12H), 1.17 (t, J = 7.2 Hz, 3H), 1.07 (d, J = 6.1 Hz, 3H), 1.11- 1.03 (m, J = 4.4 Hz, 2H), 0.83 (t, J = 7.1 Hz, 3H), 0.80 (t, J = 6.9 Hz, 3H), 0.75 (dd, J = 8.0, 4.3 Hz, 1H), 0.41 (t, J = 4.7 Hz, 1H). **^{13}C NMR (101 MHz, $CDCl_3$) δ:** 171.4, 58.7, 35.0, 34.9, 32.3, 30.9, 30.4, 28.7, 27.3, 25.9, 25.7, 22.1, 21.6, 15.8, 13.4, 13.1, 13.0, 12.2, 11.3. **HRMS** (APCI): m/z calculated for $C_{19}H_{35}O_2$ $[M+H]^+$: 295.2632, found 295.2666.

(1R,2R,3R,4S)-ethyl 2-butyl-2-ethyl-4-methylspiro[2.2]pentane-1-carboxylate (3a-2)

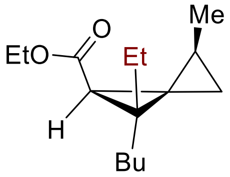

Prepared according to procedure G. **Scale, physical description, yield, mass:** 0.33 mmol, light yellow oil, 84% yield, 66 mg. **Flash chromatography:** 0 to 3 % EtOAc / petroleum ether. **^{1}H NMR (400 MHz, $CDCl_3$) δ:** 4.17 – 4.00 (m, 2H), 1.92 – 1.82 (m, 1H), 1.80 – 1.61 (m, 2H), 1.56 (s, 1H), 1.42 – 1.28 (m, 3H), 1.29 – 1.21 (m, 4H), 1.22 – 1.16 (m, 1H), 1.14 (t, J = 4.9 Hz, 4H), 0.89 (t, J = 7.1 Hz, 3H), 0.87 – 0.78 (m, 4H), 0.49 (t, J = 4.7 Hz, 1H). **^{13}C NMR (101 MHz, $CDCl_3$) δ:** 171.5, 58.8, 35.8, 34.2, 32.6, 30.1, 27.24, 22.11, 18.8, 15.8, 13.4, 13.1, 12.1, 11.4, 9.7. **HRMS** (APCI): m/z calculated for $C_{15}H_{27}O_2$ $[M+H]^+$: 239.2006, found 239.2013.

(1R,2S,3R,4S)-ethyl 2-butyl-2-ethyl-4-methylspiro[2.2]pentane-1-carboxylate (3a-3)

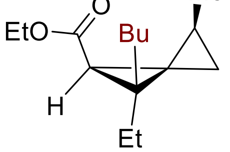

Prepared according to procedure G. **Scale, physical description, yield, mass:** 0.5 mmol, light yellow oil, 83% yield, 99 mg. **Flash chromatography:** 0 to 3 % EtOAc / petroleum ether. **^{1}H NMR (400 MHz, $CDCl_3$) δ:** 4.18 – 3.99 (m, 2H), 1.89 (dq, J = 14.6, 7.4 Hz, 1H), 1.67 (tdt, J = 14.3, 10.6, 6.9 Hz, 2H), 1.57

(s, 1H), 1.42 – 1.33 (m, 2H), 1.24 (t, J = 7.1 Hz, 3H), 1.19-1.30 (m, 3H), 1.14 (d, J = 6.1 Hz, 3H), 1.11 – 1.00 (m, 1H), 0.88 (q, J = 7.3 Hz, 6H), 0.83 (dd, J = 8.0, 4.3 Hz, 1H), 0.51 (t, J = 4.7 Hz, 1H). **^{13}C NMR (101 MHz, CDCl$_3$) δ:** 171.5, 58.8, 35.6, 32.2, 30.2, , 25.0, 22.0, 15.8, 13.3, 13.1, 12.0, 11.1, 9.3. **HRMS** (APCI): m/z calculated for $C_{15}H_{27}O_2$ [M+H]$^+$: 239.2006, found 239.2019.

(1R,2S,3R,4S)-ethyl 2-butyl-2-isopropyl-4-methylspiro[2.2]pentane-1-carboxylate (3a-4)

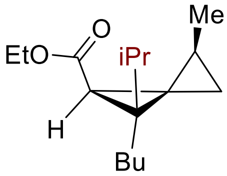

Note: compound **(3a-4)** *is obtained as a 1:3 mixture of diastereomers*

Prepared according to procedure G. **Scale, physical description, yield, mass:** 0.5 mmol, colorless oil, 74% yield, 93 mg. **Flash chromatography:** 0 to 3 % EtOAc / petroleum ether. **^{1}H NMR (400 MHz, CDCl$_3$) δ:** 4.12 – 3.94 (m, 3H), 2.41 – 2.28 (m, 0.4H), 2.24 – 2.06 (m, 0.3H), 1.96 (d, J = 11.0 Hz, 0.3H), 1.76 – 1.45 (m, 6H), 1.35 – 1.00 (m, 13H), 0.96 (d, J = 5.9 Hz, 3H), 0.92 (ddd, J = 8.0, 4.9, 0.8 Hz, 1H), 0.89 (d, J = 7.0 Hz, 1H), 0.87 – 0.73 (m, 13H), 0.45 (t, J = 4.9 Hz, 1H), 0.39 (t, J = 4.8 Hz, 0.35H). **^{13}C NMR (101 MHz, CDCl$_3$) δ**: major: 72.9, 59.8, 39.6, 33.8, 32.6, 29.4, 27.9, 25.7, 23.4, 19.6, 19.3, 16.9, 14.4, 14.2, 11.9, 11.1. minor: 172.9, 59.9, 40.6, 33.0, 31.5, 30.2, 29.1, 28.2, 23.6, 20.8, 20.6, 17.3, 14.1, 13.8, 13.6. **HRMS** (APCI): m/z calculated for $C_{16}H_{29}O_2$ [M+H]$^+$: 253.2162, found 253.2168.

(1R,2R,3R,4S)-ethyl 2-ethyl-4-methyl-2-phenethylspiro[2.2]pentane-1-carboxylate (3a-5)

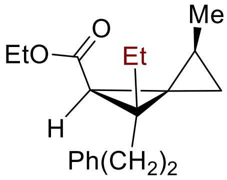

Prepared according to procedure G. **Scale, physical description, yield, mass:** 0.12 mmol, colorless oil, 79% yield, 28 mg. **Flash chromatography:** 0 to 3 % EtOAc / petroleum ether. **^{1}H NMR (400 MHz, CDCl$_3$) δ:** 7.33 – 7.24 (m, 2H), 7.19 (tq, J = 4.6, 1.6 Hz, 3H), 4.20 – 4.03 (m, 2H), 2.72 (ddd, J = 13.5, 11.8, 5.2 Hz, 1H), 2.53 (ddd, J = 13.4, 11.7, 5.1 Hz, 1H), 2.19 (ddd, J = 13.8, 11.8, 5.2 Hz, 1H), 1.83 (tq, J = 14.6, 7.3 Hz, 2H), 1.51 (ddd, J = 13.8, 11.8, 5.1 Hz, 1H), 1.44 (s, 1H), 1.42 – 1.37 (m, 1H), 1.26 (t, J = 7.1 Hz, 3H), 1.16 (d, J = 6.1 Hz, 3H), 0.93 (t, J = 7.4 Hz, 3H), 0.87 (dd, J = 8.0, 4.4 Hz, 1H), 0.49 (t, J = 4.7 Hz, 1H). **^{13}C NMR (101 MHz, CDCl$_3$) δ**: 171.3, 141.2, 127.4, 127.3, 124.8, 58.8, 36.7, 35.4, 32.6, 31.5, 30.1, 29.3, 18.8, 15.8, 13.3, 12.12 11.4, 9.8. **HRMS** (APCI): m/z calculated for $C_{19}H_{27}O_2$ [M+H]$^+$: 287.2006, found 287.2033.

(1R,2R,3R,4S)-ethyl 2-butyl-2-(2-((tert-butyldimethylsilyl)oxy)ethyl)-4-methylspiro[2.2]pentane-1-carboxylate (3a-6)

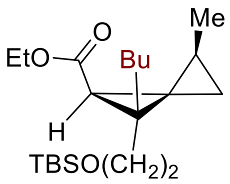

Prepared according to procedure G. **Scale, physical description, yield, mass:** 0.64 mmol, colorless oil, 76% yield, 181 mg. **Flash chromatography:** 0 to 3 % EtOAc / petroleum ether. **^{1}H NMR (400 MHz, $CDCl_3$) δ:** 4.18 – 3.99 (m, 2H), 3.74 – 3.58 (m, 2H), 2.10 (ddd, J = 13.6, 7.8, 5.6 Hz, 1H), 1.75 – 1.57 (m, 3H), 1.51 – 1.33 (m, 3H), 1.30 – 1.20 (m, 5H), 1.15 (d, J = 6.1 Hz, 3H), 1.11 – 1.04 (m, 1H), 0.95 – 0.81 (m, 13H), 0.54 (t, J = 4.7 Hz, 1H), 0.05 (d, J = 1.2 Hz, 6H). **^{13}C NMR (101 MHz, $CDCl_3$) δ**: 172.3, 60.8, 59.9, 38.9, 34.0, 33.3, 31.1, 29.2, 27.0, 26.5, 23.0, 18.4, 16.8, 14.4, 14.2, 13.5, 12.9, 1.1, -5.24, -5.27. **HRMS** (APCI): m/z calculated for $C_{21}H_{41}O_3Si$ $[M+H]^+$: 369.2819, found 369.2793.

(1S,2S,3R,4S)-ethyl 2-ethyl-4-methyl-2-phenylspiro[2.2]pentane-1-carboxylate (3a-7)

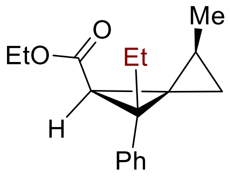

Prepared according to procedure G. **Scale, physical description, yield, mass:** 1.5 mmol, colorless oil, 82% yield, 320 mg. **Flash chromatography:** 0 to 3 % EtOAc / petroleum ether. **^{1}H NMR (400 MHz, $CDCl_3$) δ:** 7.38 – 7.28 (m, 2H), 7.27 – 7.20 (m, 3H), 4.28 – 4.11 (m, 2H), 2.29 (s, 1H), 2.19 (dq, J = 14.8, 7.4 Hz, 1H), 1.89 (dq, J = 14.4, 7.3 Hz, 1H), 1.55 (dq, J = 8.1, 5.8 Hz, 1H), 1.34 (d, J = 5.2 Hz, 3H), 1.32 (t, J = 6.6 Hz, 3H), 0.86 (dd, J = 8.1, 4.8 Hz, 1H), 0.76 (t, J = 7.3 Hz, 3H), 0.53 (t, J = 5.0 Hz, 1H). **^{13}C NMR (101 MHz, $CDCl_3$) δ**: 171.1, 142.1, 127.8, 127.1, 125.3, 59.1, 40.4, 32.2, 29.4, 23.9, 16.0, 13.4, 12.6, 12.4, 10.2. **HRMS** (APCI): m/z calculated for $C_{17}H_{23}O_2$ $[M+H]^+$: 259.1698, found 259.1717.

(1R,2R,3R,4S)-ethyl 2-butyl-2,4-diethylspiro[2.2]pentane-1-carboxylate (3a-8)

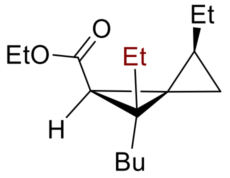

Prepared according to procedure G. **Scale, physical description, yield, mass:** 0.5 mmol, colorless oil, 95% yield, 120 mg. **Flash chromatography:** 0 to 3 % EtOAc / petroleum ether. **^{1}H NMR (400 MHz, $CDCl_3$) δ:** 4.18 – 4.01 (m, 2H), 1.90 – 1.81 (m, 1H), 1.80 – 1.61 (m, 3H), 1.55 (s, 1H), 1.36 – 1.21 (m, 7H), 1.20 – 1.09 (m, 2H), 1.05 – 0.93 (m, 4H), 0.90 (t, J = 7.2 Hz, 3H), 0.86 – 0.77 (m, 4H), 0.49 (t, J = 4.7 Hz, 1H). **^{13}C NMR (101 MHz, $CDCl_3$) δ**: 171.5, 58.7, 35.7, 34.3, 32.5, 30.2, 27.2, 24.2, 22.1, 19.7, 19.0, 13.4, 13.1, 12.8, 10.0, 9.7. **HRMS** (APCI): m/z calculated for $C_{16}H_{29}O_2$ $[M+H]^+$: 253.2162, found 253.2171.

(1R,2R,3R,4S)-ethyl 2-butyl-4-(methoxymethyl)-2-methylspiro[2.2]pentane-1-carboxylate (3a-9)

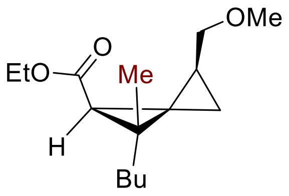

Prepared according to procedure G. **Scale, physical description, yield, mass:** 0.5 mmol, colorless oil, 76% yield, 97 mg. **Flash chromatography:** 0 to 3 % EtOAc / petroleum ether. **^{1}H NMR (400 MHz, $CDCl_3$) δ:** 4.10 (q, J = 7.1 Hz, 2H), 3.75 (dd, J = 10.1, 4.8 Hz, 1H), 3.37 (s, 3H), 3.01 (dd, J = 10.1, 9.0 Hz, 1H), 1.75 – 1.68 (m, 1H), 1.65 (s, 1H), 1.45 – 1.28 (m, 6H), 1.25 (t, J = 7.1 Hz, 3H), 1.17 (s, 3H), 0.99 (dd, J = 7.8, 4.6 Hz, 1H), 0.90 (t, J = 7.1 Hz, 3H), 0.78 (t, J = 4.8 Hz, 1H). **^{13}C NMR (101 MHz, $CDCl_3$) δ:** 172.0, 74.7, 60.0, 58.4, 39.4, 31.1, 31.0, 30.9, 28.6, 23.0, 18.1, 14.7, 14.4, 14.1, 10.6. **HRMS** (APCI): m/z calculated for $C_{15}H_{27}O_3$ $[M+H]^+$: 255.1960, found 255.1966.

(1R,2R,3s,4R,5S)-ethyl 2-butyl-2-ethyl-4,5-dimethylspiro[2.2]pentane-1-carboxylate (3a-10)

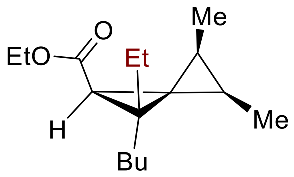

Prepared according to procedure G. **Scale, physical description, yield, mass:** 0.42 mmol, colorless oil, 95% yield, 100 mg. **Flash chromatography:** 0 to 3 % EtOAc / petroleum ether. **^{1}H NMR (400 MHz, $CDCl_3$) δ:** 4.16 – 4.00 (m, 2H), 1.72 (qd, J = 7.3, 3.1 Hz, 2H), 1.56 (s, 1H), 1.50 – 1.46 (m, 1H), 1.35 – 1.27 (m, 4H), 1.24 (t, J = 7.1 Hz, 3H), 1.21 – 1.15 (m, 1H), 1.09 (d, J = 6.4 Hz, 3H), 1.03 (d, J = 6.3 Hz, 3H), 0.91 (t, J = 7.0 Hz, 3H), 0.86 (t, J = 7.4 Hz, 3H). **^{13}C NMR (101 MHz, $CDCl_3$) δ**: 171.6, 58.7, 36.8, 36.3, 33.5, 30.2, 27.7, 22.2, 19.4, 15.7, 14.9, 13.4, 13.1, 9.8, 9.6, 9.5. **HRMS** (APCI): m/z calculated for $C_{16}H_{29}O_2$ $[M+H]^+$: 253.2168, found 253.2178.

(1R,2S,3S,4S)-ethyl 2-butyl-2-ethyl-1,4-dimethylspiro[2.2]pentane-1-carboxylate (3a-11)

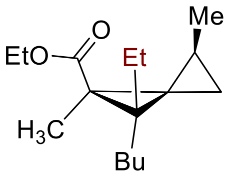

Note: compound **(3a-11)** *was isolated as a 86:14 mixture of diastereomers.*

Prepared according to procedure G. **Scale, physical description, yield, mass:** 0.32 mmol, colorless oil, 70% yield, 58 mg. **Flash chromatography:** 0 to 3 % EtOAc / petroleum ether. **^{1}H NMR (400 MHz, $CDCl_3$) δ:** δ 4.08 (t, J = 7.2 Hz, 2H), 1.75 – 1.68 (m, 1H), 1.67 – 1.60 (m, 2H), 1.51 – 1.45 (m, 1H), 1.31 – 1.28 (m, 3H), 1.26 – 1.21 (m, 6H), 1.20 – 1.15 (m, 4H), 1.12 (d, J = 6.1 Hz, 3H), 0.90 (d, J = 7.3 Hz, 4H), 0.87 – 0.83 (m, 2H), 0.80 (t, J = 7.4 Hz, 3H), 0.63 (dd, J = 8.0, 4.3 Hz, 1H), 0.60 – 0.56 (m, 0.15H), 0.33 (dd, J = 6.5, 5.1 Hz, 0.17H), 0.26 (t, J = 4.6 Hz, 1H). **^{13}C NMR (101 MHz, $CDCl_3$) δ**: 173.8, 58.9, 35.34 35.3, 29.6,

28.7, 27.3, 22.4, 20.0, 16.0, 14.4, 13.8, 13.3, 13.1, 13.1, 9.6, 9.2. **HRMS** (APCI): m/z calculated for $C_{16}H_{29}O_2$ $[M+H]^+$: 253.2162, found 253.2170.

(1R,2S,3S,4R)-ethyl 2-butyl-2-ethyl-1,4-dimethylspiro[2.2]pentane-1-carboxylate (3a-12)

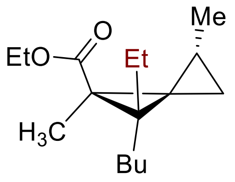

Prepared according to procedure G. **Scale, physical description, yield, mass:** 0.13 mmol, colorless oil, 82% yield, 28 mg. **Flash chromatography:** 0 to 3 % EtOAc / petroleum ether. **^{1}H NMR (400 MHz, $CDCl_3$) δ:** 4.10 (q, J = 7.1 Hz, 2H), 1.66 (dt, J = 14.9, 7.4 Hz, 1H), 1.59 – 1.35 (m, 4H), 1.35 – 1.28 (m, 5H), 1.28 – 1.20 (m, 5H), 1.11 (d, J = 6.1 Hz, 3H), 0.89 (t, J = 7.0 Hz, 3H), 0.78 (t, J = 7.5 Hz, 3H), 0.64 (dd, J = 8.0, 4.1 Hz, 1H), 0.11 (t, J = 4.6 Hz, 1H). **^{13}C NMR (101 MHz, $CDCl_3$) δ**: 173.2, 58.8, 36.4, 34.0, 29.7, 28.5, 27.2, 22.2, 20.5, 17.5, 15.1, 13.3, 13.1, 9.5, 8.5. **HRMS** (APCI): m/z calculated for $C_{16}H_{29}O_2$ $[M+H]^+$: 253.2162, found 253.2176.

(1S,2S,3S,4S)-ethyl 2-butyl-2-ethyl-4-methylspiro[2.2]pentane-1-carboxylate (3b-1)

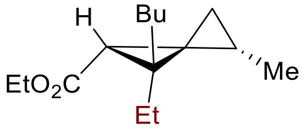

Note: compound **(3b-1)** *was isolated as a 90:10 mixture of diastereomers.*

Prepared according to procedure G. **Scale, physical description, yield, mass:** 0.46 mmol, light yellow oil, 90% yield, 82 mg. **Flash chromatography:** 0 to 3 % EtOAc / petroleum ether. **^{1}H NMR (400 MHz, $CDCl_3$) δ:** 4.08 (qq, J = 6.6, 3.7 Hz, 2H), 1.76 – 1.65 (m, 2H), 1.62 (s, 1H), 1.45 (dt, J = 14.2, 9.4 Hz, 1H), 1.31 – 1.19 (m, 9H), 1.07 (d, J = 6.0 Hz, 3H), 0.88 (d, J = 6.9 Hz, 3H), 0.85 – 0.75 (m, 4H), 0.53 (t, J = 4.9 Hz, 0.1H), 0.29 (t, J = 4.6 Hz, 1H). **^{13}C NMR (101 MHz, $CDCl_3$) δ**: 171.4, 58.7, 33.1, 33.8, 33.6, 29.2, 27.2, 22.0, 19.4, 16.3, 13.3, 13.1, 10.7, 10.2, 9.4. **HRMS** (APCI): m/z calculated for $C_{15}H_{27}O$ $[M+H]^+$: 239.2006, found 239.2015.

(1S,2S,3S,4S)-ethyl 2-butyl-2-(2-((tert-butyldimethylsilyl)oxy)ethyl)-4-methylspiro[2.2]pentane-1-carboxylate (3b-2)

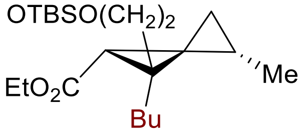

Note: compound **(3b-2)** *was synthesized as a 90:10 mixture of diastereomers*

Prepared according to procedure G. **Scale, physical description, yield, mass:** 0.41 mmol, colorless oil, 69% yield, 106 mg. **Flash chromatography:** 0 to 3 % EtOAc / petroleum ether. **^{1}H NMR (400 MHz, $CDCl_3$) δ:** 7 4.13 – 4.01 (m, 2H), 3.63 (td, J = 7.5, 4.5 Hz, 2H), 1.76 – 1.52 (m, 5H), 1.33 – 1.15 (m, 8H), 1.07 (d, J = 6.1 Hz, 3H), 0.94 – 0.79 (m, 18H), 0.30 (t, J = 4.6 Hz, 1H), 0.03 (s, 6H). **^{13}C NMR (101 MHz, $CDCl_3$) δ**: 170.9, 59.7, 58.8, 37.5, 33.5, 31.0, 28.9, 27.6, 26.4, 24.9, 22.0, 17.3, 16.2, 13.3, 13.1, 11.0, 10.7, -6.39, -6.41. **HRMS** (APCI): m/z calculated for $C_{21}H_{41}O_3Si$ [M+H]$^+$: 369.2819, found 369.2827.

((1S,2S,3S,4S)-2-butyl-2,4-dimethylspiro[2.2]pentan-1-yl)methanol (8b-1)

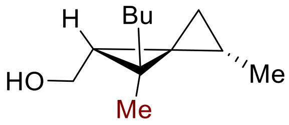

Prepared according to procedure G. **Scale, physical description, yield, mass:** 0.5 mmol, colorless oil, 81% yield, 74 mg. **Flash chromatography:** 0 to 15 % EtOAc / petroleum ether. **^{1}H NMR (400 MHz, $CDCl_3$) δ:** 3.65 (dd, J = 11.3, 6.5 Hz, 1H), 3.58 (dd, J = 11.4, 8.2 Hz, 1H), 1.82 (brs, 1H), 1.39 – 1.18 (m, 7H), 1.15 – 1.10 (m, 1H), 1.09 (s, 3H), 1.04 (d, J = 5.7 Hz, 3H), 0.95 (dd, J = 8.3, 6.5 Hz, 1H), 0.87 (t, J = 7.2 Hz, 3H), 0.67 (dd, J = 7.5, 4.0 Hz, 1H), 0.33 (t, J = 4.1 Hz, 1H). **^{13}C NMR (101 MHz, $CDCl_3$) δ**: 63.0, 39.7, 29.8, 29.3, 28.9, 24.1, 23.1, 17.1, 15.0, 14.2, 11.3, 10.9. **HRMS** (APCI): m/z calculated for $C_{12}H_{21}$ [M-OH]$^+$: 165.1643, found 165.1638.

((1R,2R,3R,4S)-2-butyl-2-ethyl-4-methylspiro[2.2]pentan-1-yl)methanol (8a-1)

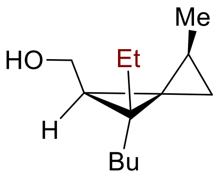

Prepared according to procedure G. **Scale, physical description, yield, mass:** 0.5 mmol, colorless oil, 68% yield, 67 mg. **Flash chromatography:** 0 to 15 % EtOAc / petroleum ether. **^{1}H NMR (400 MHz, $CDCl_3$) δ:** 3.71 (dd, J = 11.2, 7.5 Hz, 1H), 3.59 (dd, J = 11.2, 6.9 Hz, 1H), 1.68 – 1.54 (m, 2H), 1.37 (dt, J = 14.6, 7.5 Hz, 1H), 1.31 – 1.22 (m, 3H), 1.21 – 1.10 (m, 3H), 1.06 (d, J = 5.8 Hz, 3H), 0.93 (t, J = 7.2 Hz, 1H), 0.92 (t, J = 7.4 Hz, 3H), 0.88 (t, J = 7.1 Hz, 3H), 0.70 (dd, J = 7.6, 4.0 Hz, 1H), 0.38 (t, J = 4.2 Hz, 1H). **^{13}C NMR (101 MHz, $CDCl_3$) δ** 61.5, 34.4, 29.3, 28.7, 28.1, 27.2, 22.2, 20.7, 16.4, 13.1, 10.6, 10.4, 9.8. **HRMS** (APCI): m/z calculated for $C_{13}H_{25}O$ [M+H]$^+$: 197.1900, found 197.1895.

((1R,2S,3R,4S)-2-butyl-2-hexyl-4-methylspiro[2.2]pentan-1-yl)methanol (8a-2)

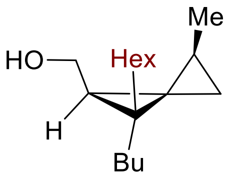

Prepared according to procedure G. **Scale, physical description, yield, mass:** 0.5 mmol, colorless oil, 75% yield, 95 mg. **Flash chromatography:** 0 to 15% EtOAc / petroleum ether. **^{1}H NMR (400 MHz, $CDCl_3$) δ:** 3.73 (dd, J = 11.2, 7.6 Hz, 1H), 3.61 (dd, J = 11.2, 6.9 Hz, 1H), 1.64 – 1.47 (m, 2H), 1.39 – 1.24 (m, 12H), 1.22 – 1.10 (m, 3H), 1.08 (t, J = 5.8 Hz, 3H), 0.95 (t, J = 7.2 Hz, 1H), 0.89 (t, J = 6.9 Hz, 3H), 0.88 (t, J = 6.9 Hz, 3H), 0.71 (dd, J = 7.5, 4.0 Hz, 1H), 0.40 (t, J = 4.2 Hz, 1H). **^{13}C NMR (101 MHz, $CDCl_3$) δ:** 62.8, 36.1, 31.9, 30.5, 30.2, 29.6, 29.2, 28.6, 28.3, 27.2, 23.3, 22.7, 17.4, 14.2, 14.1, 11.6, 10.9. **HRMS** (APCI): m/z calculated for $C_{17}H_{31}O$ $[M-H]^+$: 251.2375, found 251.2369.

((1R,2R,3R,4S)-2-butyl-2,4-dimethylspiro[2.2]pentan-1-yl)methanol (8a-3)

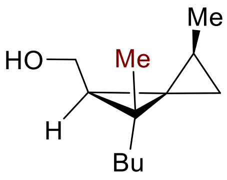

Prepared according to procedure G. **Scale, physical description, yield, mass:** 0.5 mmol, colorless oil, 76% yield, 69 mg. **Flash chromatography:** 0 to 15 % EtOAc / petroleum ether. **^{1}H NMR (400 MHz, $CDCl_3$) δ:** 3.70 (dd, J = 11.4, 6.4 Hz, 1H), 3.61 (dd, J = 11.3, 8.5 Hz, 1H), 1.44 – 1.23 (m, 7H), 1.17 – 1.14 (m, 1H), 1.13 (s, 3H), 1.07 (d, J = 5.7 Hz, 3H), 0.99 (dd, J = 8.5, 6.4 Hz, 1H), 0.90 (t, J = 7.1 Hz, 3H), 0.70 (dd, J = 7.4, 4.0 Hz, 1H), 0.36 (t, J = 4.1 Hz, 1H). **^{13}C NMR (101 MHz, $CDCl_3$) δ**: 63.2, 39.7, 30.0, 29.3, 28.9, 24.2, 23.1, 17.2, 15.1, 14.2, 11.3, 11.0. **HRMS** (APCI): m/z calculated for $C_{12}H_{23}O$ $[M+H]^+$: 183.1749, found 183.1731.

13. Miscellaneous synthetic protocols

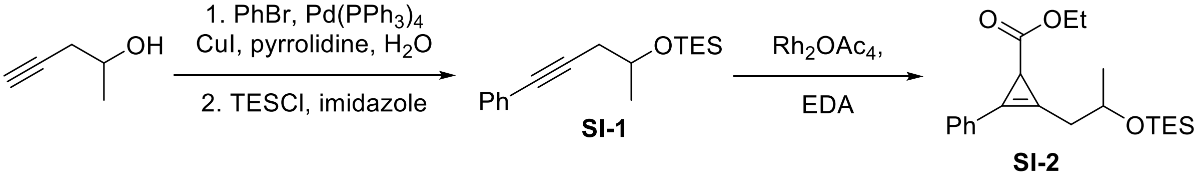

A round bottom flask open to air and fitted with a reflux condenser was charged with aryl halide (2 equiv.), terminal alkyne (1 equiv.), pyrrolidine (3 equiv.), $Pd(PPh_3)_4$ (0.5 mol %), CuI (1 mol %), and water (0.5 M). It was then immersed in an oil bath kept at 70 °C. After vigorous stirring for 30 min (the reaction darkened in color), it was cooled to room temperature and extracted with ether. The combined organic layers were dried and the solvent removed under reduced pressure. The crude reaction mixture was then dissolved in DCM and TESCl (1.2 equiv.) and imidazole (1.5 equiv.) were added. The mixture was stirred at 30 °C. Upon completion of the reactionby TLC analysis of hydrolyzed aliquots (EtOAc/petroleum ether 20/80, $KMnO_4$ or *p*-anisaldehyde), the reaction was diluted with

petroleum ether, filtered over celite and concentrated. Silica-gel chromatography of the residue produced internal alkyne **SI-1**.

A flame-dried round bottom flask was charged with internal alkyne **SI-1** (1.3 equiv.), Rh_2OAc_4 (0.5 mol%) and dry dichloromethane under an argon atmosphere. A solution of ethyl diazoacetate in dry dichloromethane (1.0 equiv.) was added over a period of 15 h via a syringe pump at ambient temperature. After further 2 h, the solvent was removed under reduced pressure and the reaction mixture was directly transferred to silica gel column chromatography to yield the pure product **SI-2** as a yellowish oil.

Triethyl((5-phenylpent-4-yn-2-yl)oxy)silane (SI-1)

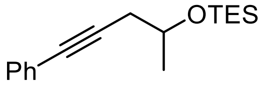

*Note: compound **SI-1** was isolated along with water (H_2O) as contaminant.*

Scale, physical description, yield, mass: 90 mmol, yellowish oil, 92% yield, 22.5 g. **Flash chromatography:** 0 to 2 % EtOAc / petroleum ether. **^{1}H NMR (400 MHz, CDCl$_3$) δ:** δ 7.38 (dd, J = 4.9, 1.7 Hz, 2H), 7.26 (dt, J = 5.7, 2.6 Hz, 3H), 4.03 (m, 1H), 2.58 (dd, J = 16.5, 5.6 Hz, 1H), 2.46 (dd, J = 16.5, 7.3 Hz, 1H), 1.30 (d, J = 6.0 Hz, 3H), 0.97 (t, J = 7.9 Hz, 9H), 0.62 (q, J = 7.7 Hz, 6H). **^{13}C NMR (101 MHz, CDCl$_3$) δ**: 131.6, 128.2, 127.6, 123.9, 87.6, 82.0, 67.7, 30.5, 23.6, 6.9, 4.9. **HRMS** (APCI): m/z calculated for $C_{17}H_{27}OS$ $[M+H]^+$: 275.1826, found 275.1843.

Ethyl 2-phenyl-3-(2-((triethylsilyl)oxy)propyl)cycloprop-2-enecarboxylate (**SI-2**)

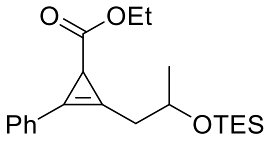

*Note: compound **SI-2** is obtained as a 1:1 mixture of diastereomers.*

*Compound **SI-2** was isolated with less than 10% (molar) of diethyl fumarate.*

*Compound **SI-2** was isolated along with water (H_2O) as contaminant.*

Scale, physical description, yield, mass: 60 mmol, yellowish oil, 24% yield, 5.31 g. **Flash chromatography:** 0 to 3 % EtOAc / petroleum ether. **^{1}H NMR (400 MHz, CDCl$_3$) δ:** 7.52 (ddt, J = 7.1, 5.9, 1.4 Hz, 2H), 7.43 – 7.36 (m, 2H), 7.35 – 7.28 (m, 1H), 4.24 – 4.05 (m, 3H), 3.01 – 2.70 (m, 2H), 2.44 (d, J = 2.2 Hz, 1H), 1.34 – 1.20 (m, 6H), 0.96 (td, J = 7.9, 5.2 Hz, 9H), 0.61 (qd, J = 7.7, 6.0 Hz, 6H). **^{13}C NMR (101 MHz, CDCl$_3$) δ**: 175.8, 175.7, 133.7, 129.5, 129.5, 128.7, 128.6, 128.6, 127.2, 127.1, 107.2, 107.1, 106.1, 105.9, 66.7, 66.6, 60.1, 35.9, 35.7, 23.8, 23.7, 22.1, 22.0, 14.4, 6.87, 6.86, 4.9. **HRMS** (APCI): m/z calculated for $C_{21}H_{33}O_3S$ $[M+H]^+$: 361.2199, found 361.2216.

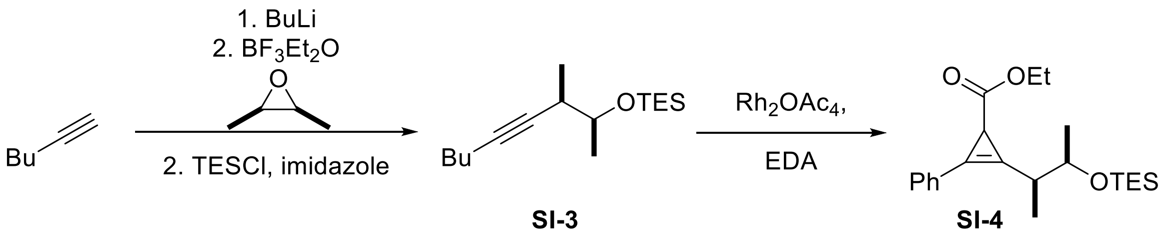

n-Butyllithium (1.5 equiv.) was added dropwise to a solution of 1-hexyne (1.5 equiv.) in THF (50 mL) at –78 °C and stirring was continued for 10 min. BF_3•Et_2O (1.5 equiv.) was then introduced at the same temperature and 15 min later, the syn-2,3-dimethyloxirane (1 equiv.) was added. The reaction mixture was stirred at –78 °C for 2 h before quenching with a saturated aqueous solution of NH_4Cl. The mixture was allowed to warm to room temperature and extracted with Et_2O. The combined extracts were washed with brine, dried over sodium sulfate, and concentrated under reduced pressure. The crude mixture was then dissolved in DCM and TESCl (1.2 equiv.) and imidazole (1.5 equiv.) were added. The mixture was stirred at 30 °C. Upon completion of the reaction by TLC analysis of hydrolyzed aliquots, (EtOAc/petroleum ether 20/80, $KMnO_4$ or *p*-anisaldehyde), the reaction was diluted with petroleum ether, filtered over celite and concentrated in vacuo. Silica-gel chromatography of the residue produced internal alkyne **SI-3**.

A flame-dried round bottom flask was charged with internal alkyne **SI-3** (1.3 equiv.), Rh_2OAc_4 (0.5 mol%) and dry dichloromethane under an argon atmosphere. A solution of ethyl diazoacetate in dry dichloromethane (1.0 equiv.) was added over a period of 15 h via a syringe pump at ambient temperature. After further 2 h of stirring, the solvent was removed under reduced pressure and the reaction mixture was directly transferred to silica gel column chromatography to yield the pure product **SI-4** as a yellowish oil.

Triethyl(((2R,3S)-3-methylnon-4-yn-2-yl)oxy)silane (SI -3)

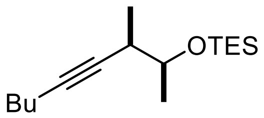

Scale, physical description, yield, mass: 70 mmol, yellowish oil, 80% yield, 14.95 g. **Flash chromatography:** 0 to 2 % EtOAc / petroleum ether. **^{1}H NMR (400 MHz, $CDCl_3$) δ:** 3.85 (qd, J = 6.2, 4.6 Hz, 1H), 2.48 (ttd, J = 7.0, 4.7, 2.3 Hz, 1H), 2.15 (td, J = 6.9, 2.2 Hz, 2H), 1.52 – 1.32 (m, 4H), 1.15 (d, J = 6.2 Hz, 3H), 1.09 (d, J = 7.0 Hz, 3H), 1.02 – 0.84 (m, 12H), 0.59 (q, J = 7.6 Hz, 6H). **^{13}C NMR (101 MHz, $CDCl_3$) δ**: 82.6, 81.5, 70.6, 33.8, 31.2, 21.9, 19.4, 18.5, 15.0, 13.7, 6.9, 4.9. **HRMS** (APCI): m/z calculated for $C_{16}H_{33}OSi$ $[M+H]^+$: 269.2301, found 269.2288.

Ethyl 2-butyl-3-((2S,3R)-3-((triethylsilyl)oxy)butan-2-yl)cycloprop-2-enecarboxylate (SI -4)

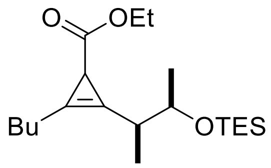

Note: this compound is obtained as a 1:1 mixture of diastereomers.

Scale, physical description, yield, mass: 55 mmol, yellowish oil, 6% yield, 1.28 g. **Flash chromatography:** 0 to 3 % EtOAc / petroleum ether. **^{1}H NMR (400 MHz, $CDCl_3$) δ:** 4.09 (q, J = 7.1 Hz, 2H), 4.02 (dd, J = 6.3, 3.5 Hz, 0.5H), 3.94 (dd, J = 6.2, 3.9 Hz, 0.5H), 2.71 (td, J = 6.6, 3.3 Hz, 1H), 2.41 (td, J = 7.3, 1.4 Hz, 2H), 2.03 (t, J = 4.6 Hz, 1H), 1.60 – 1.46 (m, 2H), 1.43 – 1.27 (m, 2H), 1.22 (t, J = 7.1 Hz, 3H), 1.19 – 1.01 (m, 6H), 1.00 – 0.83 (m, 13H), 0.75 – 0.45 (m, 6H). **^{13}C NMR (101 MHz, $CDCl_3$) δ**: 177.1, 176.9, 107.5, 107.3, 106.6, 106.5, 69.8, 69.6, 59.7, 38.2, 38.0, 29.2, 29.1, 24.3, 24.3, 22.4, 22.3, 21.9, 20.2, 19.8, 14.4, 13.8, 12.9, 6.9, 4.99, 4.96. **HRMS** (APCI): m/z calculated for $C_{20}H_{39}O_3Si$ $[M+H]^+$: 355.2668, found 355.2679.

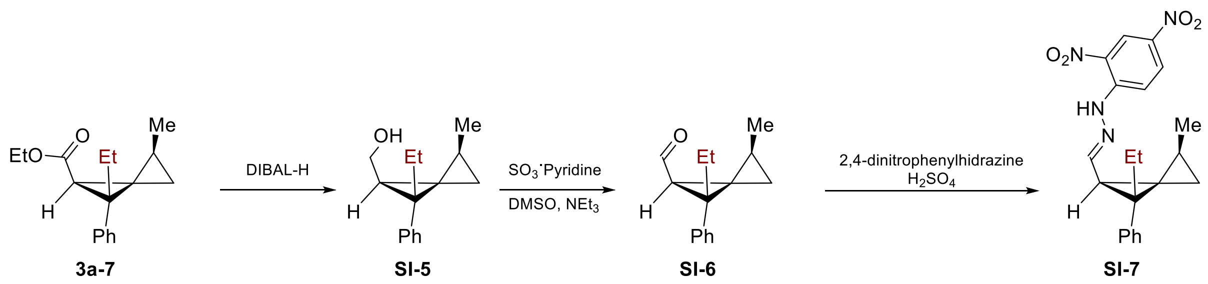

In a flame dried three necked flask equipped with an addition funnel, ester **3a-7** (1 equiv.) was dissolved in Et_2O (1 M) and cooled to -30 ℃. DIBAL-H (2.2 equiv., 1 M in hexane) was added dropwise from the addition funnel. The reaction mixture was stirred at -30 ℃ until full consumption of cyclopropenyl esters was observed by TLC analysis of hydrolyzed aliquots (15% EtOAc/ petroleum ether, p-anisaldehyde/$KMnO_4$, ca. 1 h). DIBAL-H like most aluminum compound reacts violently with air and water. Therefore, a careful hydrolysis should be performed at low temperature. At -30 ℃, the reaction was therefore diluted with a solution of Et_2O mixed with an aqueous solution of 1 M HCl (40%). The reaction mixture was stirred until two separated phases appeared. The aqueous layer was extracted three times with Et_2O, and the combined organic phases were dried over Na_2SO_4, filtered, and concentrated under reduced pressure. Crude mixture was then purified by flash chromatography using EtOAc / petroleum ether as eluent, producing **SI-5**.

In a round bottom flask, open to air, **SI-5** was dissolved in a 1:1 mixture of DCM:DMSO (0.1 M). After cooling to 0 °C, triethylamine and SO_3·pyridine complex were added sequentially. The reaction was stirred at room temperature until full consumption of the starting material was observed by TLC analysis of hydrolyzed aliquots (15% EtOAc/ petroleum ether, p-anisaldehyde/$KMnO_4$). The reaction was diluted with DCM and quenched with 1 M HCl. The mixture was stirred until two separated phases appeared. The aqueous layer was extracted three times with DCM, and the combined organic phases were dried over Na_2SO_4, filtered, and concentrated under reduced pressure. The crude reaction mixture was then purified by flash chromatography using EtOAc / petroleum ether as eluent, producing **SI-6**.

In a round bottom flask, open to air, 2,4-dinitrophenyl hydrazine (1.2 equiv.) in ethanol (3 mL) was added to the aldehyde **SI-6** (1 equiv.). One drop of concentrated sulfuric acid was added and the reaction was stirred at ambient temperature for 24 h. Ethyl acetate and water were then added. The organic fraction was washed with an aqueous saturated solution of sodium hydrogen carbonate and brine, dried over anhydrous sodium sulfate, filtered and concentrated in vacuo to an orange oil. Purification by flash column chromatography gave hydrazone **SI-7** as a orange solid which was recrystallized from 1/2 DCM/*n*-pentane to give orange crystals suitable for X-ray analysis

((1S,2S,3R,4S)-2-ethyl-4-methyl-2-phenylspiro[2.2]pentan-1-yl)methanol (SI -5)

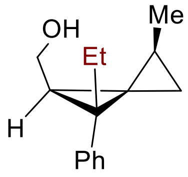

Scale, physical description, yield, mass: 1.3 mmol, colorless oil, 41% yield, 110 mg. **Flash chromatography:** 0 to 15 % EtOAc / petroleum ether. **^{1}H NMR (400 MHz, $CDCl_3$) δ:** 7.35 – 7.28 (m, 2H), 7.25 – 7.17 (m, 3H), 3.95 (dd, J = 11.2, 7.6 Hz, 1H), 3.80 (dd, J = 11.2, 6.8 Hz, 1H), 1.92 (dq, J = 14.7, 7.4 Hz, 1H), 1.71 (ddd, J = 11.1, 7.3, 3.7 Hz, 2H), 1.34 – 1.29 (m, 1H), 1.27 (d, J = 5.4 Hz, 3H), 0.85 (t, J = 7.4 Hz, 3H), 0.75 (dd, J = 7.4, 4.5 Hz, 1H), 0.43 (t, J = 4.4 Hz, 1H). **^{13}C NMR (101 MHz, $CDCl_3$) δ**: 144.6, 129.1, 128.0, 125.9, 62.5, 35.5, 29.99, 29.96, 26.3, 17.6, 12.7, 12.1, 11.5. **HRMS** (APCI): m/z calculated for $C_{15}H_{19}O$ $[M-H]^+$: 215.1430, found 215.1426.

(1S,2S,3R,4S)-2-ethyl-4-methyl-2-phenylspiro[2.2]pentane-1-carbaldehyde (SI -6)

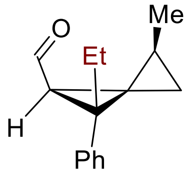

Scale, physical description, yield, mass: 0.5 mmol, colorless oil, quantitative yield, 110 mg. **Flash chromatography:** 0 to 5 % EtOAc / petroleum ether. **^{1}H NMR (400 MHz, $CDCl_3$) δ:** 9.46 (d, J = 6.5 Hz, 1H), 7.26 (t, J = 7.7 Hz, 2H), 7.22 – 7.16 (m, 1H), 7.16 – 7.11 (m, 2H), 2.30 (d, J = 6.5 Hz, 1H), 2.03 (q, J = 7.3 Hz, 2H), 1.54 (ddd, J = 13.9, 6.8, 4.2 Hz, 1H), 1.32 (d, J = 6.0 Hz, 3H), 0.83 – 0.72 (m, 4H), 0.50 (t, J = 5.1 Hz, 1H). **^{13}C NMR (101 MHz, $CDCl_3$) δ**: 202.2, 142.4, 128.6, 128.4, 126.7, 44.4, 39.7, 35.4, 26.7, 17.1, 13.0, 11.9, 11.9. **HRMS** (APCI): m/z calculated for $C_{15}H_{19}O$ $[M+H]^+$: 215.1430, found 215.1431.

(2,4-dinitrophenyl)-2-(((1S,2S,3R,4S)-2-ethyl-4-methyl-2-phenylspiro[2.2]pentan-1-yl)methylene)hydrazine (SI-7)

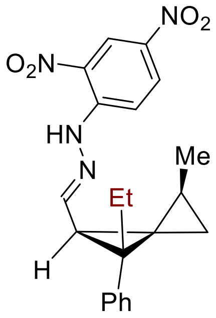

Scale, physical description, yield, mass: 1 mmol, orange solid, 22% yield, 89 mg. **Flash chromatography:** 0 to 5 % EtOAc / petroleum ether. **^{1}H NMR (400 MHz, $CDCl_3$) δ:** 11.18 (s, 1H), 9.14 (d, J = 2.6 Hz, 1H), 8.31 (dd, J = 9.6, 2.6 Hz, 1H), 7.96 (d, J = 9.5 Hz, 1H), 7.90 (d, J = 9.6 Hz, 1H), 7.38 – 7.31 (m, 2H), 7.30 – 7.23 (m, 3H), 6.43 (dt, J = 9.5, 2.5 Hz, 1H), 3.16 (ddd, J = 16.8, 8.5, 2.5 Hz, 1H), 2.57

– 2.44 (m, 1H), 2.39 (ddd, J = 16.8, 6.0, 2.6 Hz, 1H), 2.15 (dd, J = 14.1, 7.2 Hz, 1H), 2.02 (dt, J = 14.1, 7.2 Hz, 1H), 0.85 (t, J = 7.3 Hz, 3H), 0.73 (d, J = 7.0 Hz, 3H). **^{13}C NMR (101 MHz, $CDCl_3$) δ**: 162.1, 146.8, 144.7, 140.5, 130.0, 128.1, 127.4, 126.3, 123.6, 119.9, 116.7, 59.9, 37.1, 34.0, 33.9, 18.6, 9.7. **HRMS** (APCI): m/z calculated for $C_{21}H_{23}N_4O_4$ $[M+H]^+$: 395.1714, found 395.1721. **CDCC**: 2172799**.**

14. Crystal data and structure refinement for compounds **1a-8**, **SI-7**.

Crystallographic data are summarized in Table S1. Additionally, complete data have been deposited with the Cambridge Crystallographic Data Centre under the number:

CCDC 2172800 (**1a-8**), 2172799 (**SI-7**).

Copies of the data can be obtained free of charge from www.ccdc.cam.ac.uk/data_request/cif.

Table S1: Crystallographic data and structure refinement details

Compound	**1a-8**	**SI-7**
CCDC number	2172800	2172799
Formula	$C_{22}H_{24}O_5S$	$C_{21}H_{22}N_4O_4$
M_r	400.47	394.42
Cryst. size (mm^3)	0.27 × 0.15 × 0.15	0.21 × 0.09 × 0.09
Crystal system	monoclinic	triclinic
Space group	$P2_1/n$	P-1
Temperature (°K)	200.15	200.15
a (Å)	8.6193(14)	6.4520(10)
b (Å)	28.606(5)	8.429(2)
c (Å)	9.1572(15)	19.580(3)
α (°)	90	98.552(5)
β (°)	116.113(4)	95.936(4)
γ (°)	90	100.229(3)
V (Å^3)	2027.3(6)	1027.0(3)
Z	4	2
D_x (Mg m^{-3})	1.312	1.275
λ (Å)	0.71073	0.71073
μ (mm^{-1})	0.190	0.090
F(000)	848.0	416.0
$2\theta_{max}$	50.254	50.29
Refl. measured	11893	14384
Refl. indep.	3562	2641
R_{int}	0.0417	0.0700
Parameters	256	330
$wR(F^2$, all refl.)	0.0698	0.1502
$R(F, >4\sigma(F))$	0.0959	0.0968
Max. Δ*p* (e Å^{-3})	0.15/-0.31	0.13/-0.16

1a-8 - 2172800

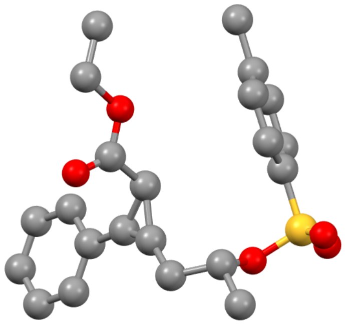

SI-7 - 2172799

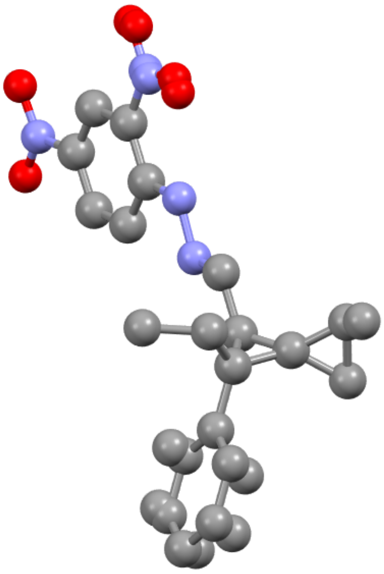

15. References

1. Bhattacharya, S.; Sengupta, S. *Tetrahedron Lett*. **2004**, *45*, 8733.

2. Sommer, H.; Hamiltob, J. Y.; Fürstner, A. *Angew. Chem. Int. Ed*. **2017**, *56*, 6161.

16. NMR spectra of starting cyclopropenes **5,1,6**.

(R)-ethyl 2-butyl-3-((R)-2-hydroxypropyl)cycloprop-2-enecarboxylate (5a-1)

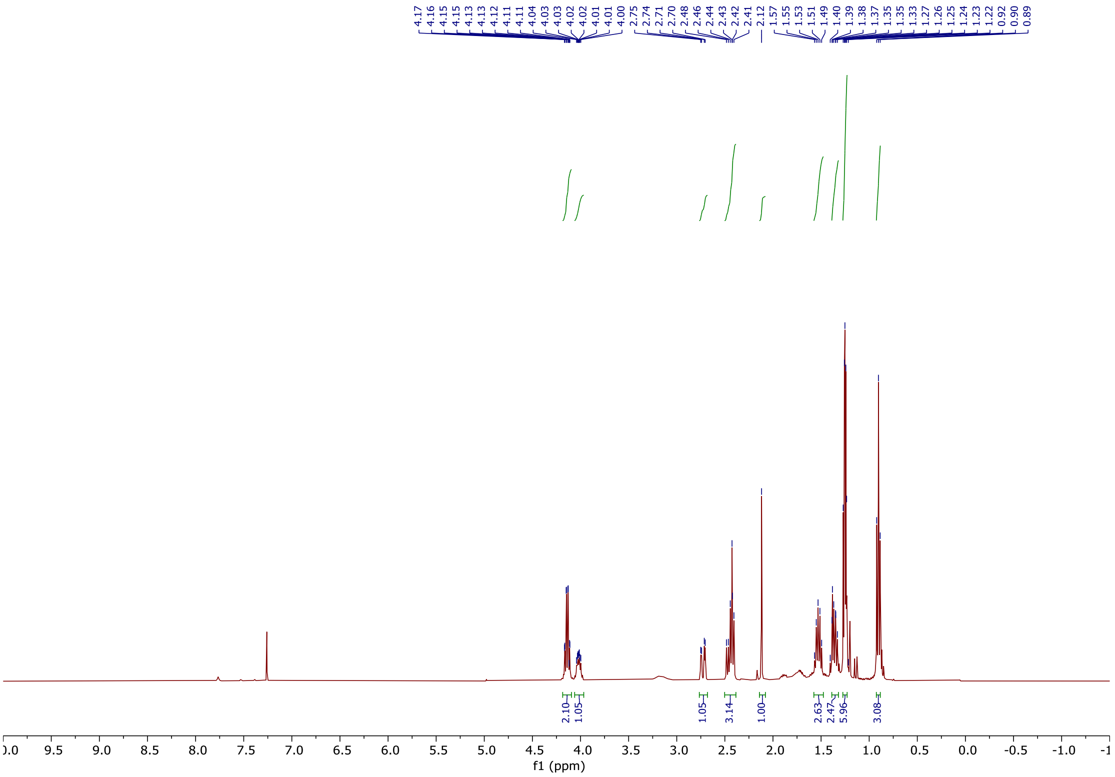

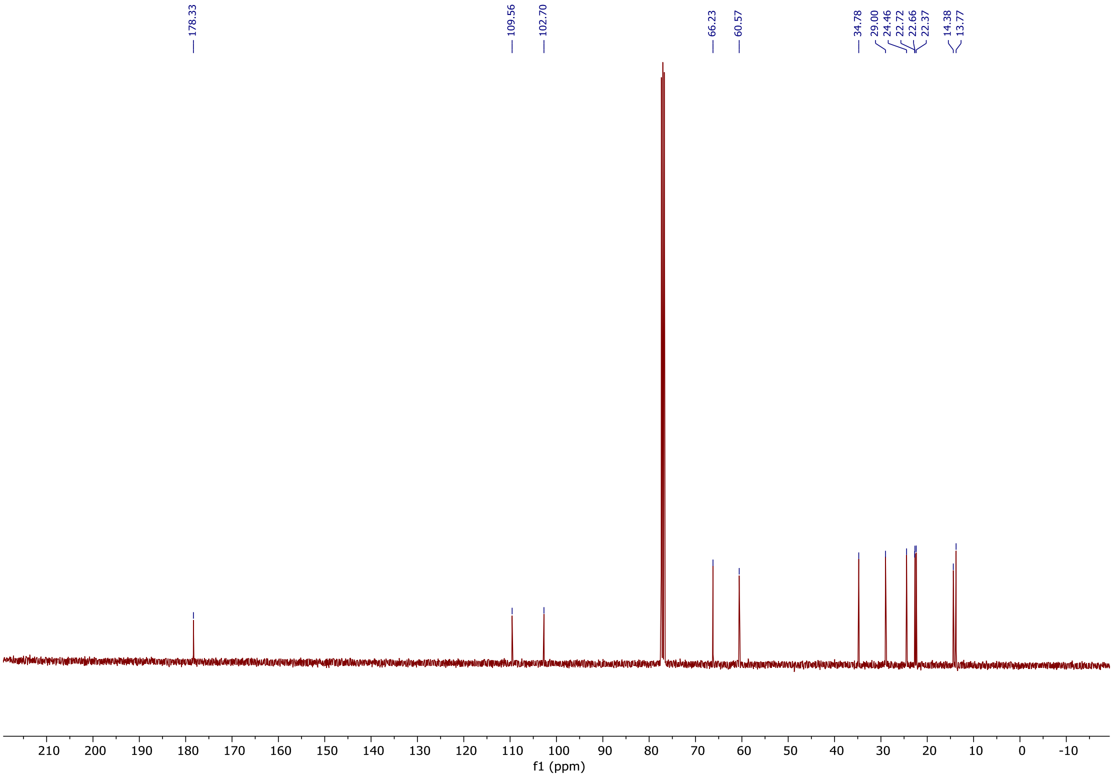

(S)-ethyl 2-butyl-3-((R)-2-hydroxypropyl)cycloprop-2-enecarboxylate (5b-1)

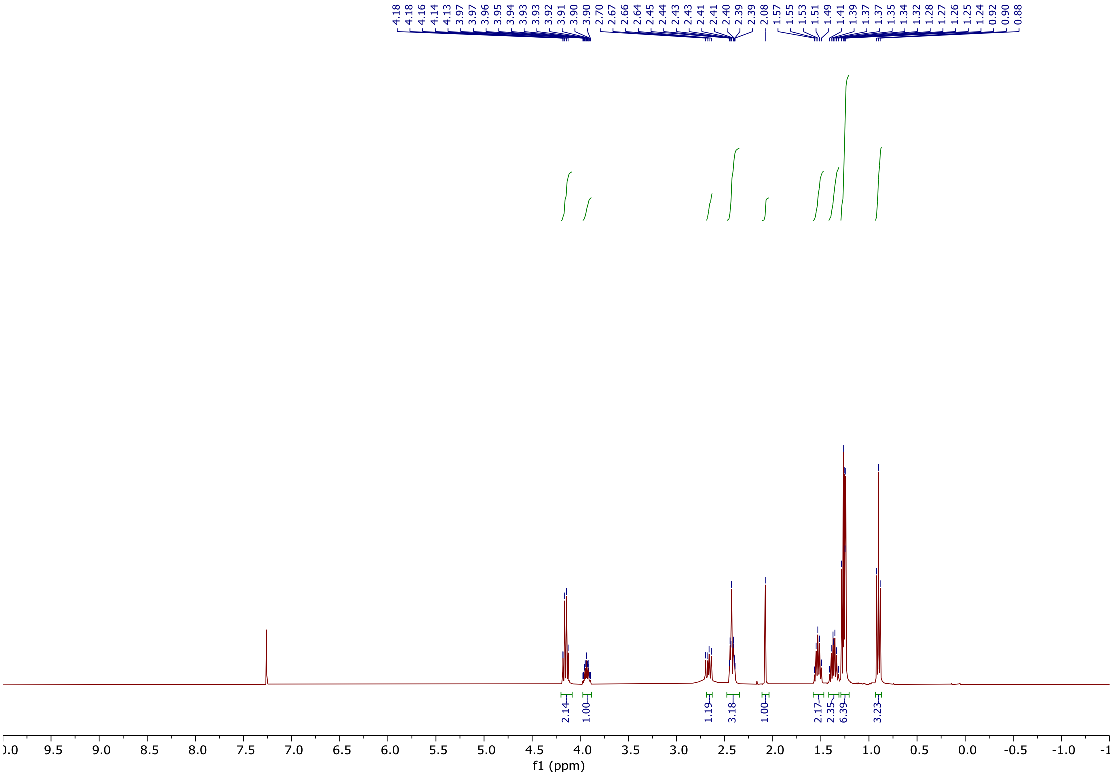

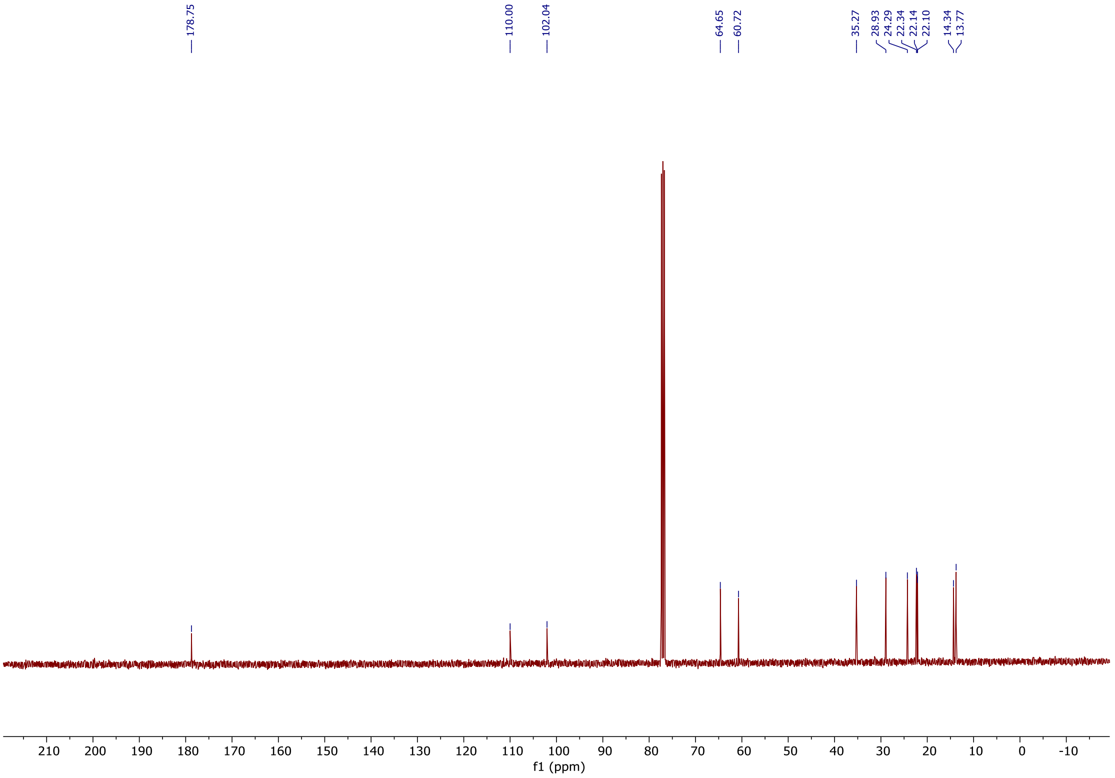

(R)-ethyl 2-ethyl-3-((R)-2-hydroxypropyl)cycloprop-2-enecarboxylate (5a-2)

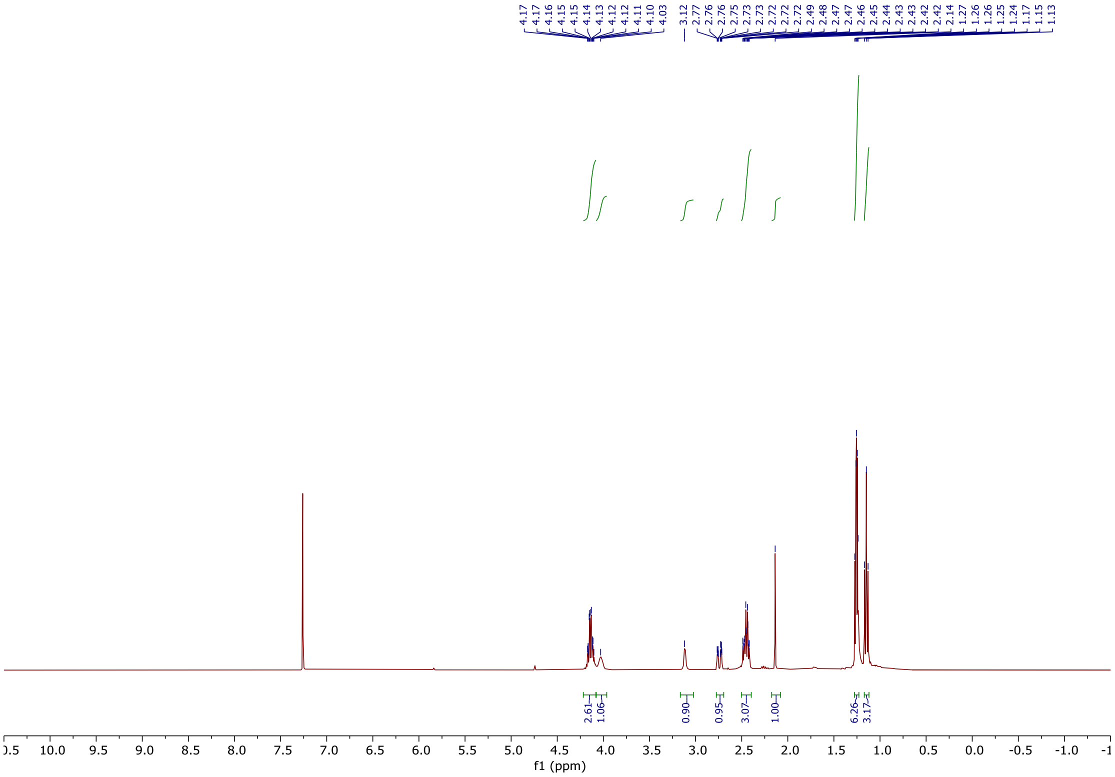

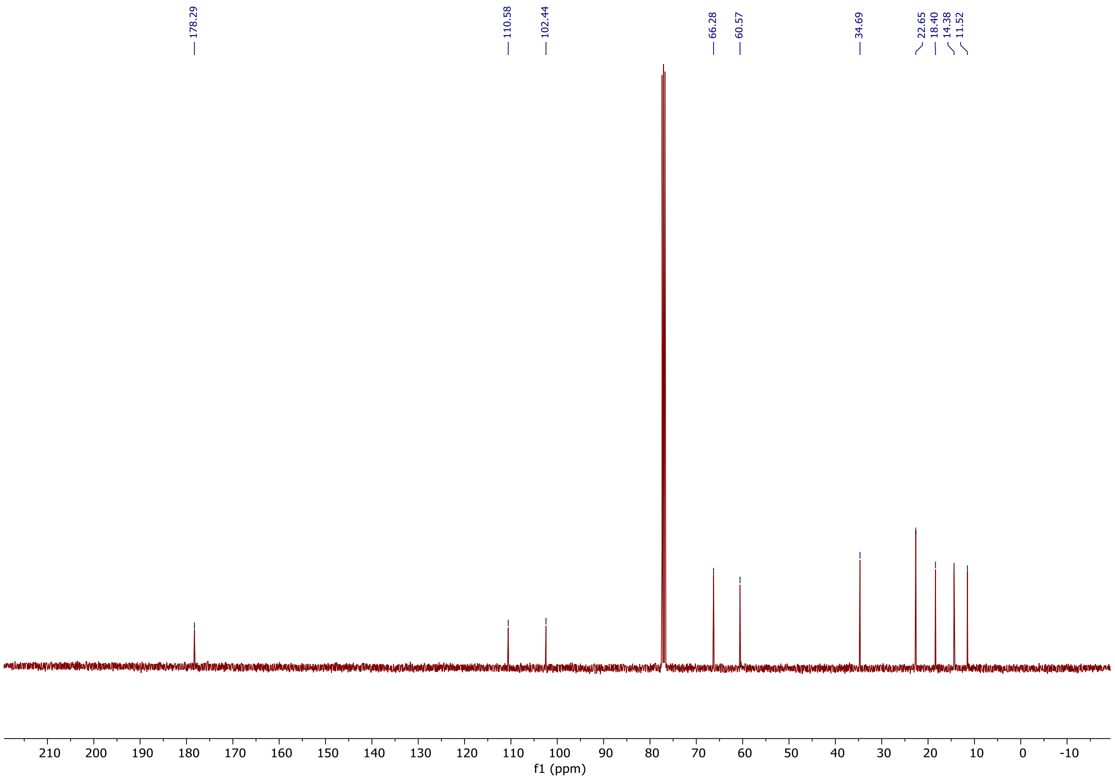

(S)-ethyl 2-cyclohexyl-3-((R)-2-hydroxypropyl)cycloprop-2-enecarboxylate (5a-3)

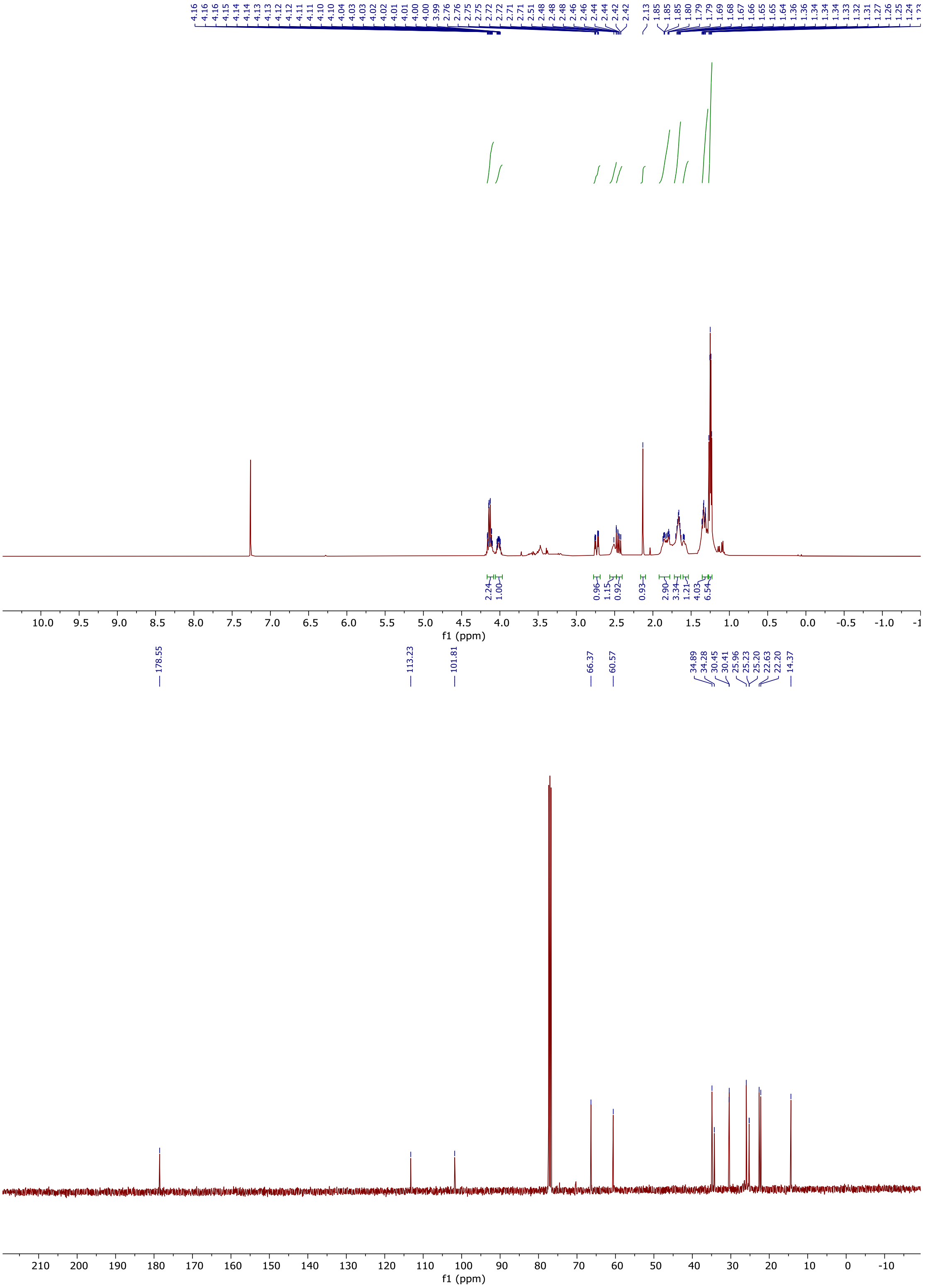

(R)-ethyl 2-((R)-2-hydroxypropyl)-3-phenethylcycloprop-2-enecarboxylate (5a-4)

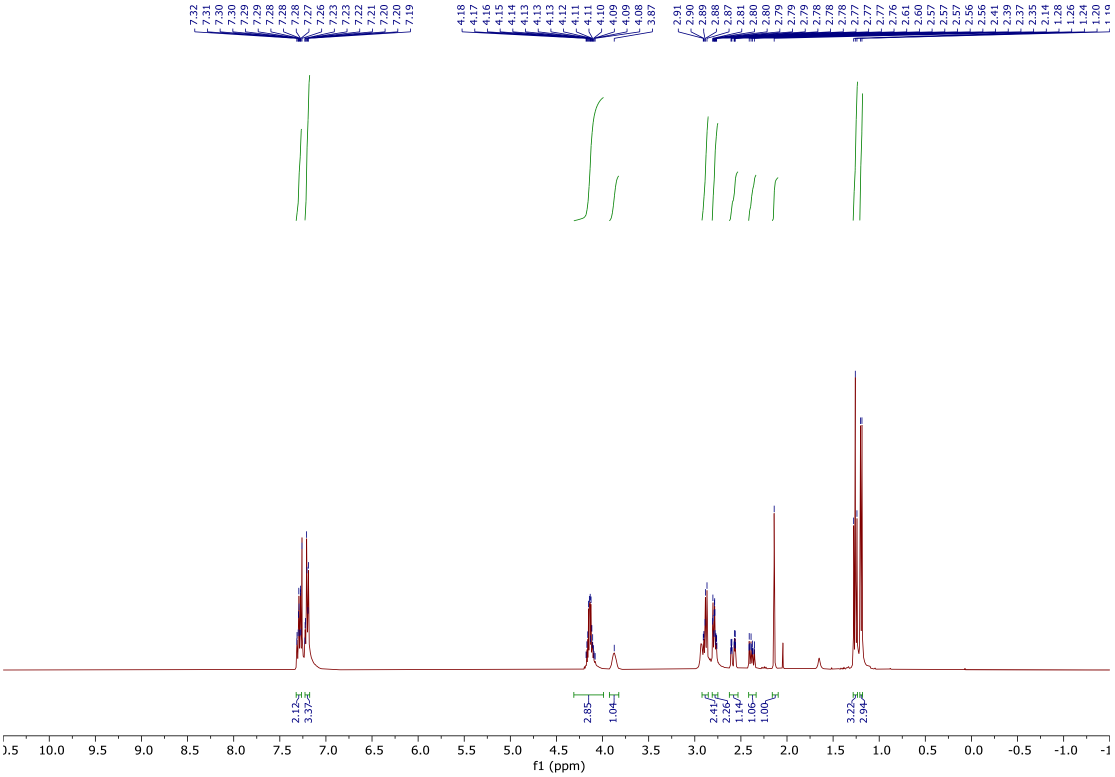

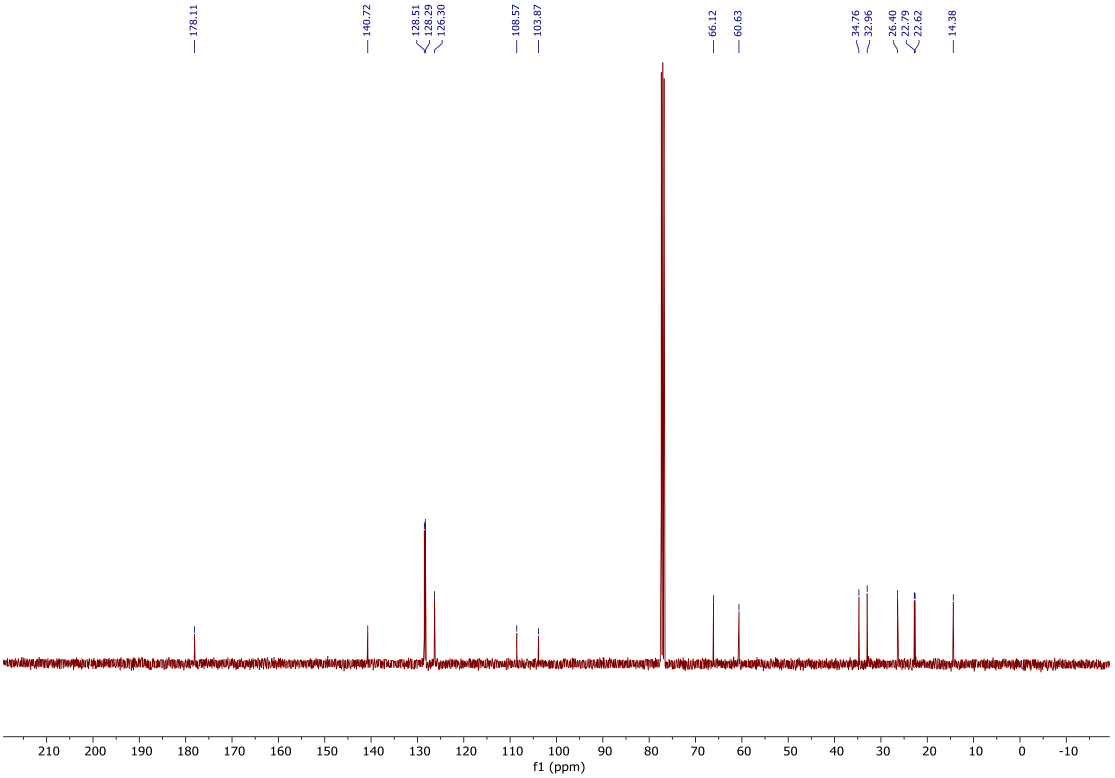

(R)-ethyl 2-(2-((tert-butyldimethylsilyl)oxy)ethyl)-3-((R)-2-hydroxypropyl)cycloprop-2-enecarboxylate (5a-5)

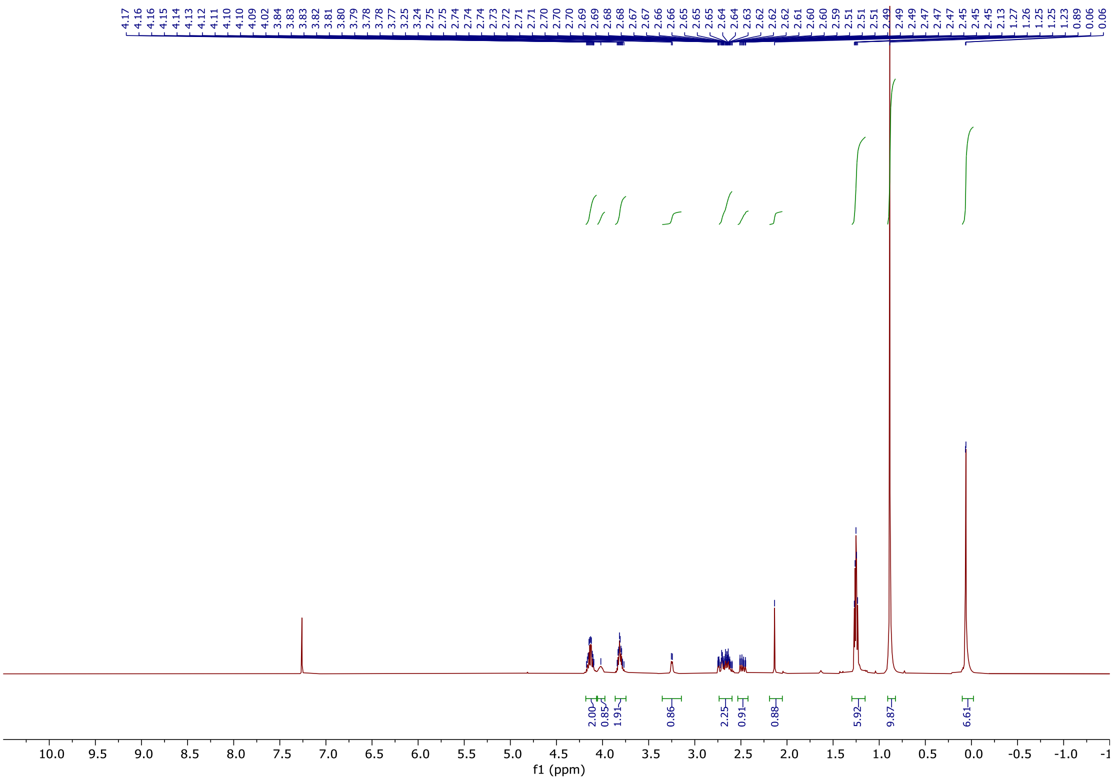

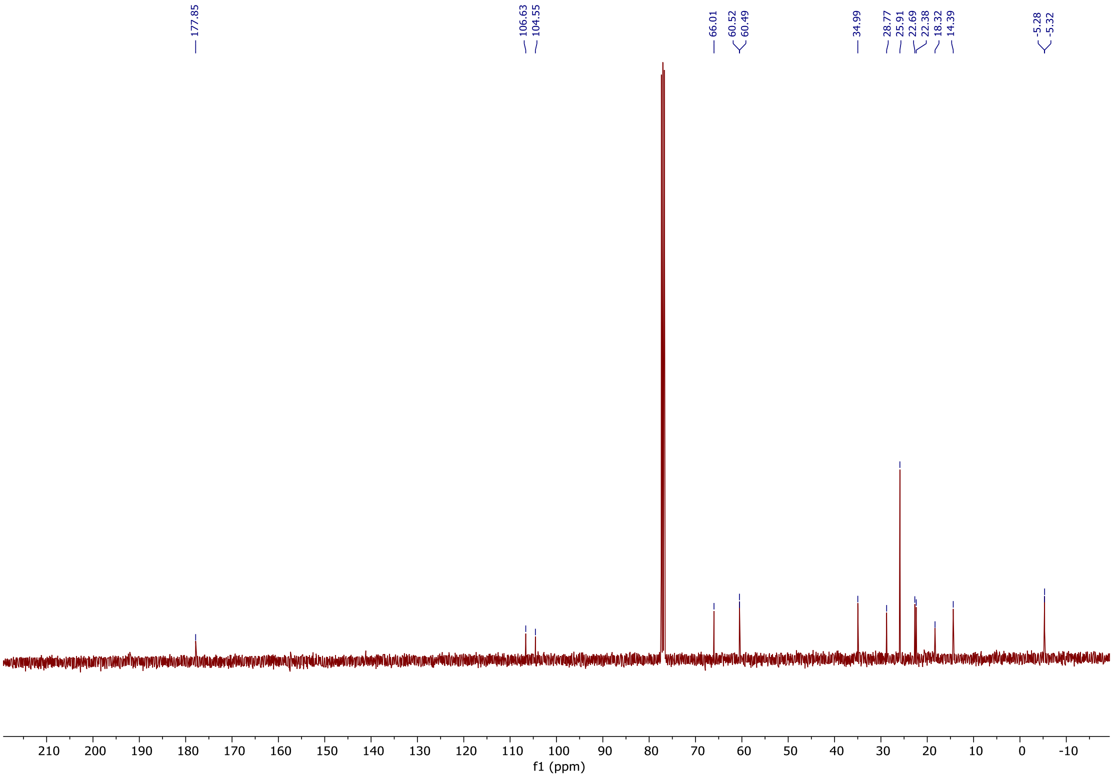

(S)-ethyl 2-(2-((tert-butyldimethylsilyl)oxy)ethyl)-3-((R)-2-hydroxypropyl)cycloprop-2-enecarboxylate (5b-2)

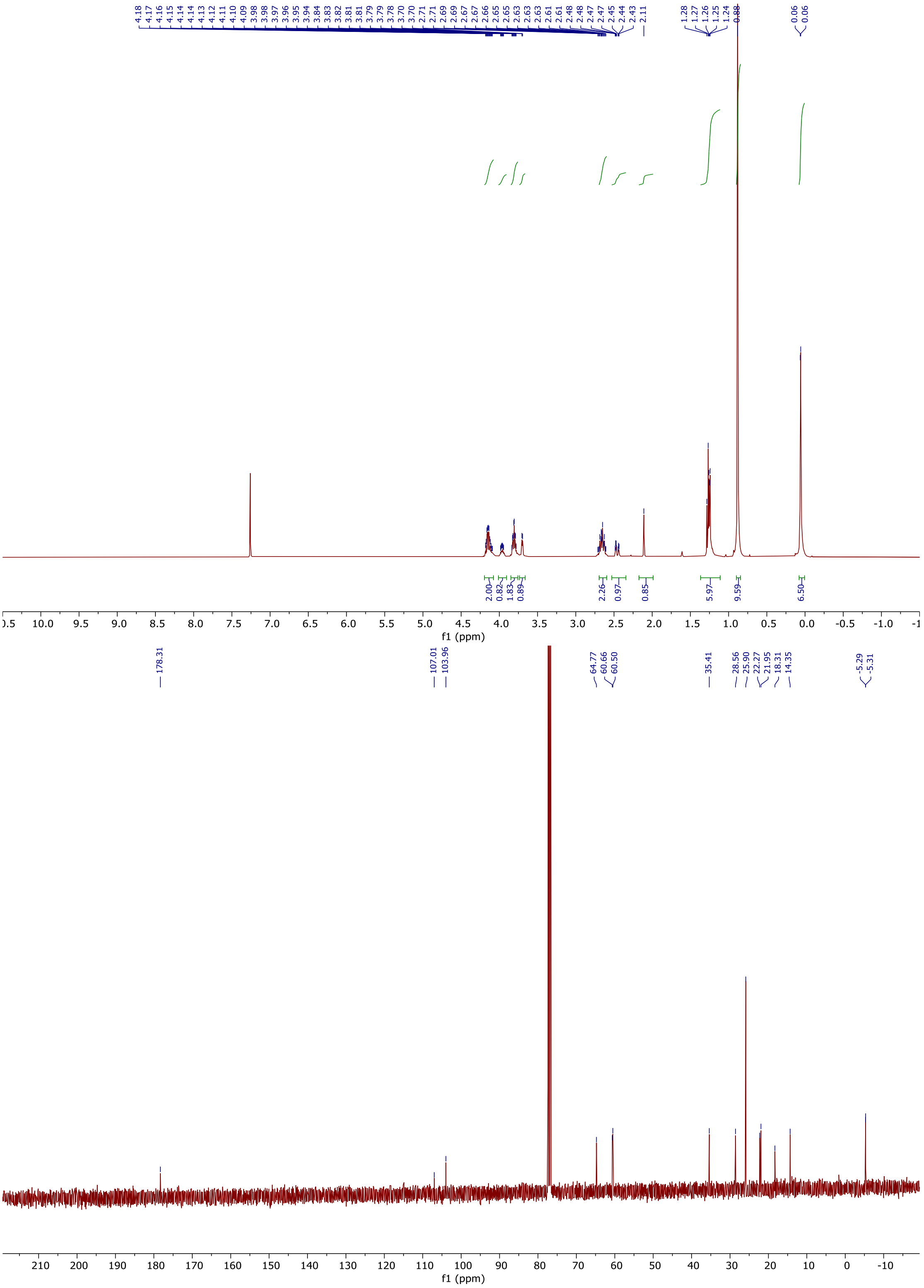

(R)-ethyl 2-butyl-3-((R)-2-hydroxybutyl)cycloprop-2-enecarboxylate (5a-6)

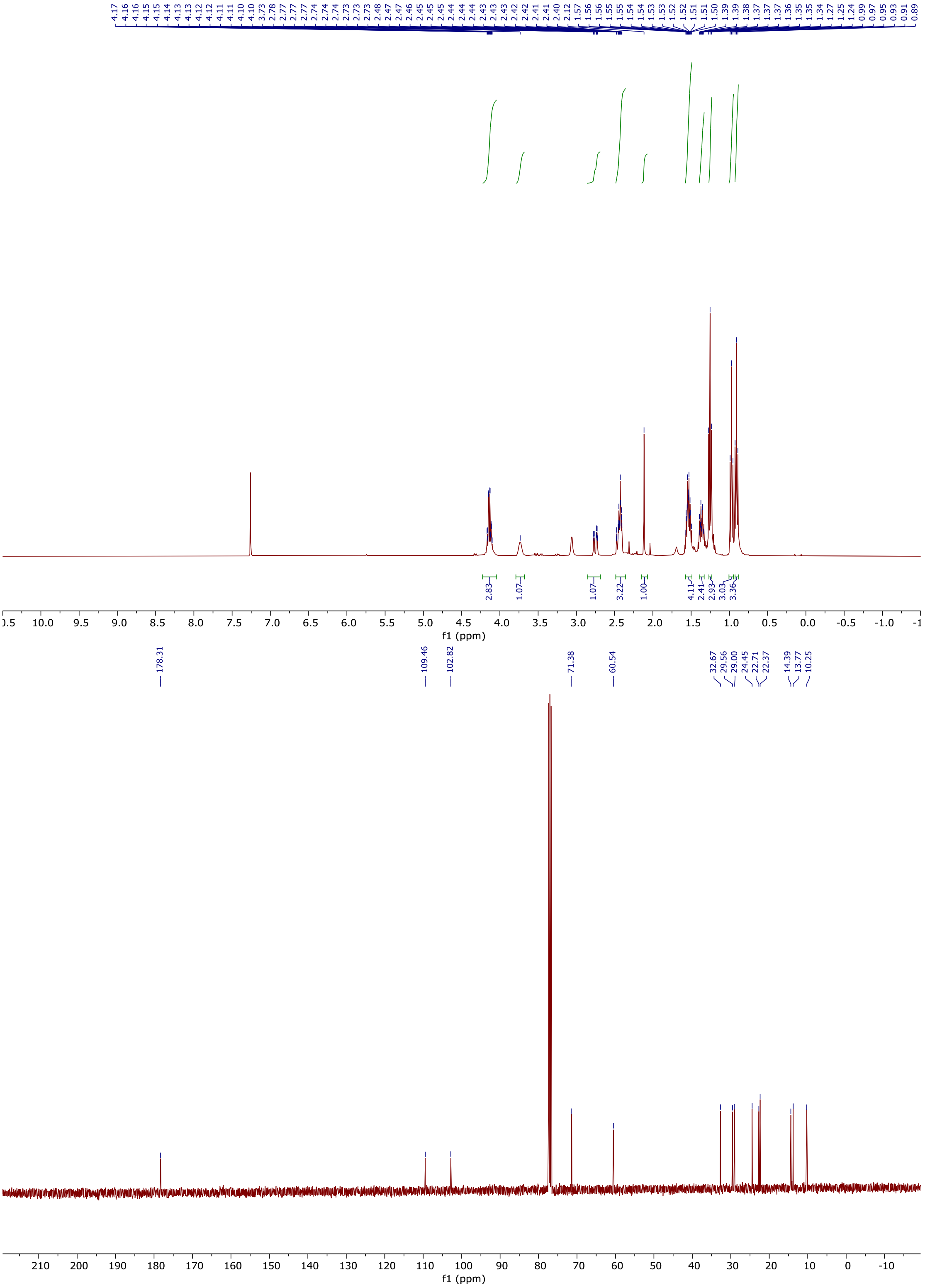

(R)-ethyl 2-butyl-3-((S)-2-hydroxy-3-methoxypropyl)cycloprop-2-enecarboxylate (5a-7)

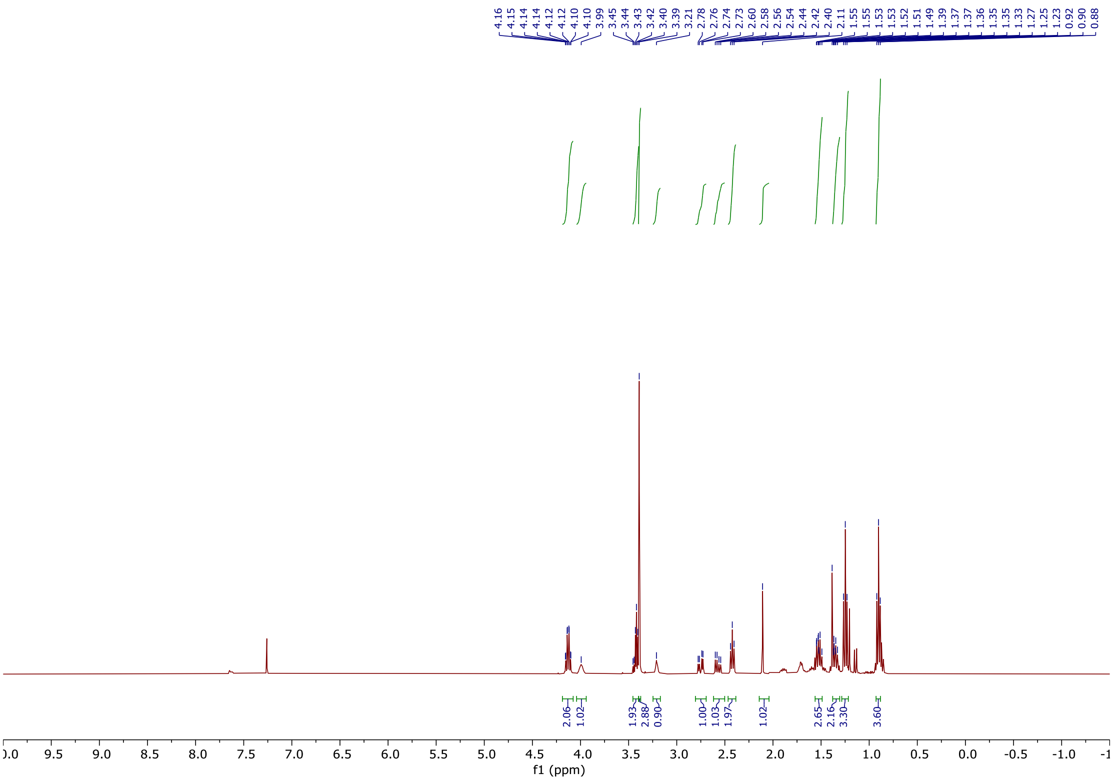

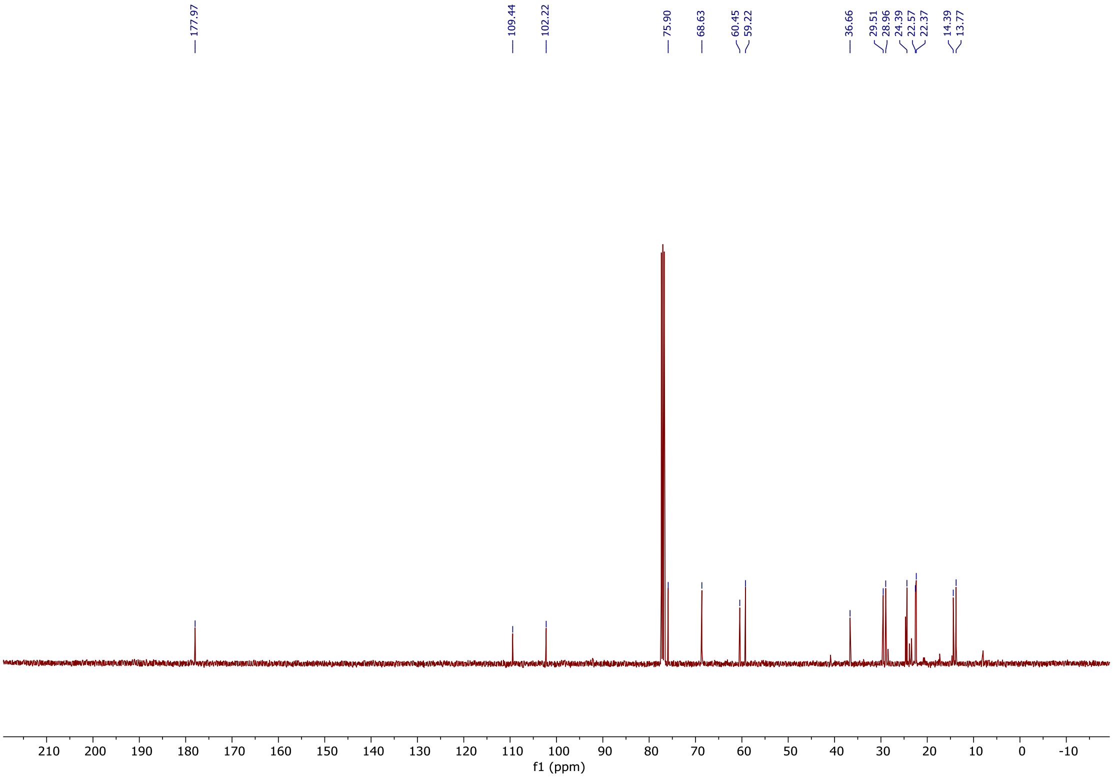

(S)-ethyl 2-((S)-2-hydroxy-3-methoxypropyl)-3-phenylcycloprop-2-enecarboxylate (5a-8)

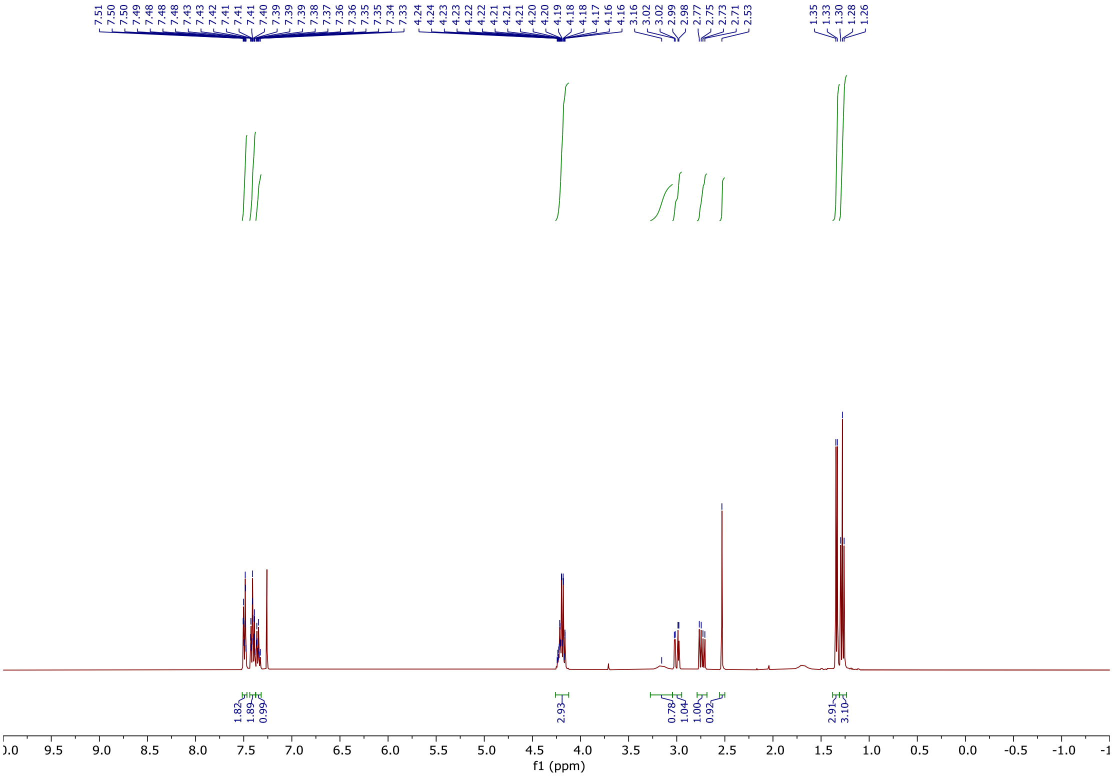

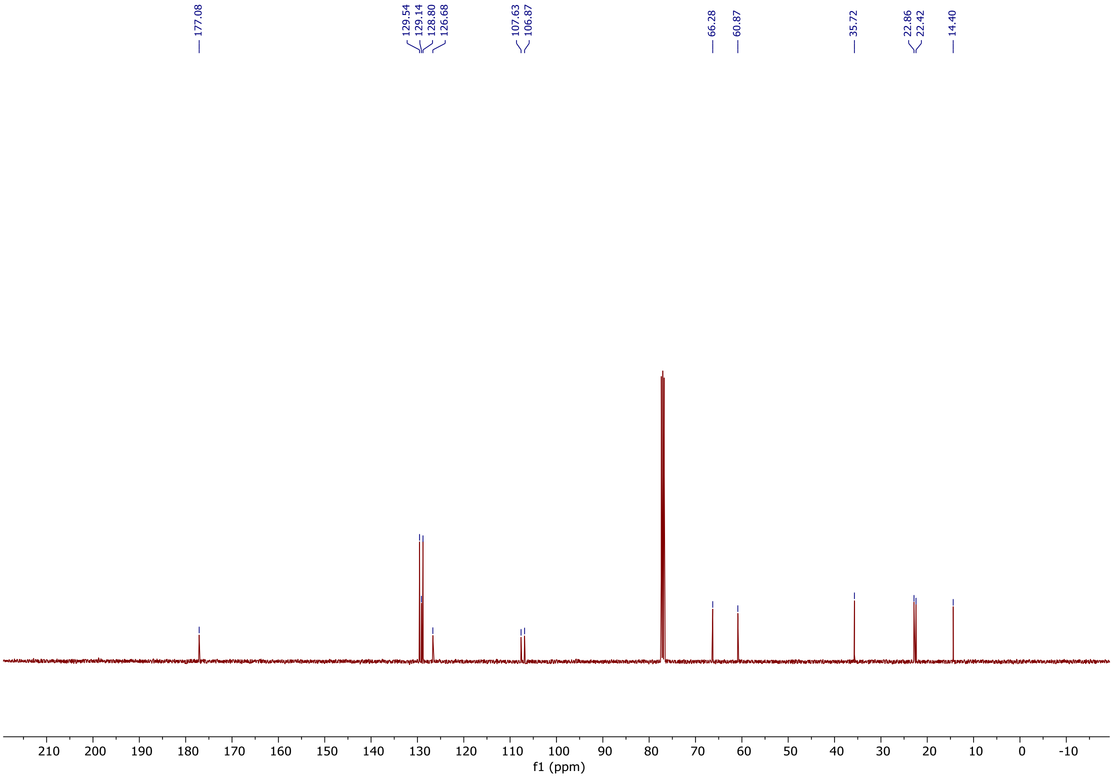

(R)-ethyl 2-butyl-3-((2S,3R)-3-hydroxybutan-2-yl)cycloprop-2-enecarboxylate (5a-9)

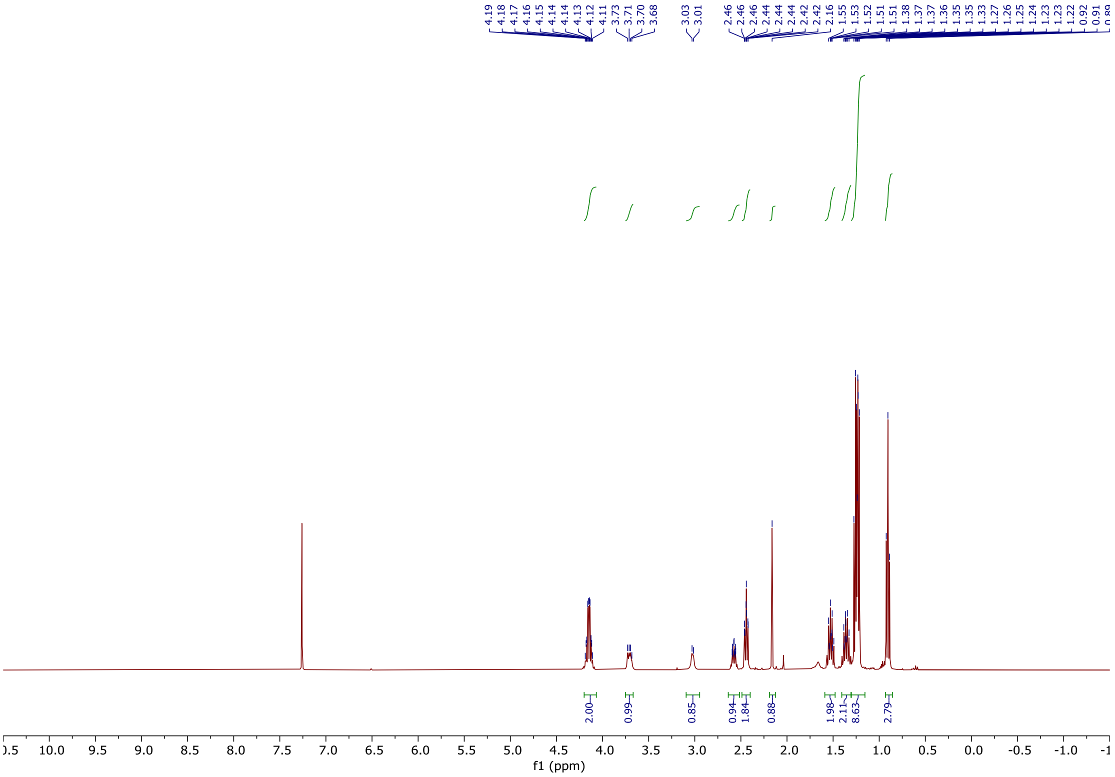

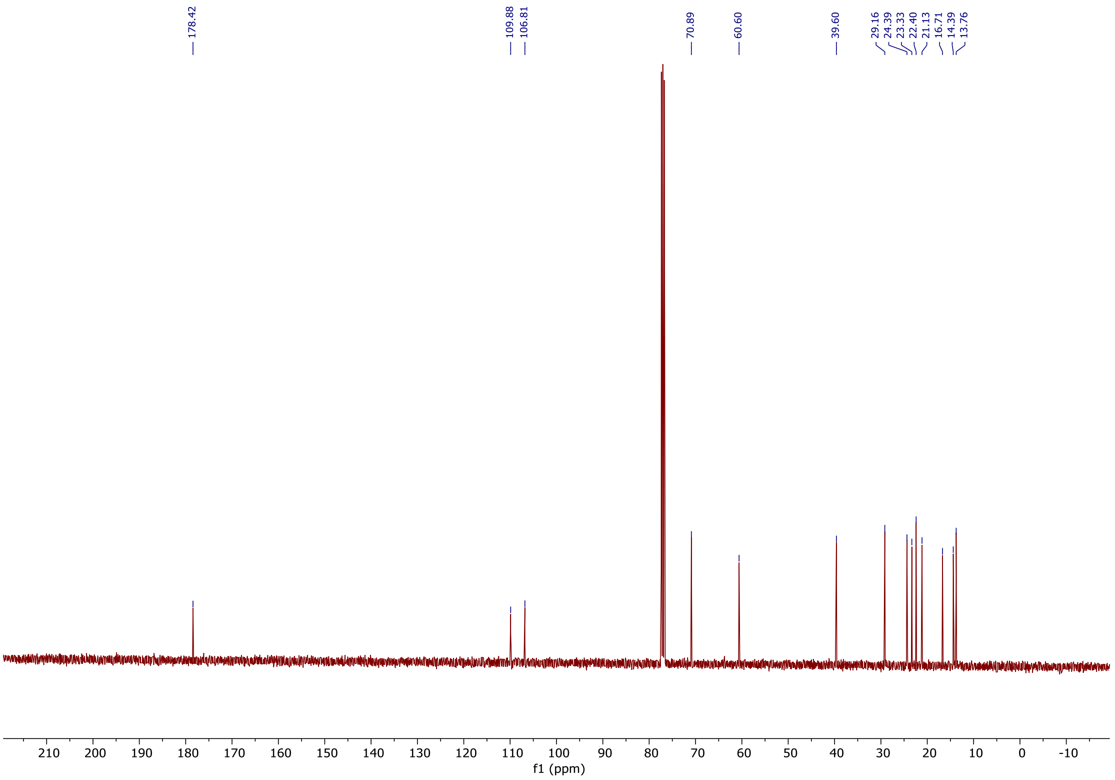

(R)-ethyl 2-butyl-3-((R)-2-hydroxypropyl)-1-methylcycloprop-2-enecarboxylate (5a-10)

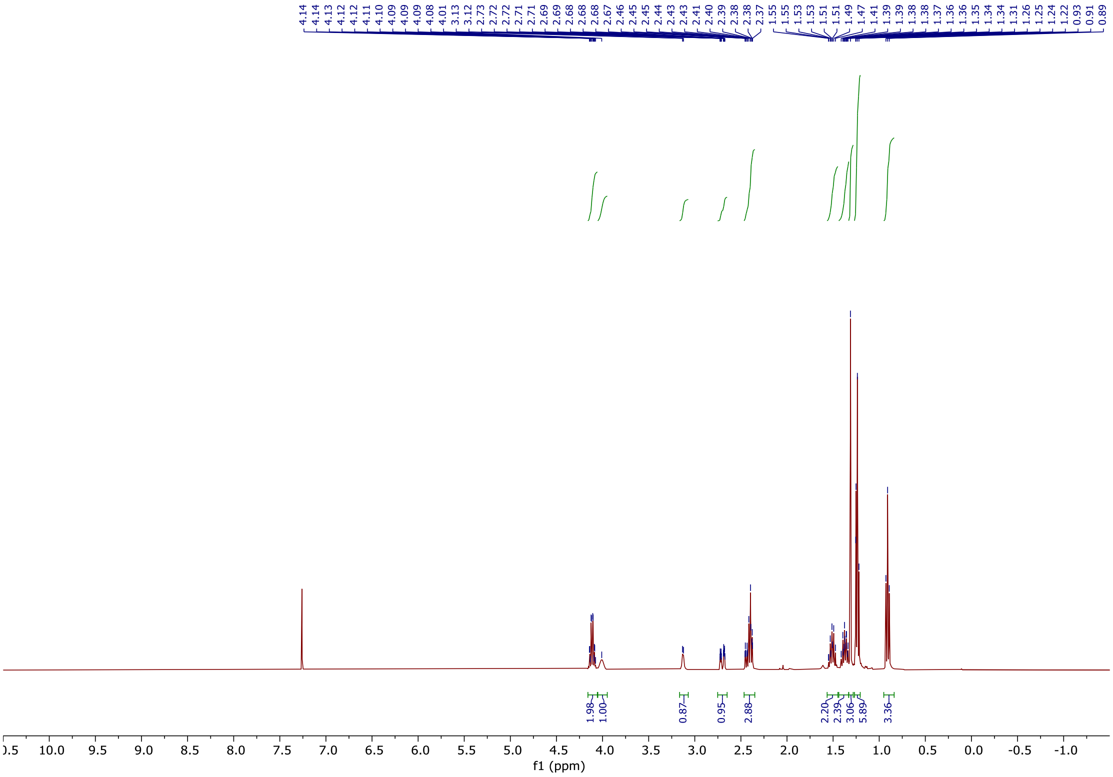

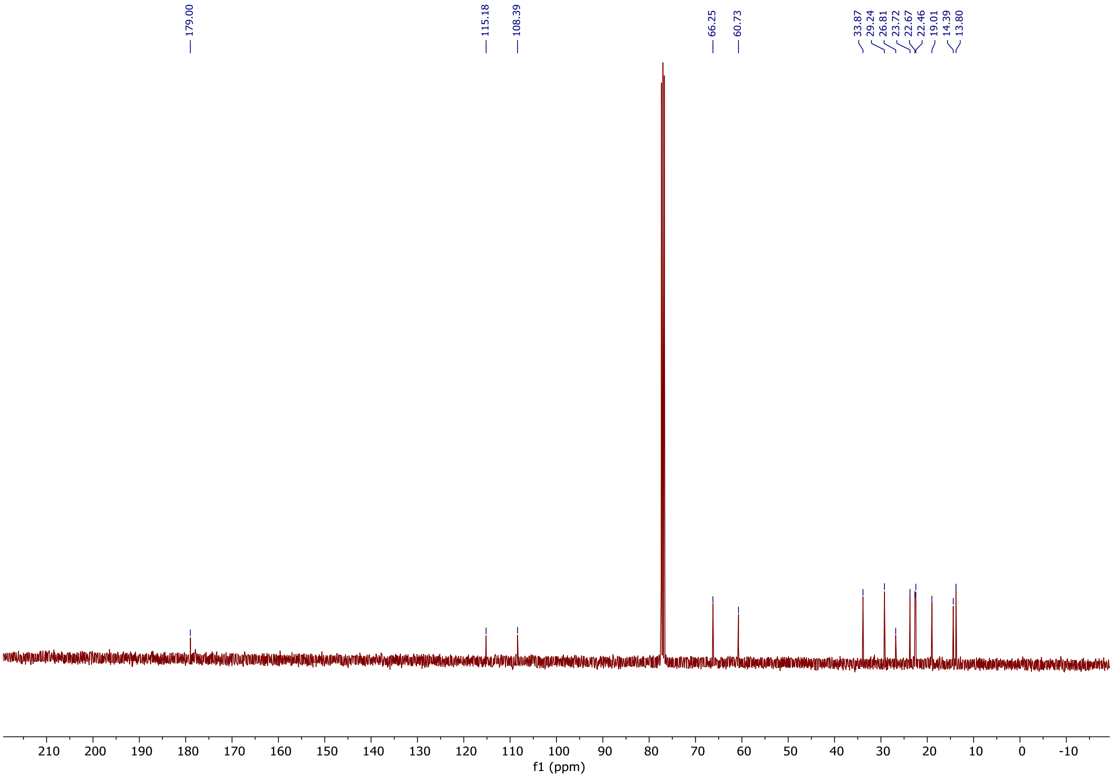

(R)-ethyl 2-butyl-3-((R)-2-(tosyloxy)propyl)cycloprop-2-enecarboxylate (1a-1)

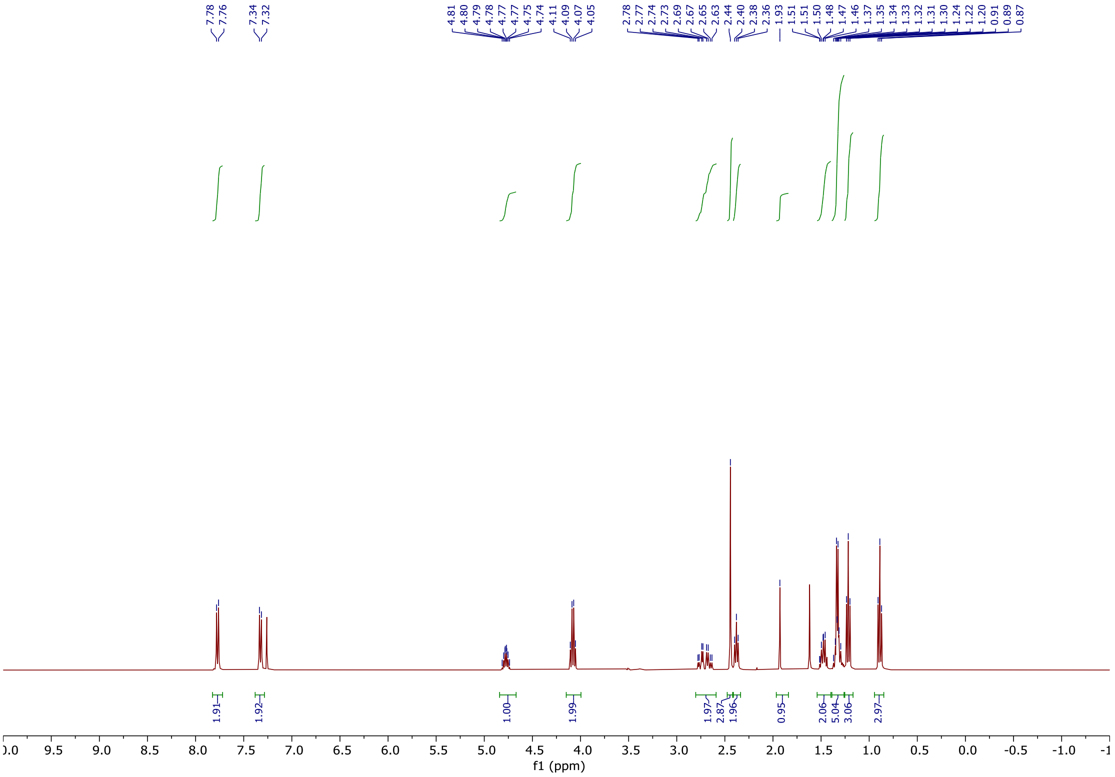

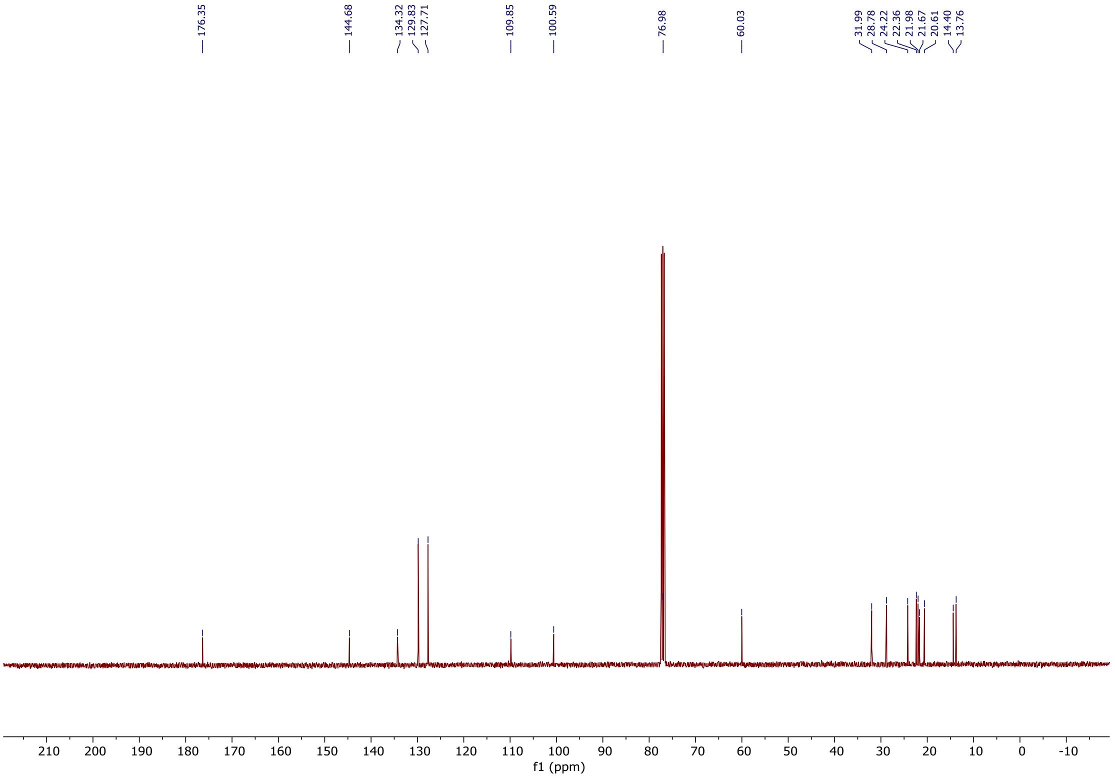

(S)-ethyl 2-butyl-3-((R)-2-(tosyloxy)propyl)cycloprop-2-enecarboxylate (1b-1)

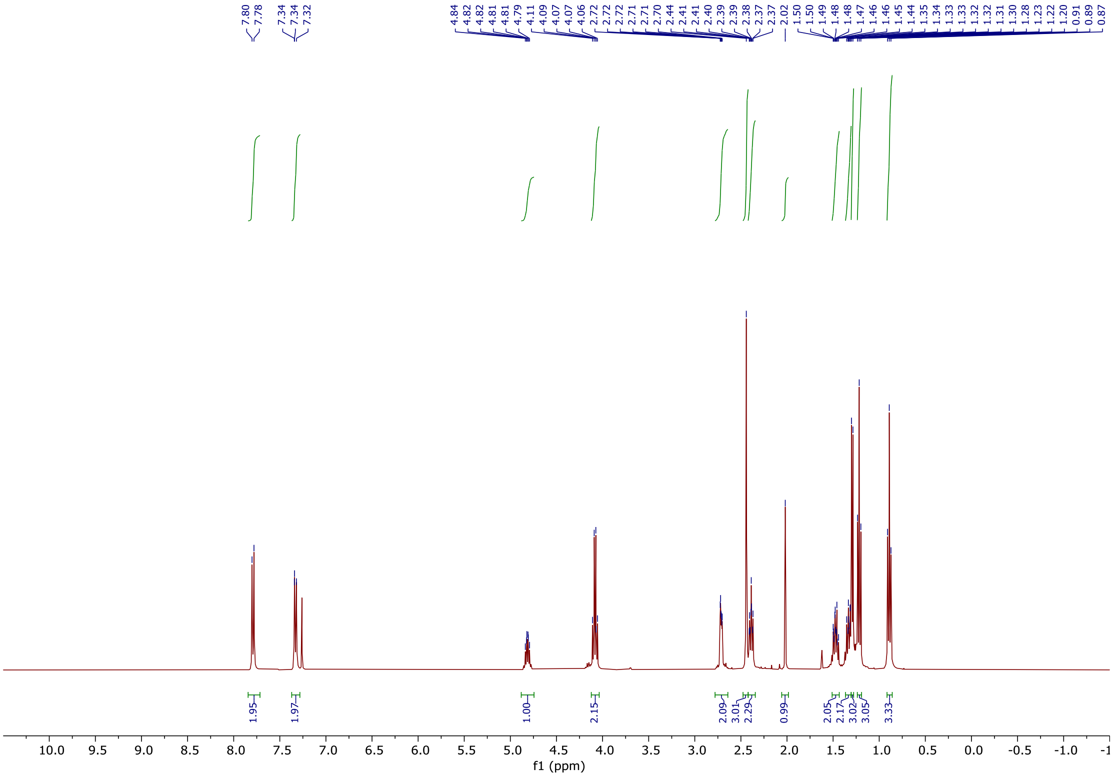

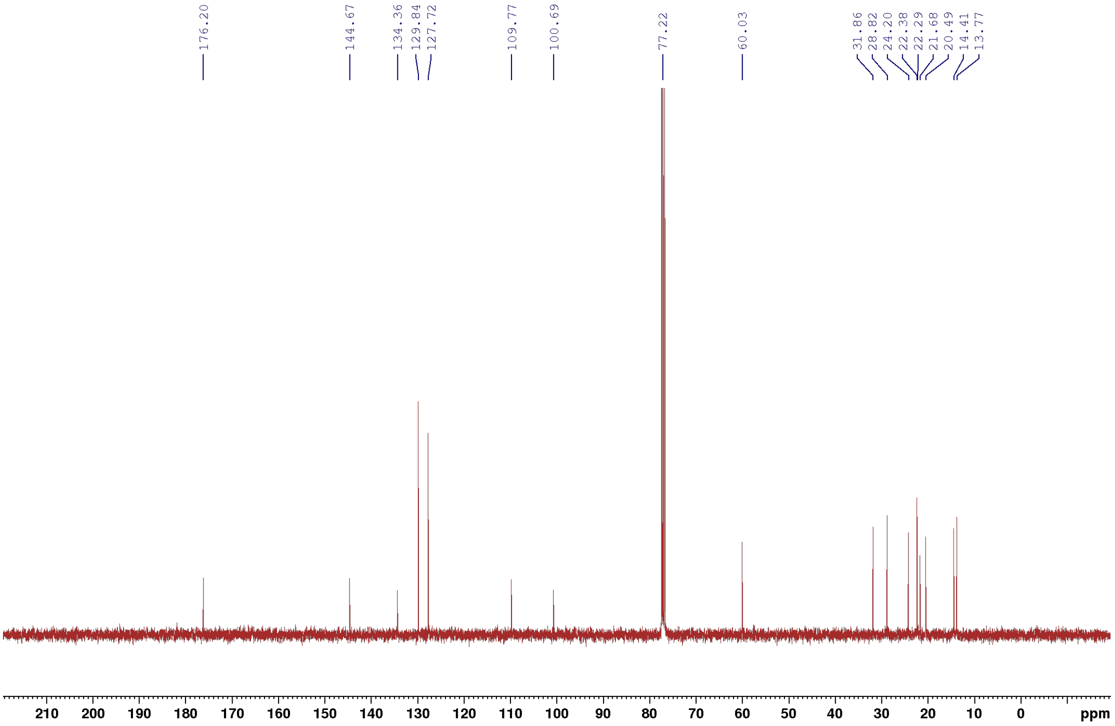

(R)-ethyl 2-ethyl-3-((R)-2-(tosyloxy)propyl)cycloprop-2-enecarboxylate (1a-2)

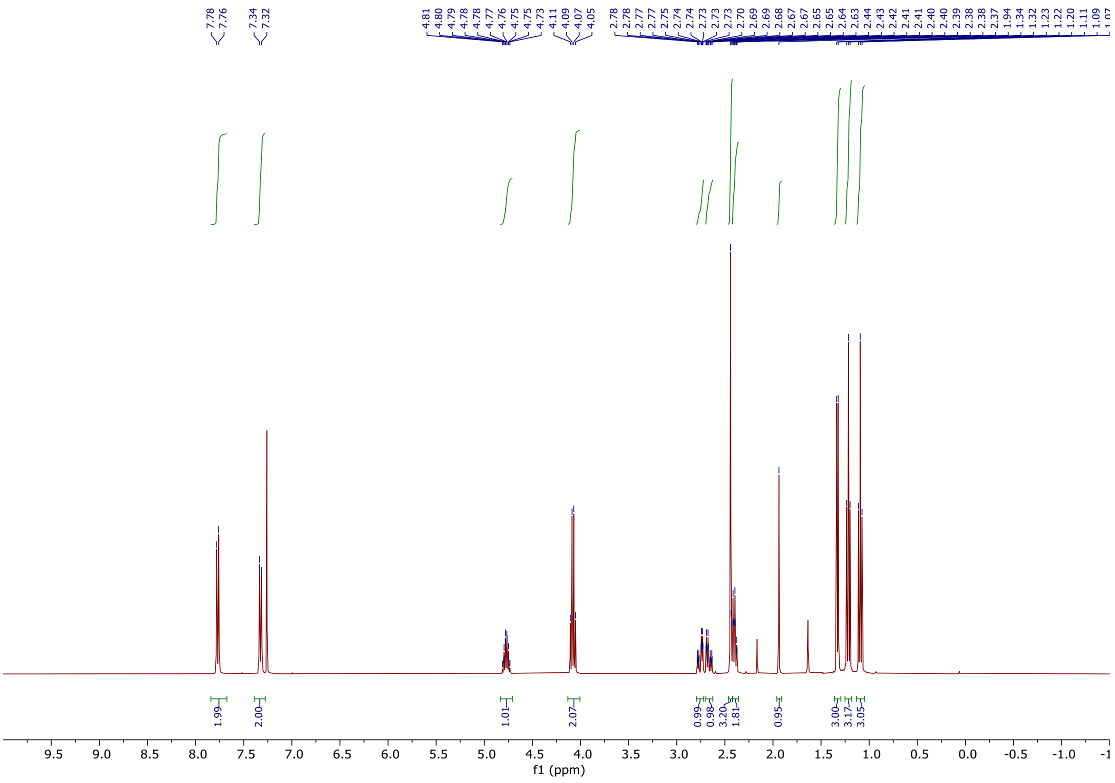

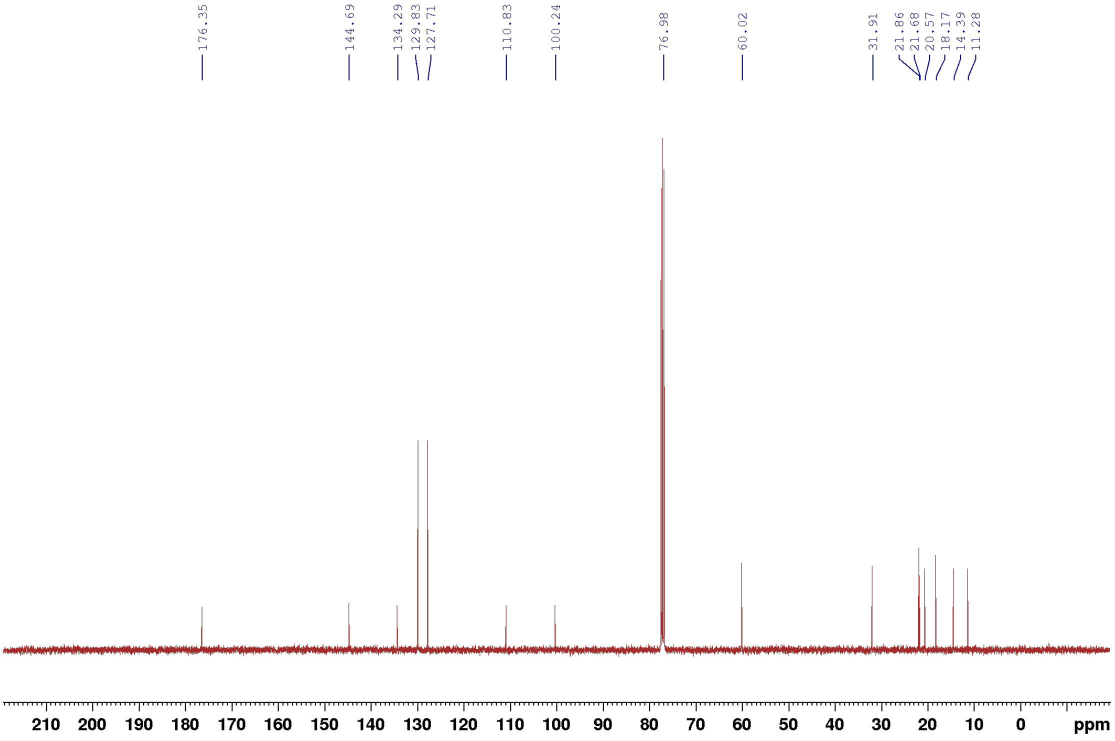

(S)-ethyl 2-cyclohexyl-3-((R)-2-(tosyloxy)propyl)cycloprop-2-enecarboxylate (1a-3)

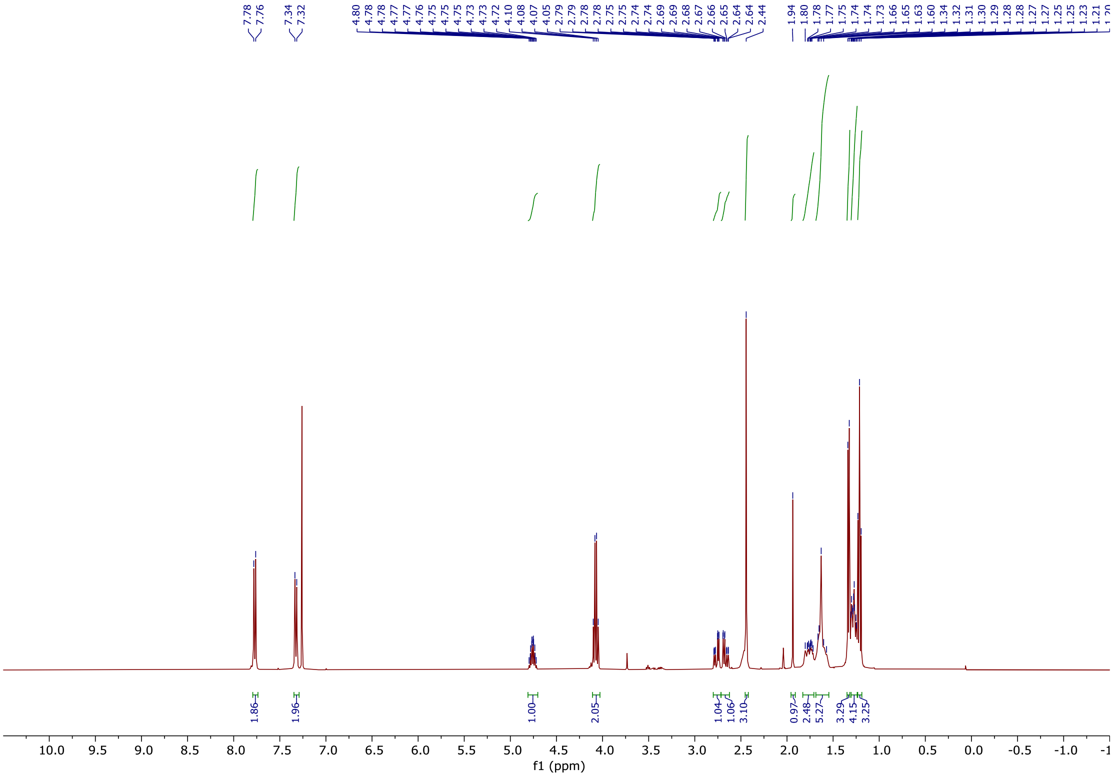

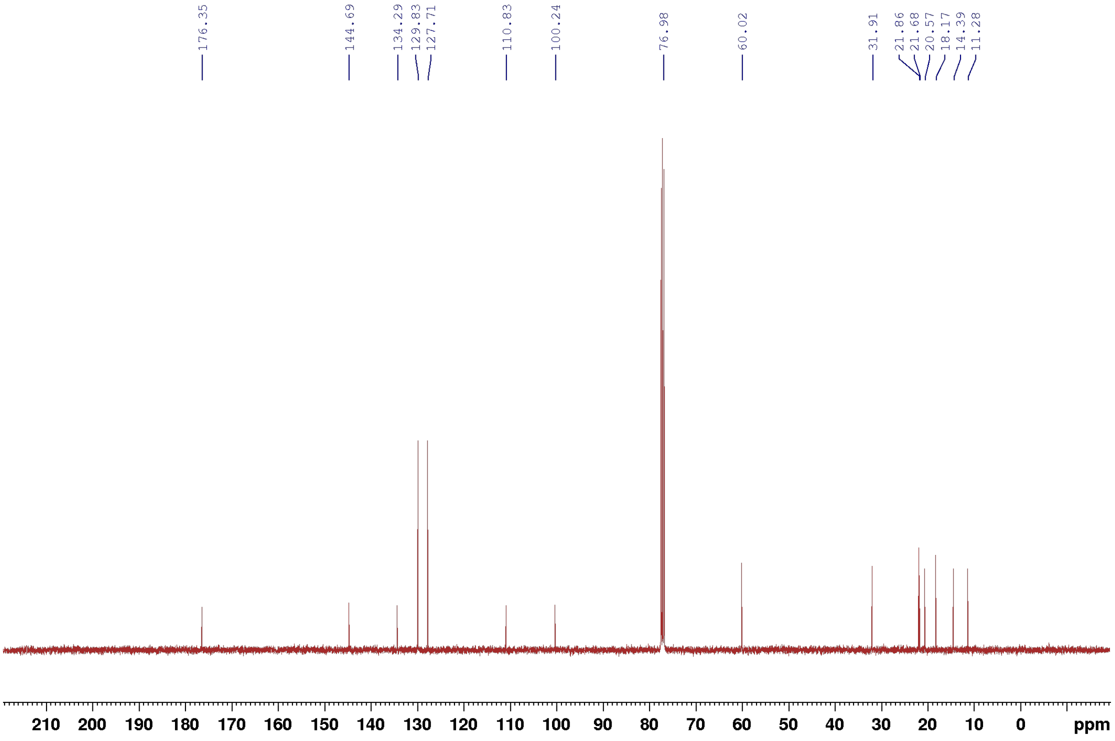

(R)-ethyl 2-phenethyl-3-((R)-2-(tosyloxy)propyl)cycloprop-2-enecarboxylate (1a-4)

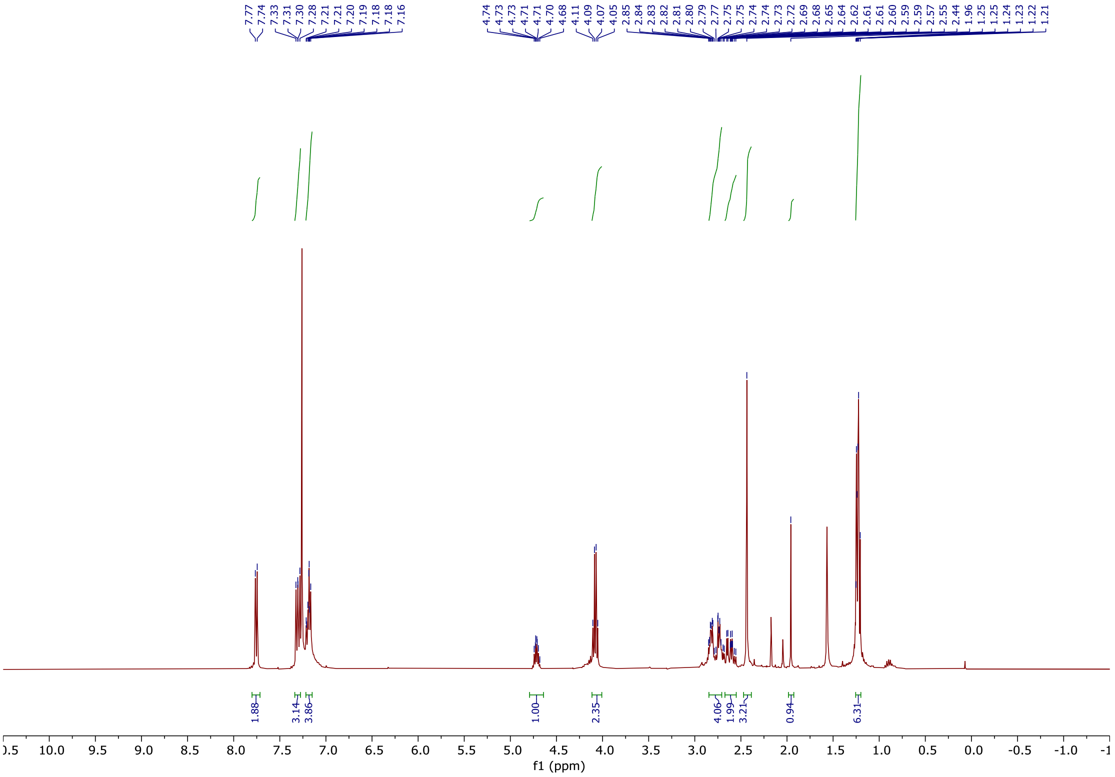

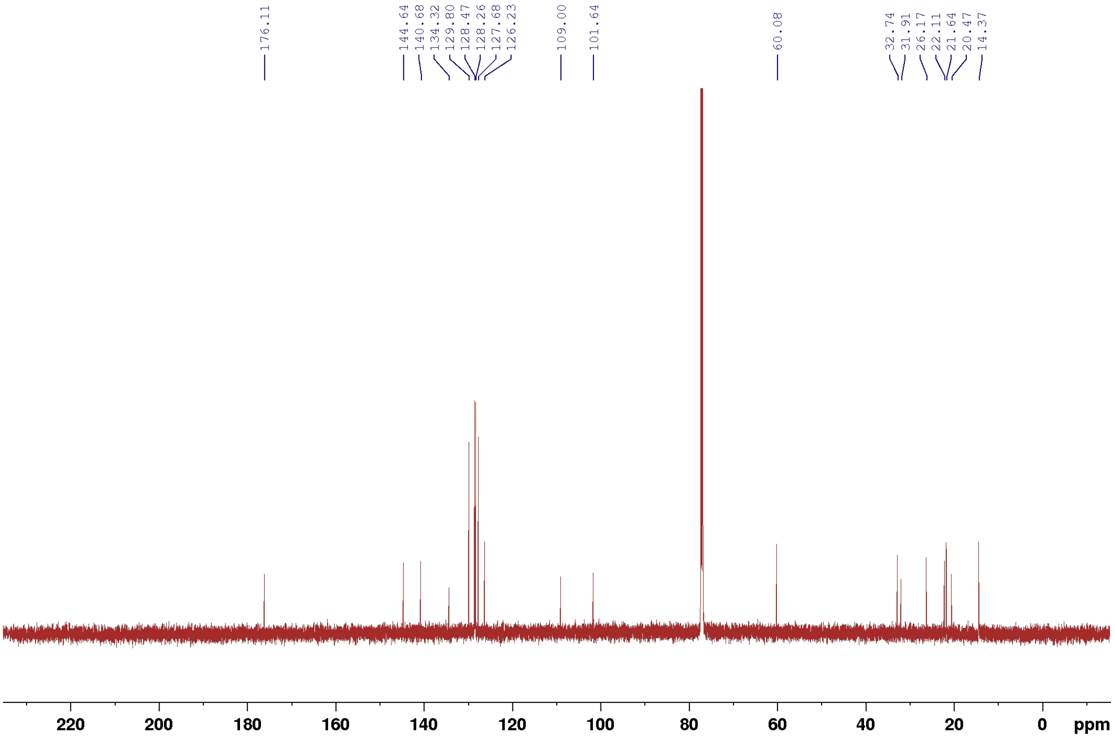

(R)-ethyl 2-(2-((tert-butyldimethylsilyl)oxy)ethyl)-3-((R)-2-(tosyloxy)propyl)cycloprop-2-enecarboxylate (1a-5)

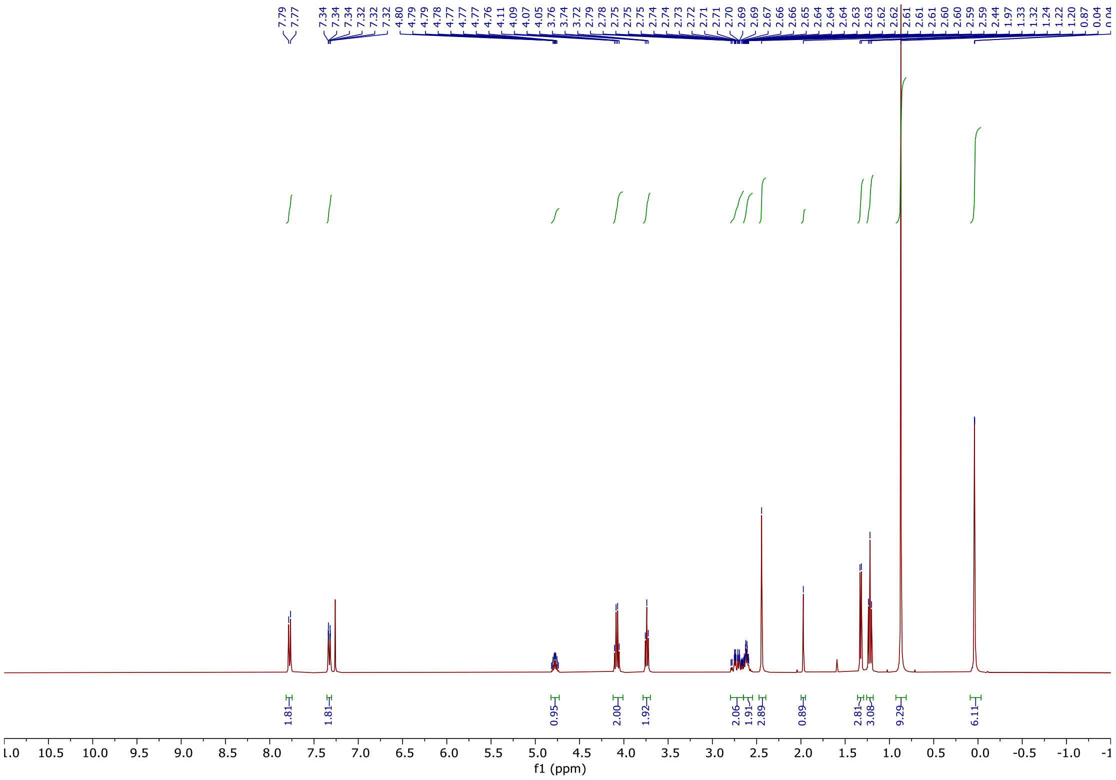

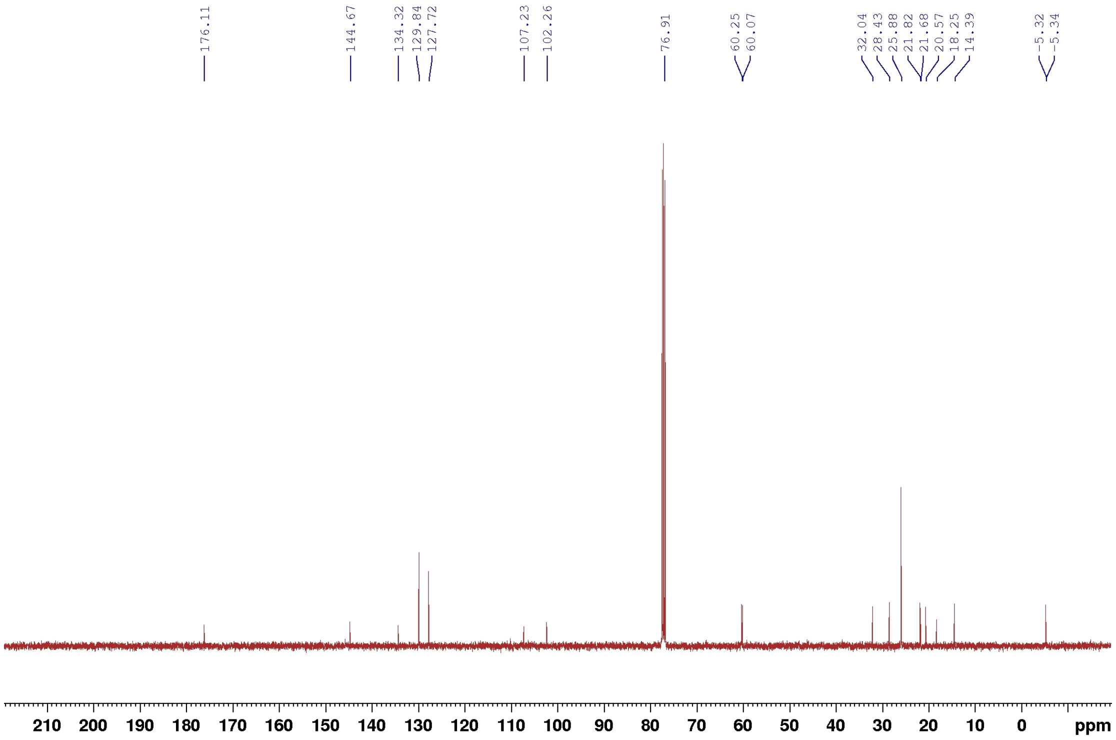

(S)-ethyl 2-(2-((tert-butyldimethylsilyl)oxy)ethyl)-3-((R)-2-(tosyloxy)propyl)cycloprop-2-enecarboxylate (1b-2)

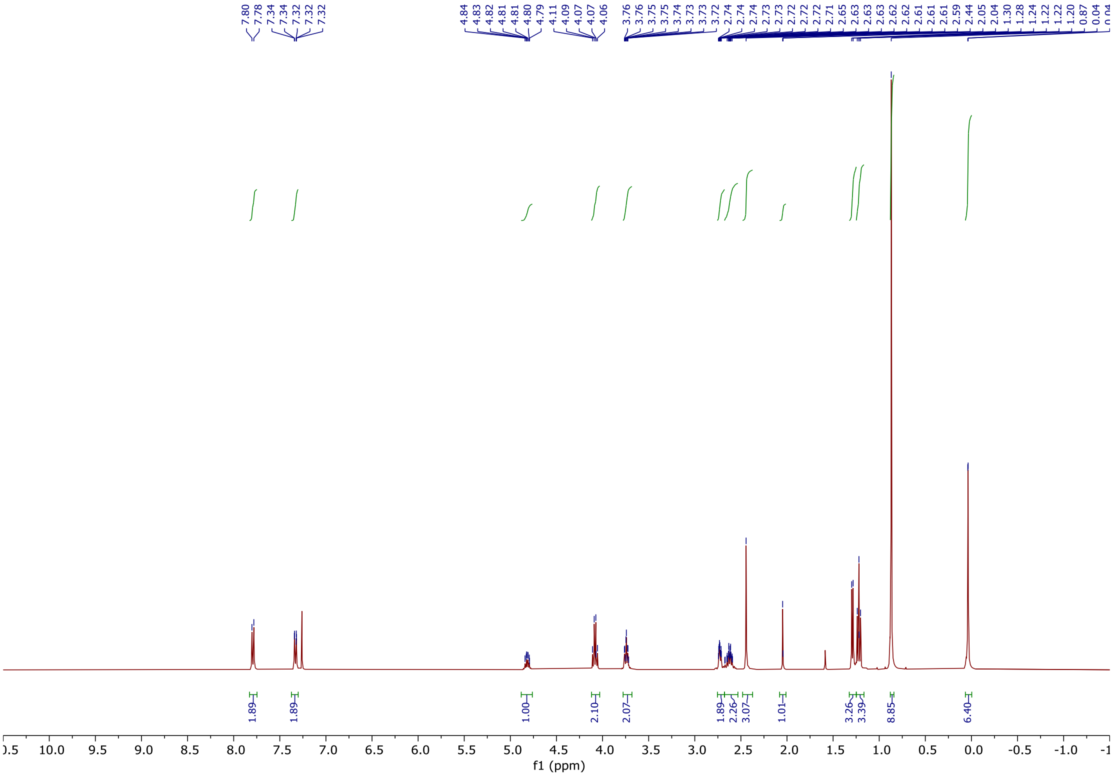

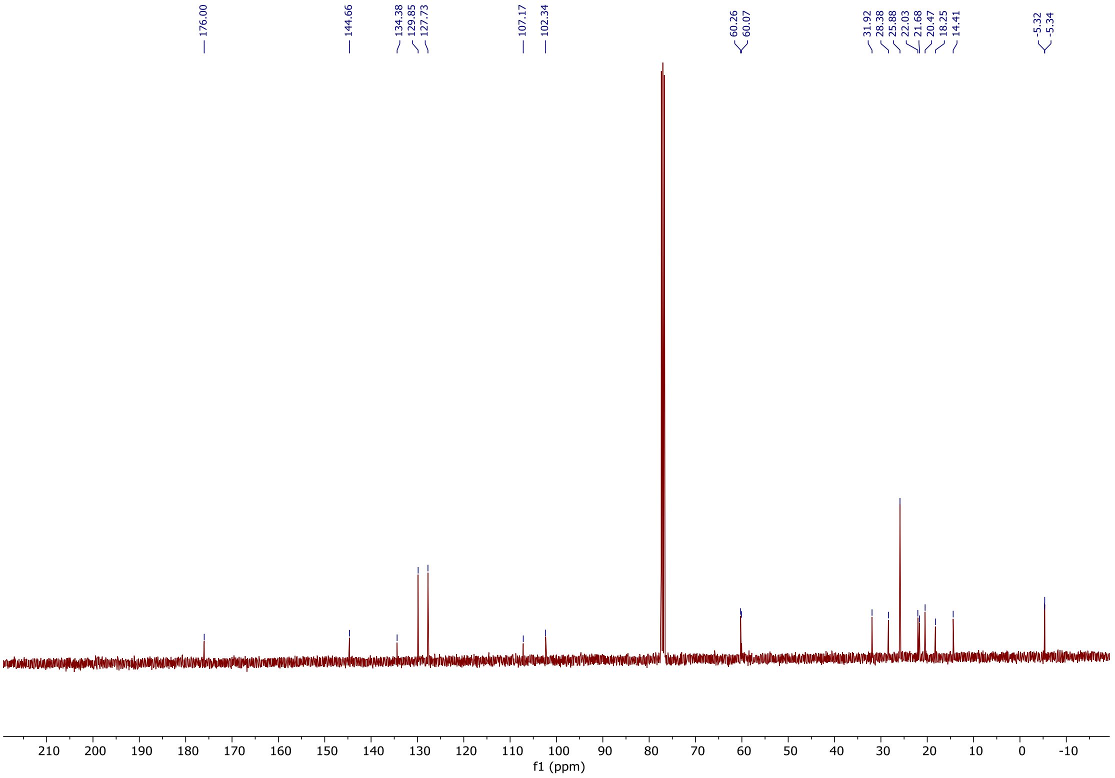

(R)-ethyl 2-butyl-3-((R)-2-(tosyloxy)butyl)cycloprop-2-enecarboxylate (1a-6)

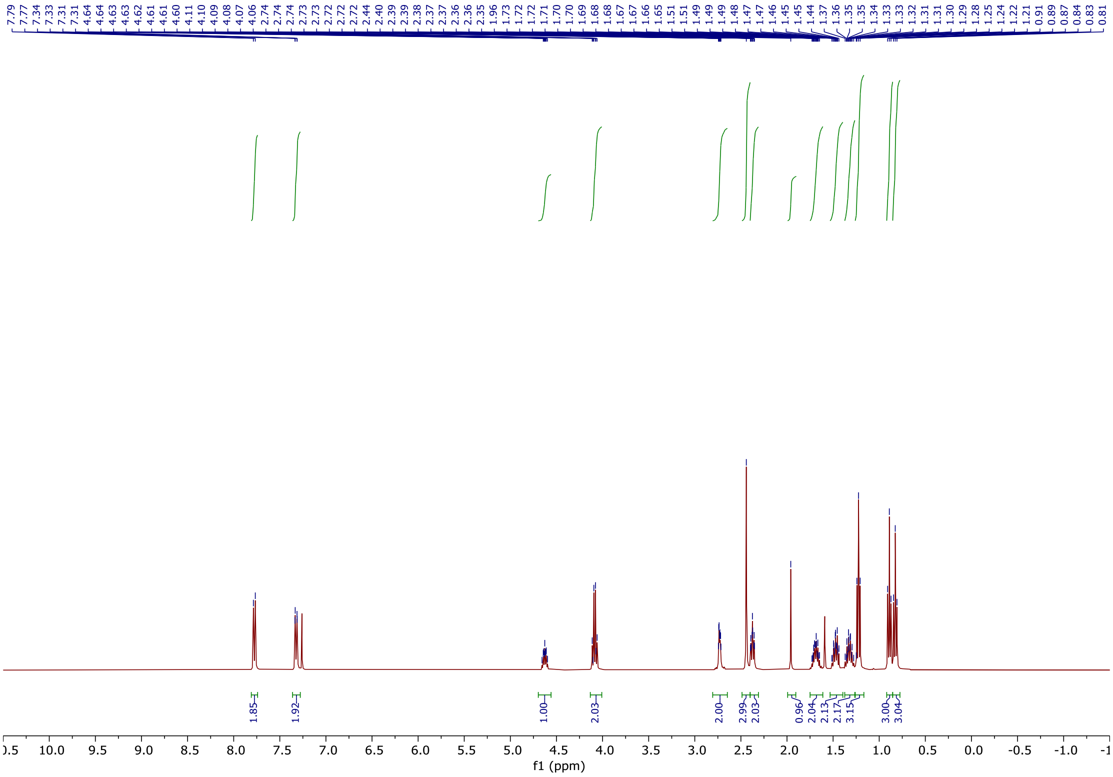

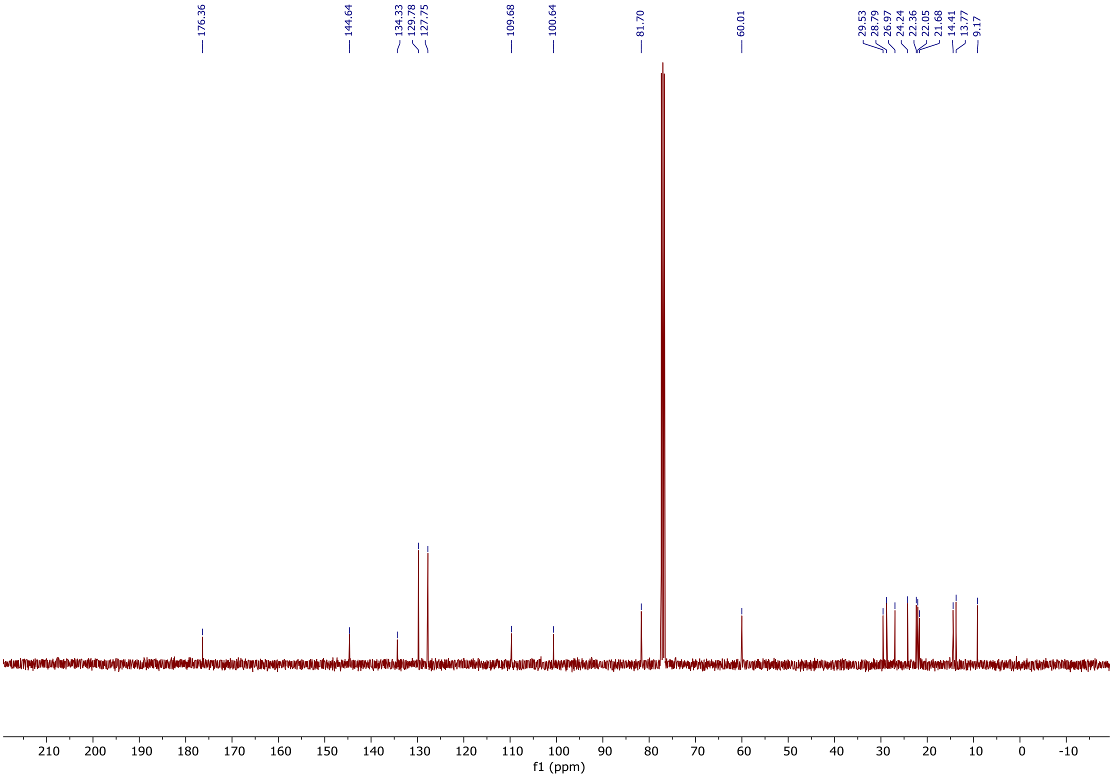

(R)-ethyl 2-butyl-3-((S)-3-methoxy-2-(tosyloxy)propyl)cycloprop-2-enecarboxylate (1a-7)

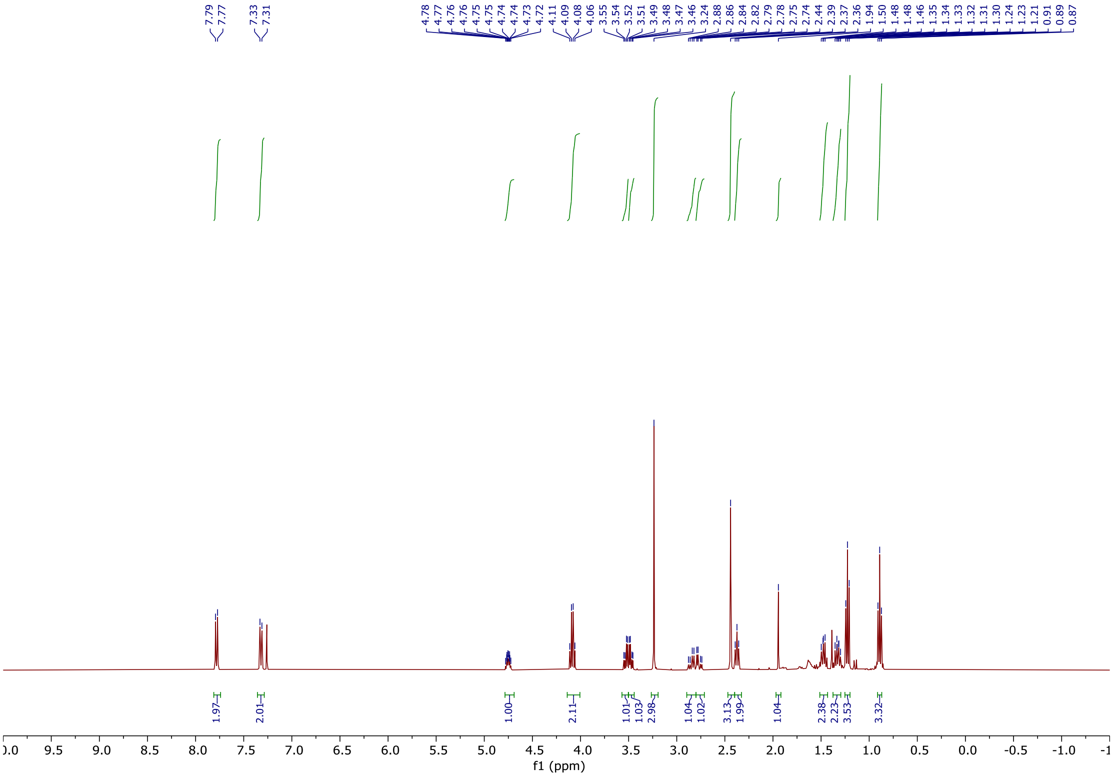

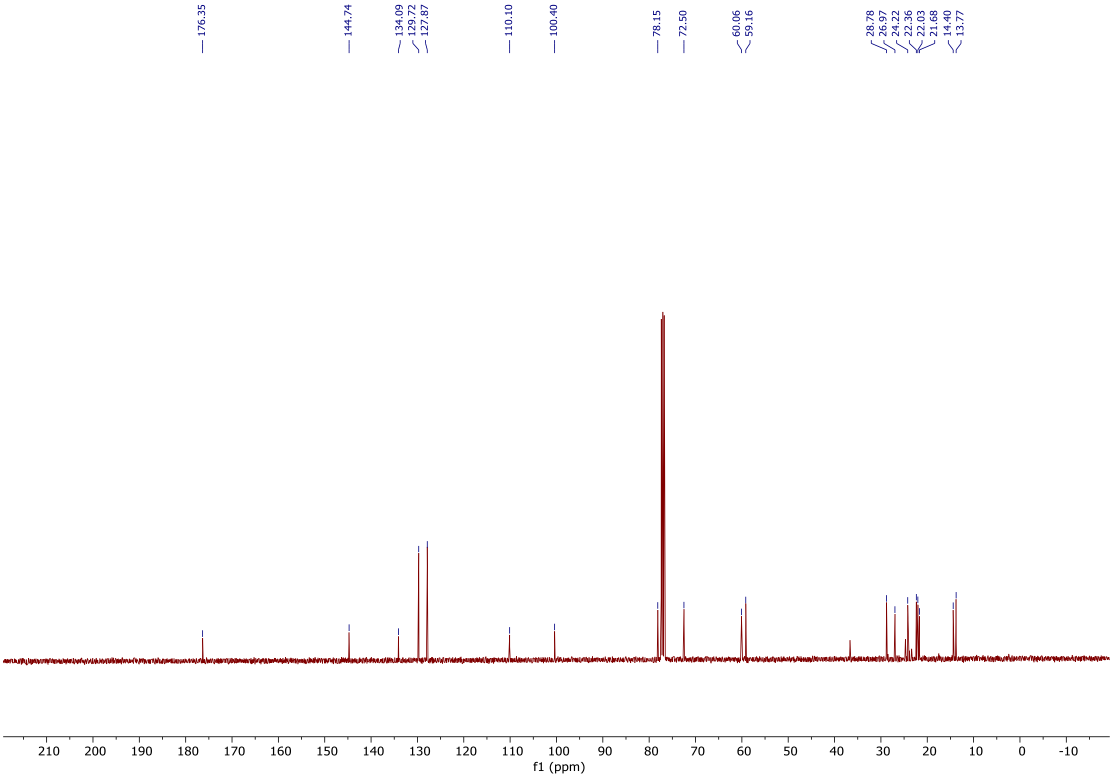

(S)-ethyl 2-phenyl-3-((R)-2-(tosyloxy)propyl)cycloprop-2-enecarboxylate (1a-8)

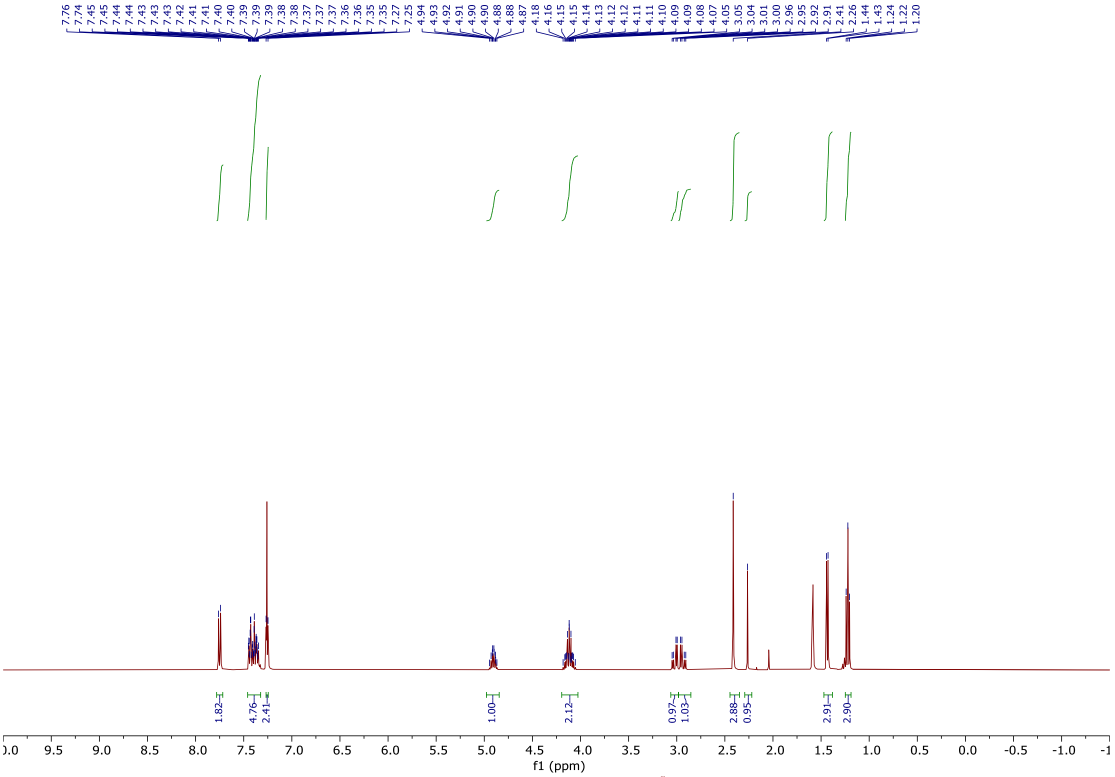

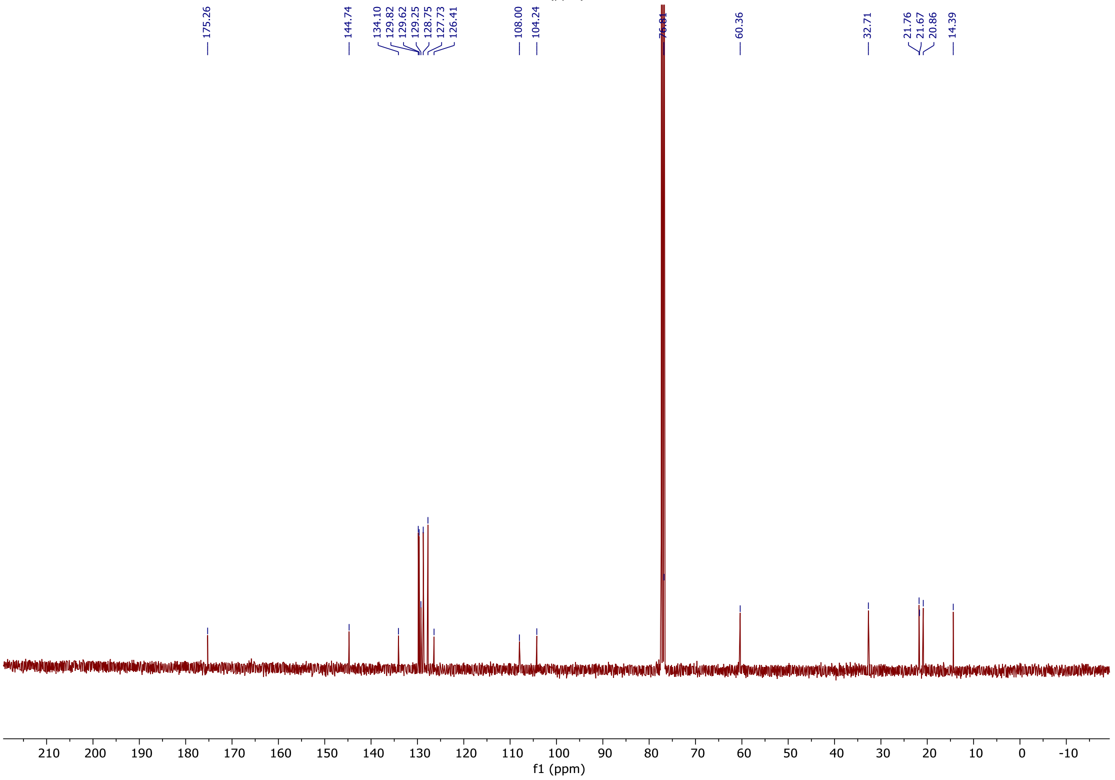

(R)-ethyl 2-butyl-3-((2S,3R)-3-(tosyloxy)butan-2-yl)cycloprop-2-enecarboxylate (1a-9)

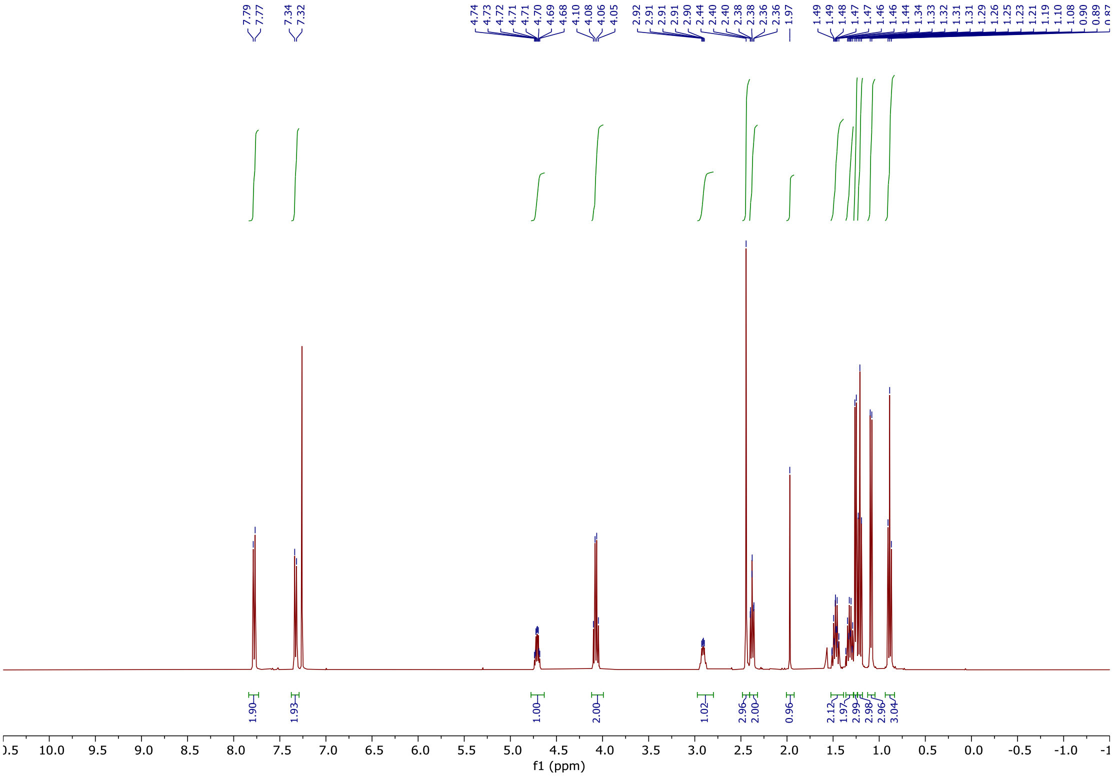

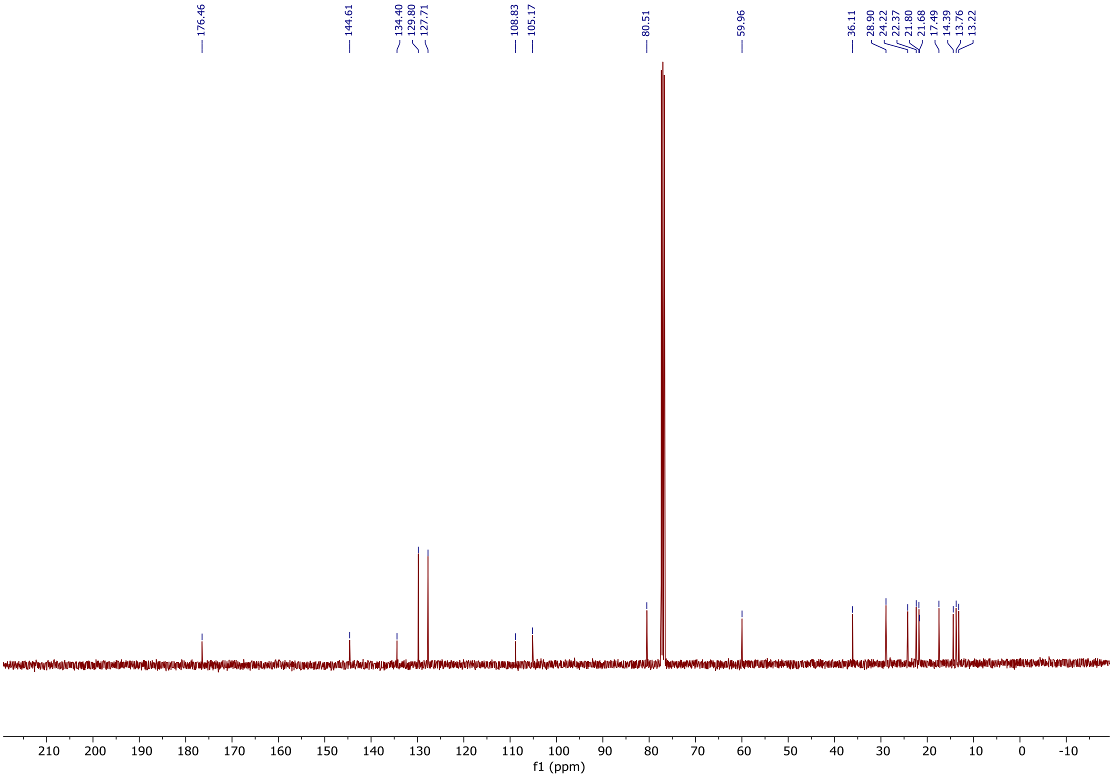

(R)-ethyl 2-butyl-1-methyl-3-((R)-2-(tosyloxy)propyl)cycloprop-2-enecarboxylate (1a-10)

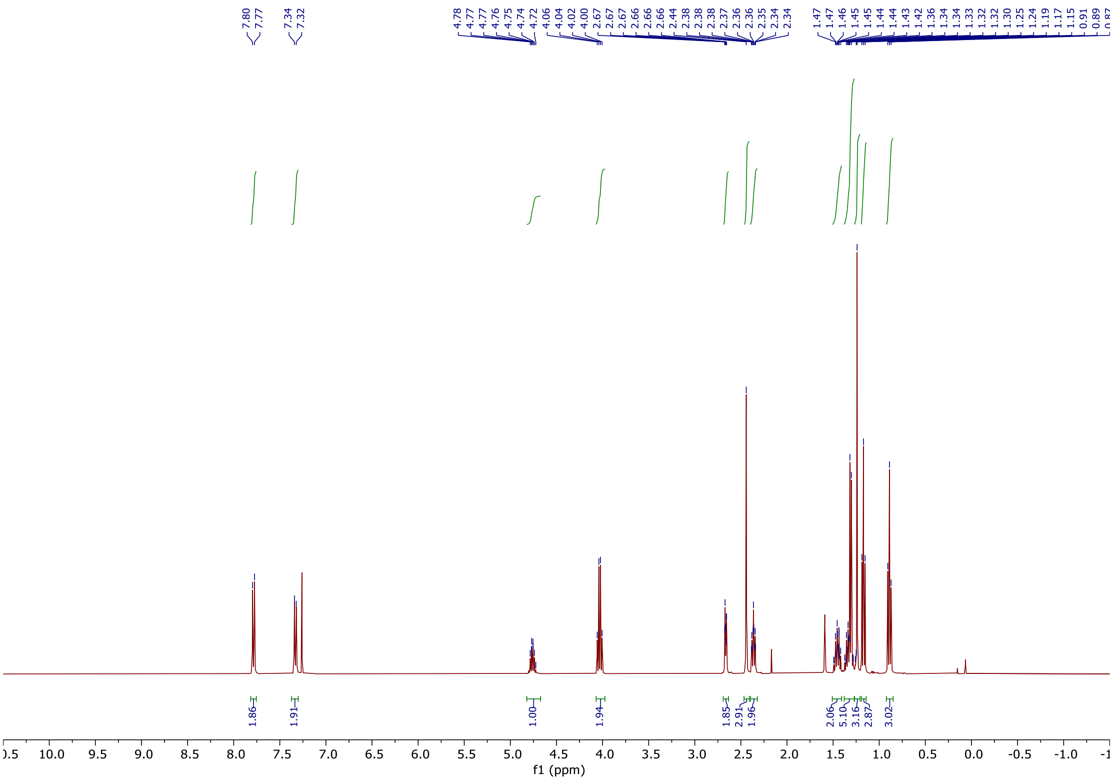

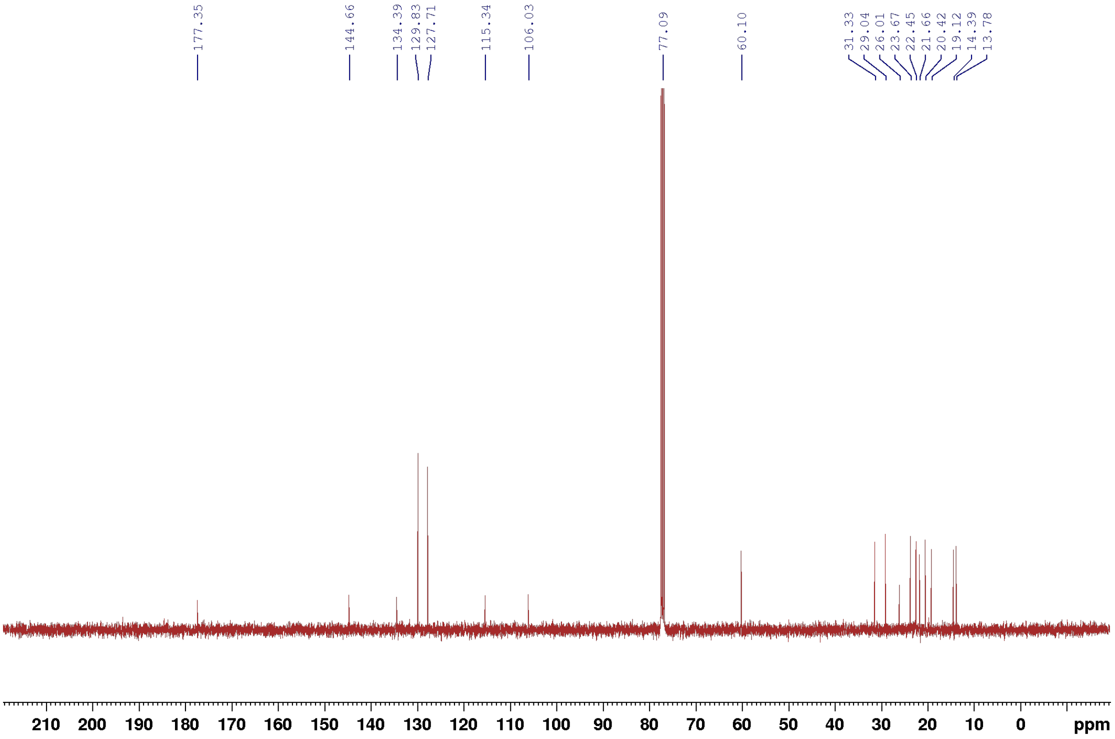

(R)-ethyl 2-butyl-3-((S)-2-chloropropyl)-1-methylcycloprop-2-enecarboxylate (6a-1)

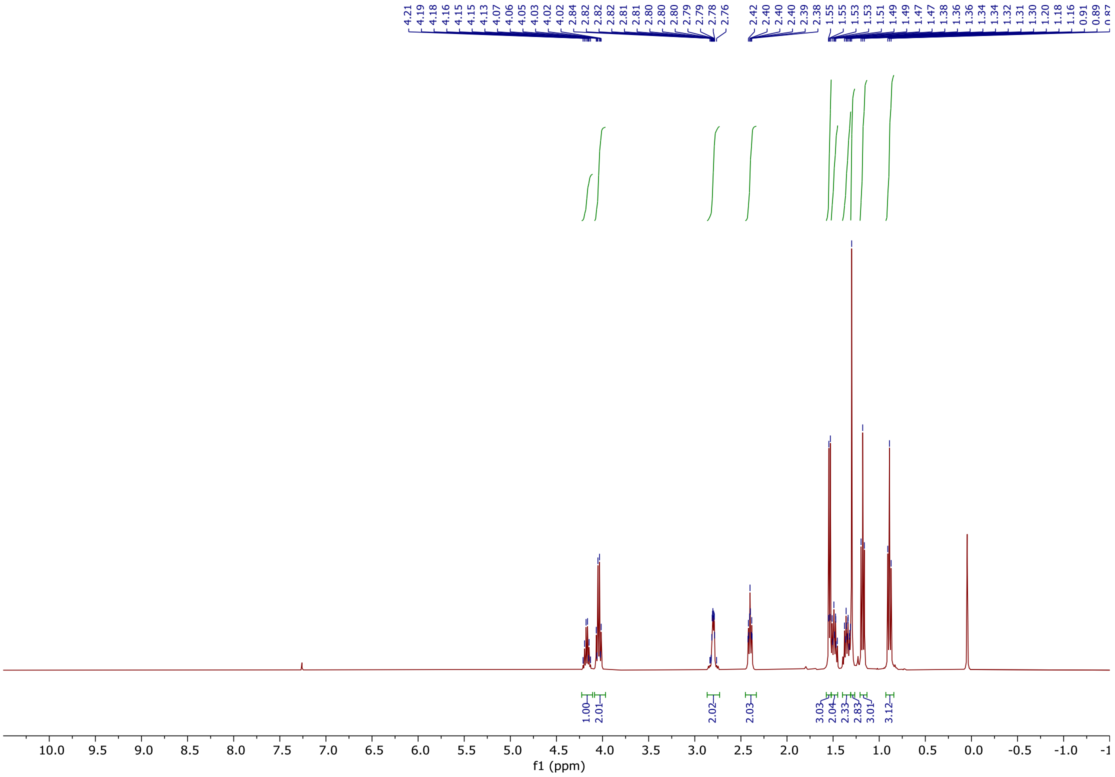

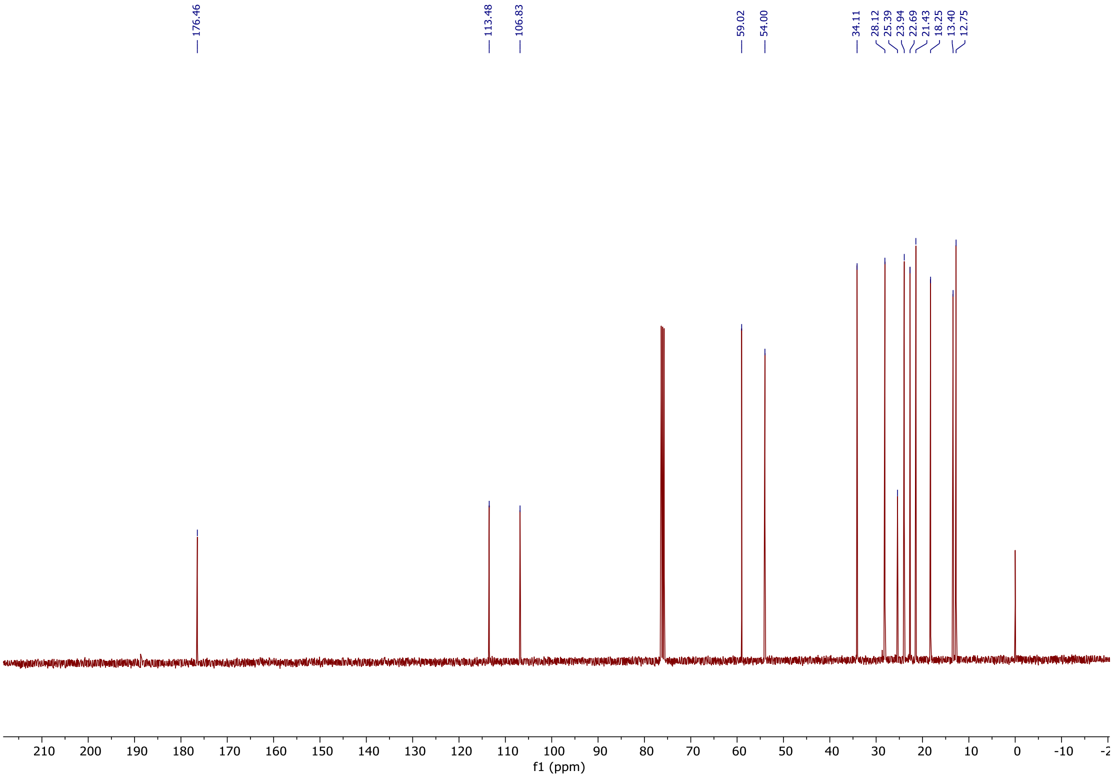

(S)-ethyl 2-butyl-3-((R)-2-chloropropyl)cycloprop-2-enecarboxylate (6b-1)

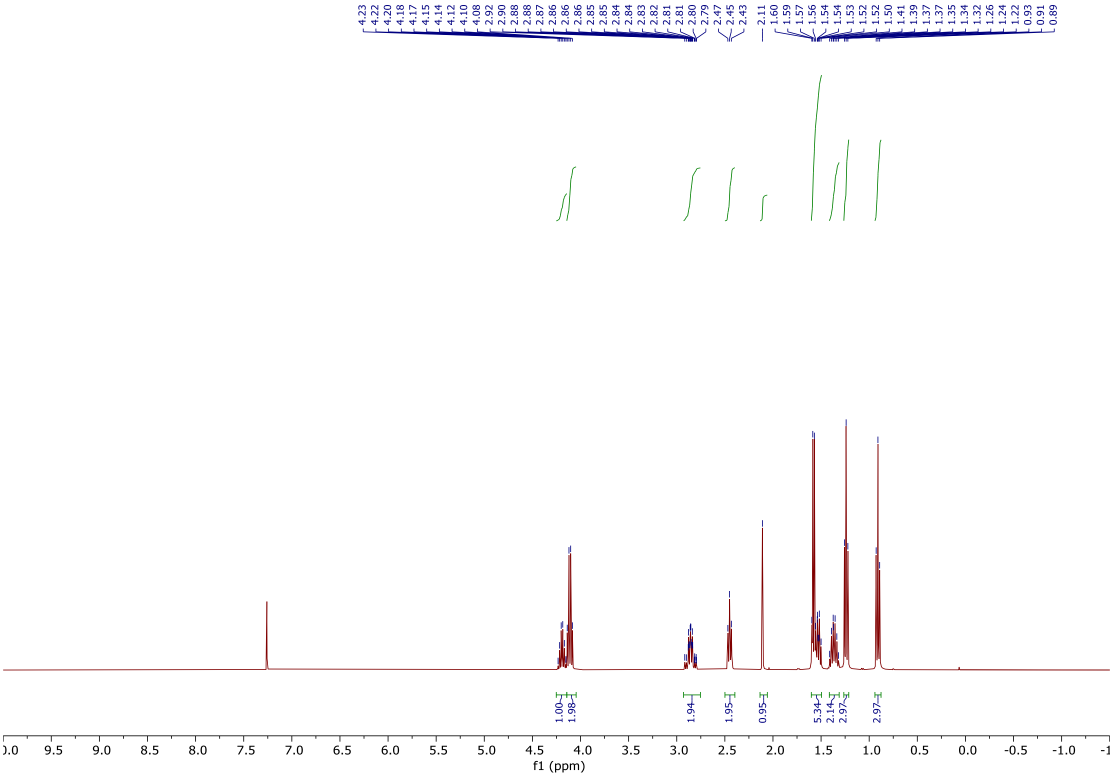

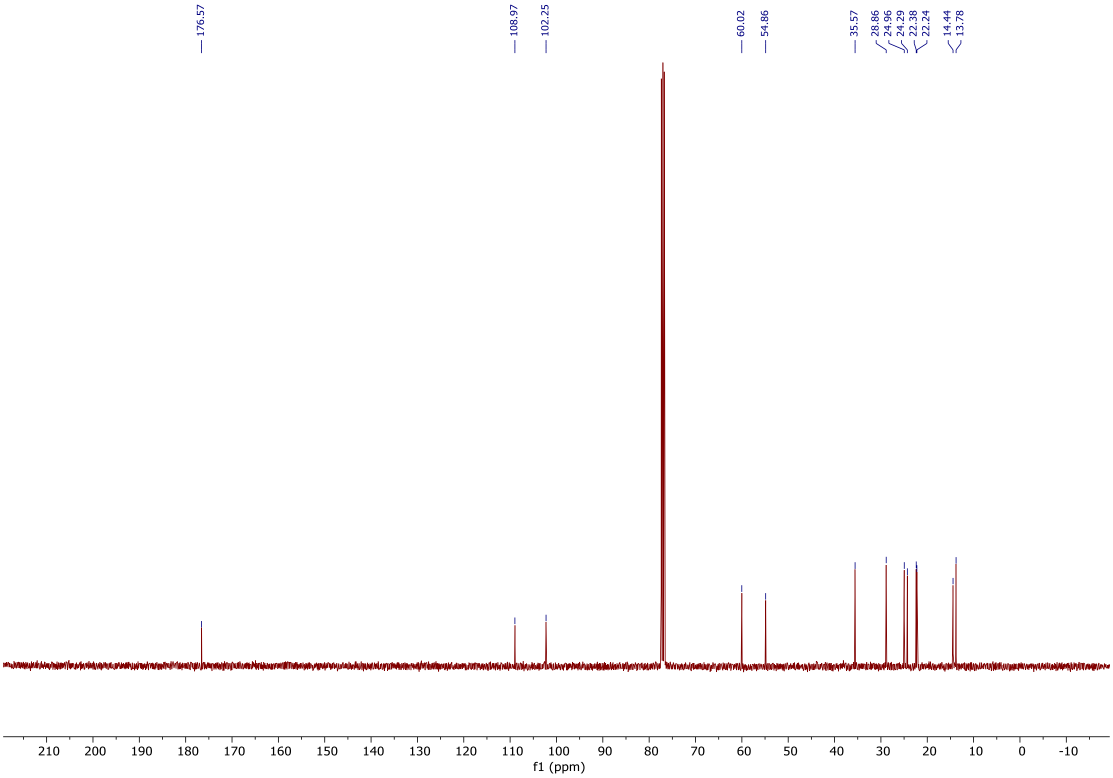

((S)-2-butyl-3-((R)-2-chloropropyl)cycloprop-2-en-1-yl)methanol (7b-1)

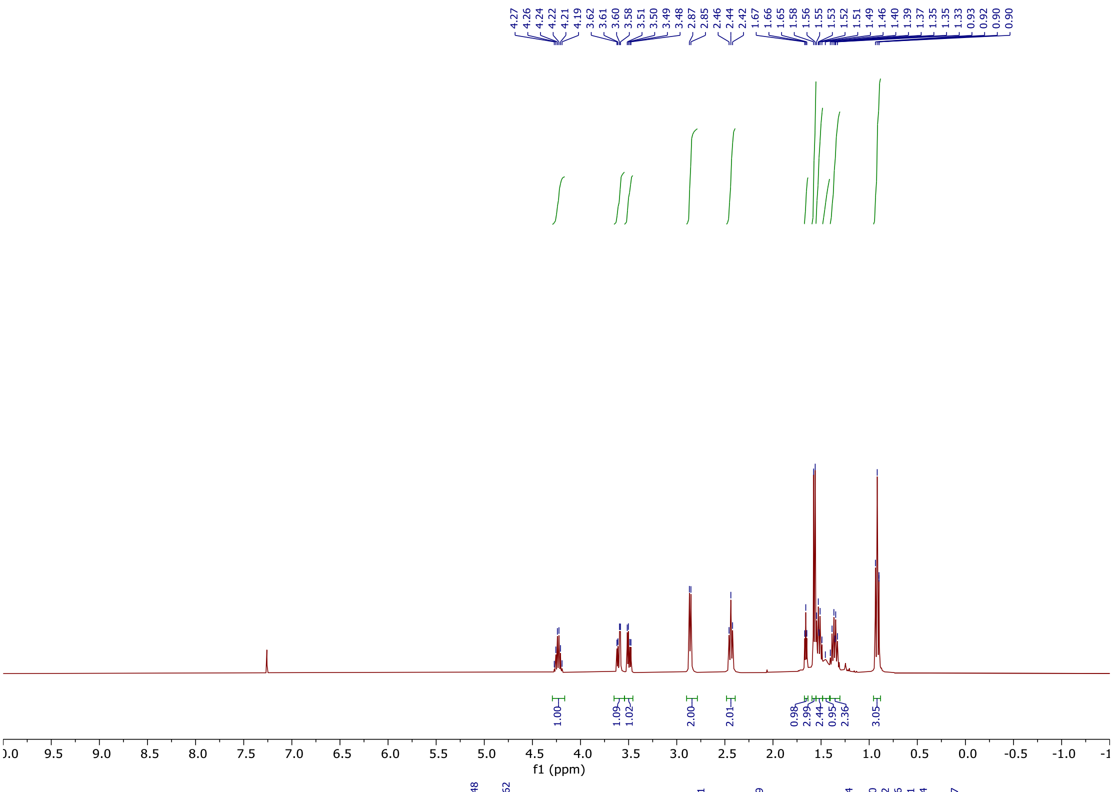

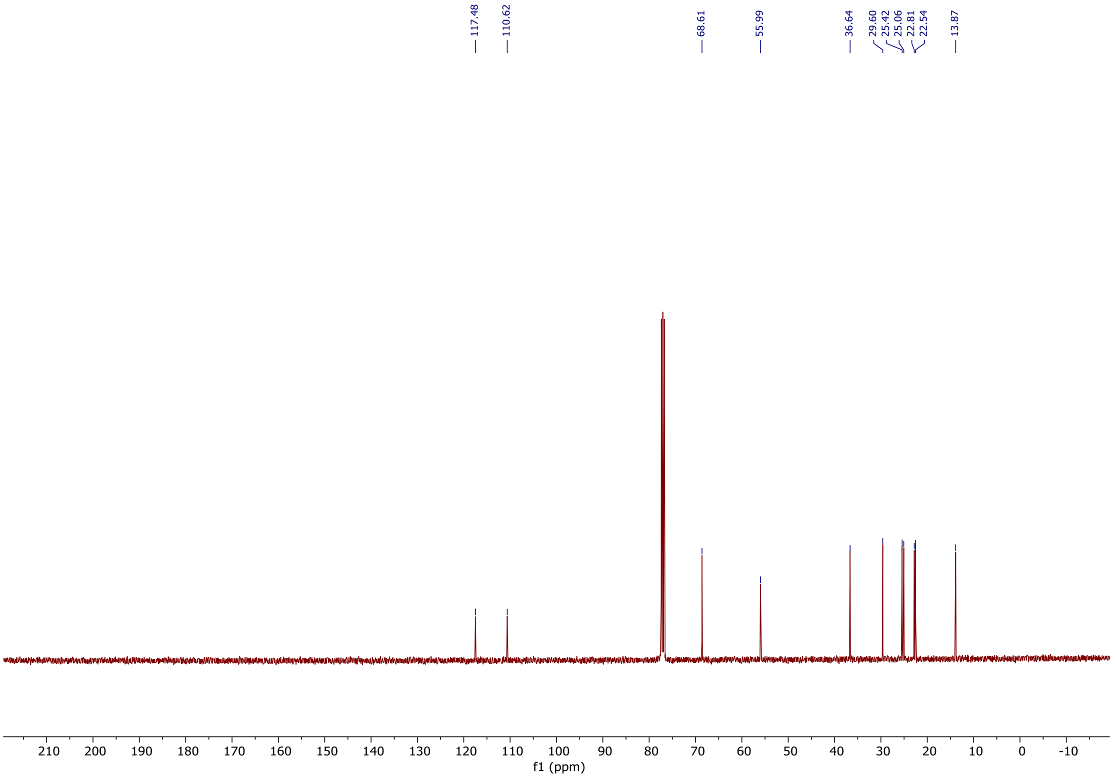

(R)-1-((R)-2-butyl-3-(hydroxymethyl)cycloprop-1-en-1-yl)propan-2-yl 4-methylbenzenesulfonate (7a-1)

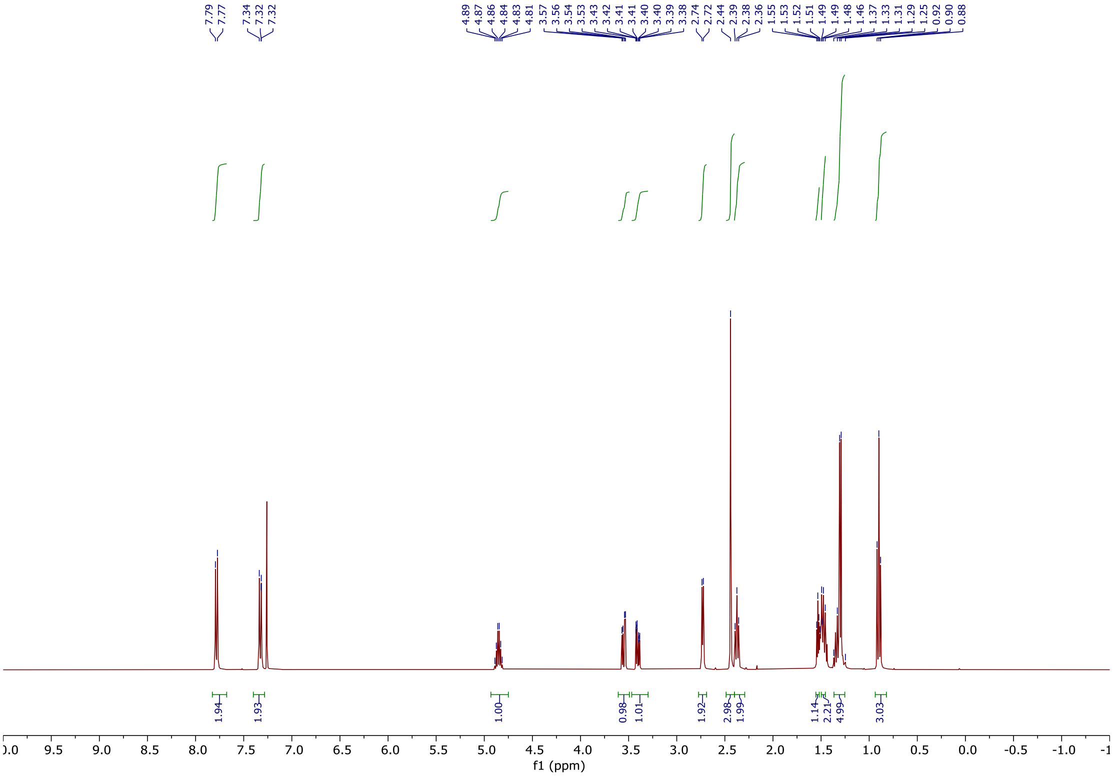

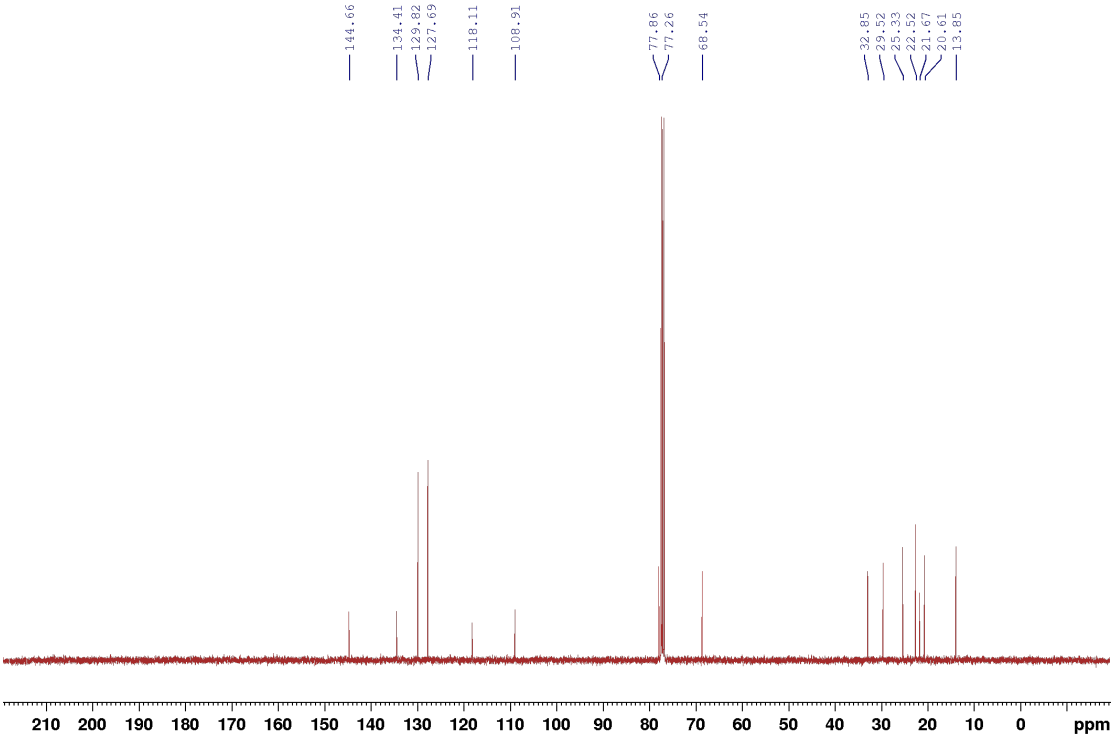

17. NMR spectra of spiropentanes

(1R,2S,3R,4S)-ethyl 2-butyl-2-hexyl-4-methylspiro[2.2]pentane-1-carboxylate (3a-1)

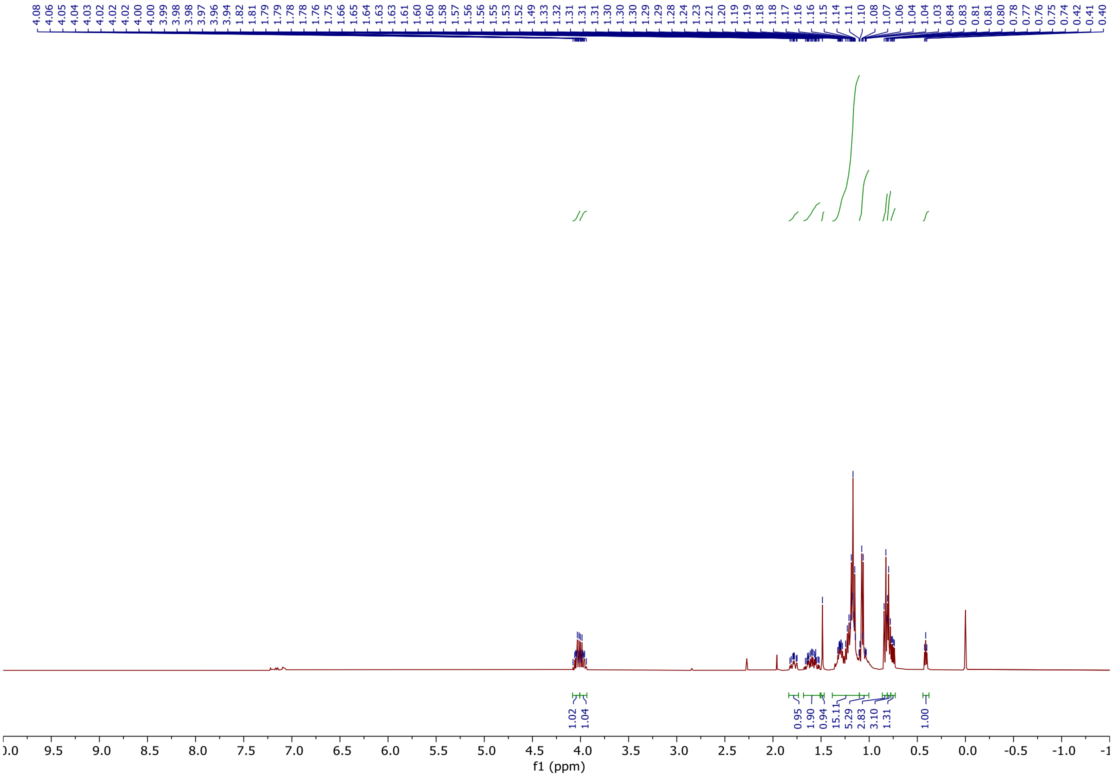

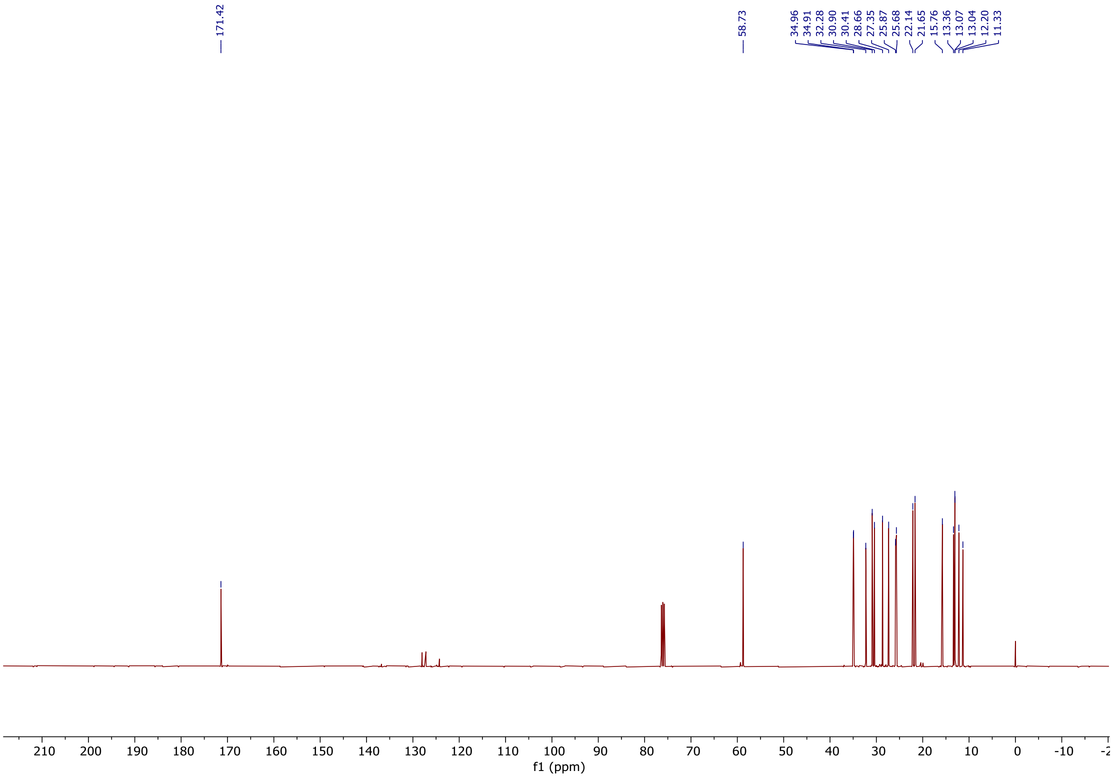

(1R,2R,3R,4S)-ethyl 2-butyl-2-ethyl-4-methylspiro[2.2]pentane-1-carboxylate (3a-2)

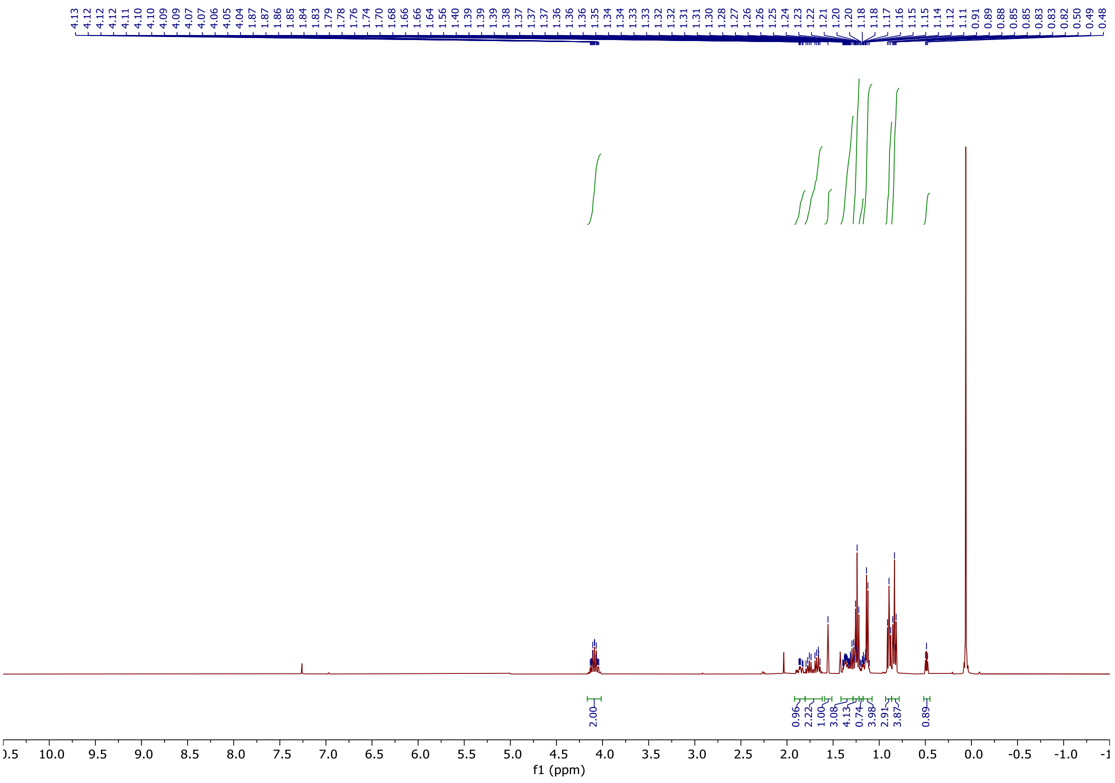

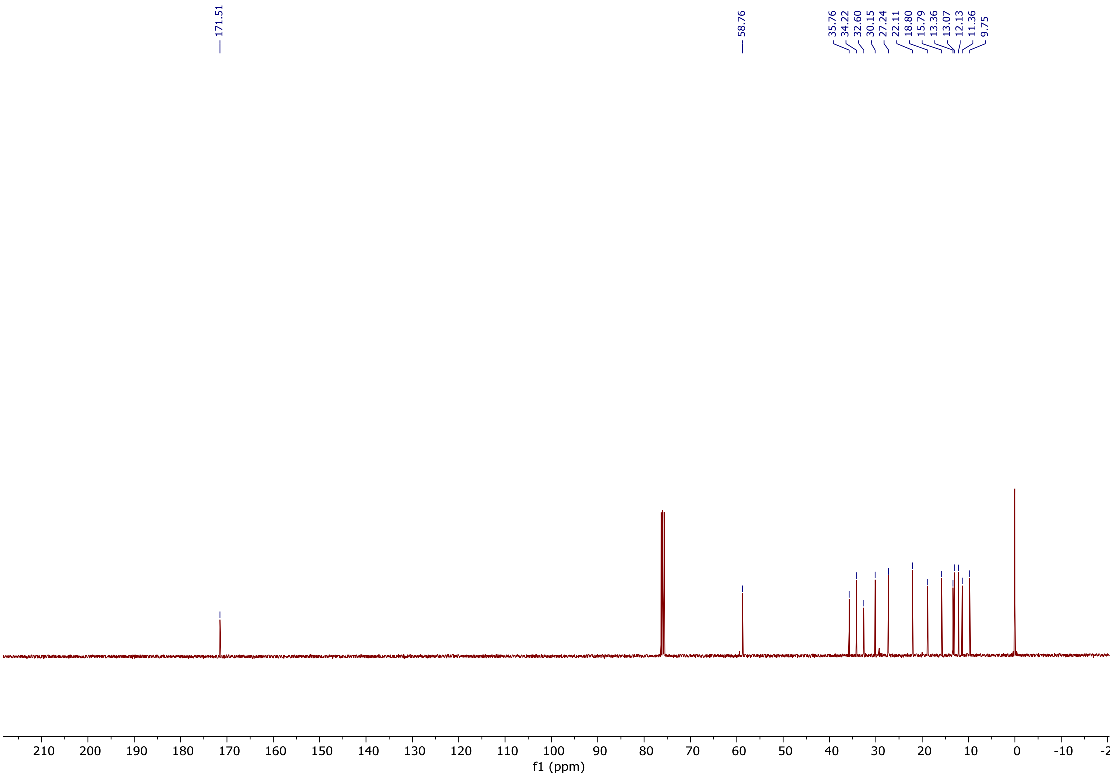

(1R,2S,3R,4S)-ethyl 2-butyl-2-ethyl-4-methylspiro[2.2]pentane-1-carboxylate (3a-3)

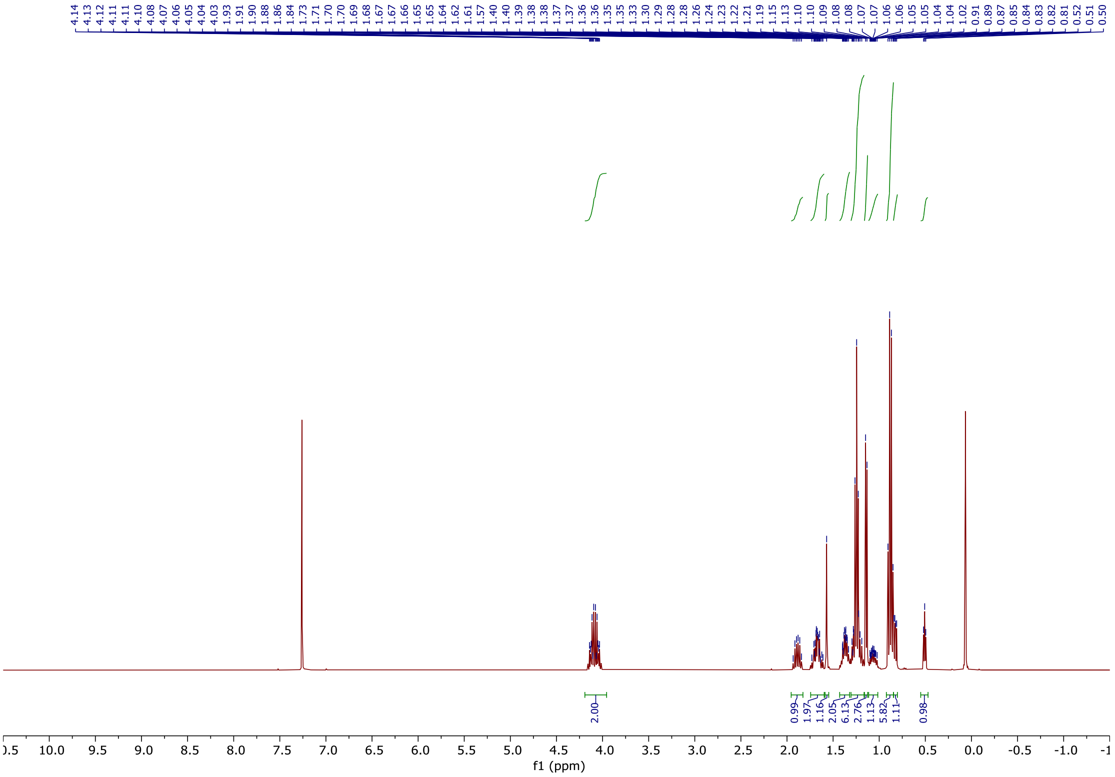

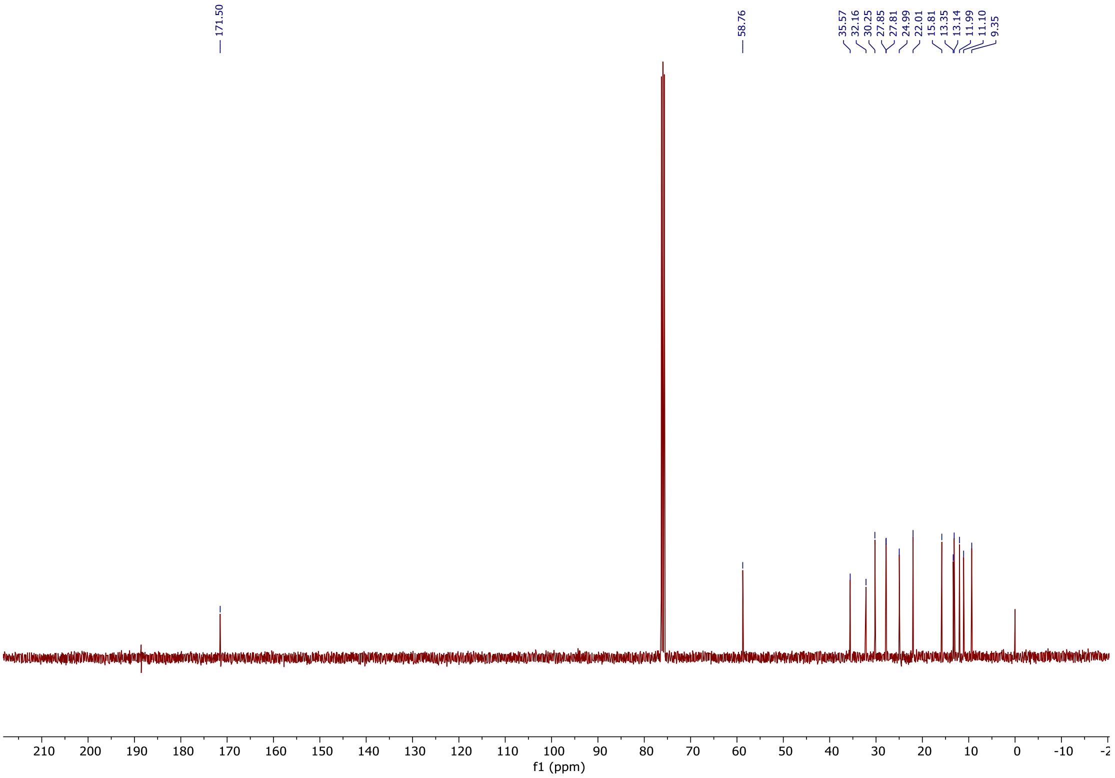

(1R,2S,3R,4S)-ethyl 2-butyl-2-isopropyl-4-methylspiro[2.2]pentane-1-carboxylate (3a-4)

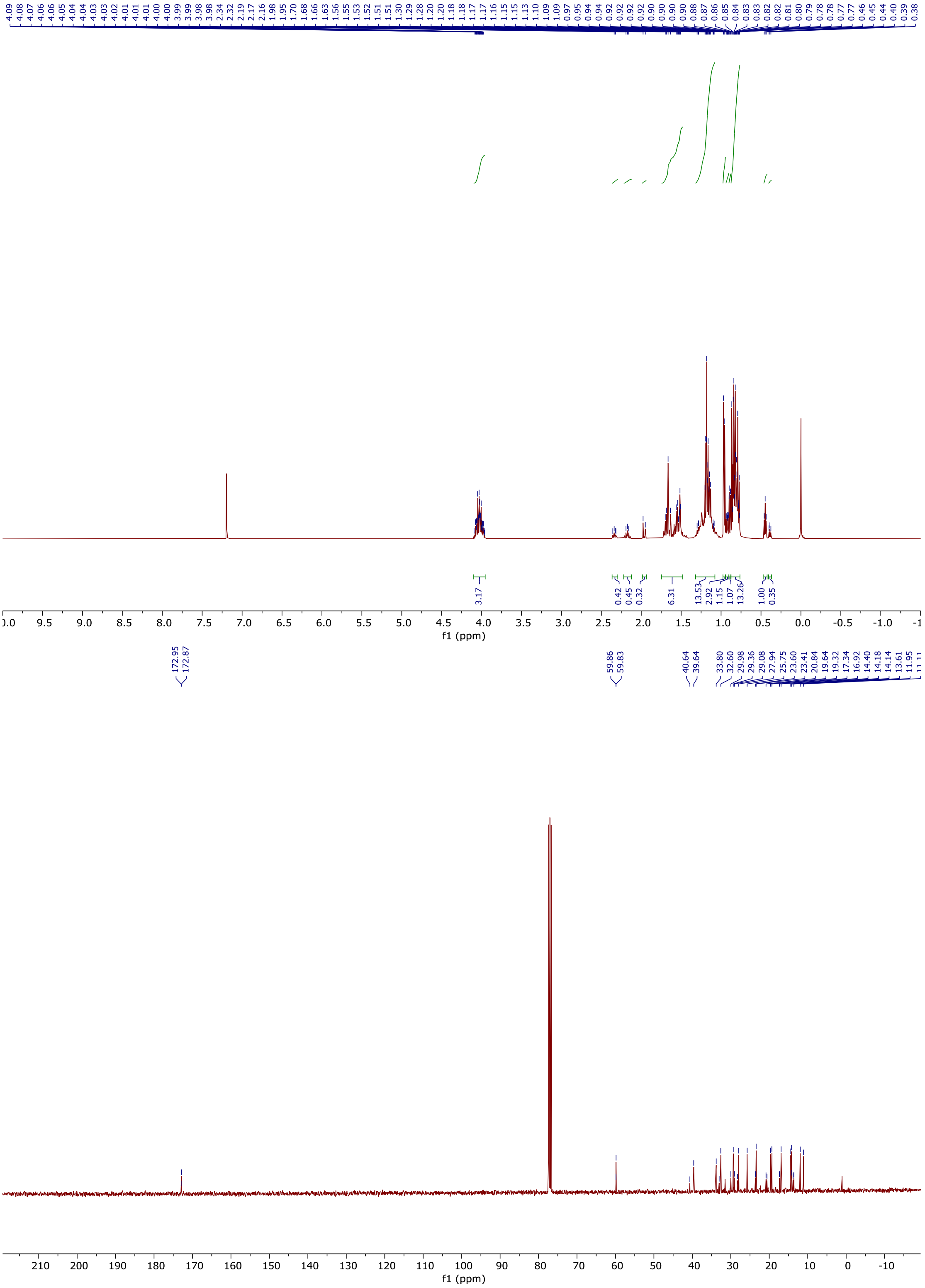

(1R,2R,3R,4S)-ethyl 2-ethyl-4-methyl-2-phenethylspiro[2.2]pentane-1-carboxylate (3a-5)

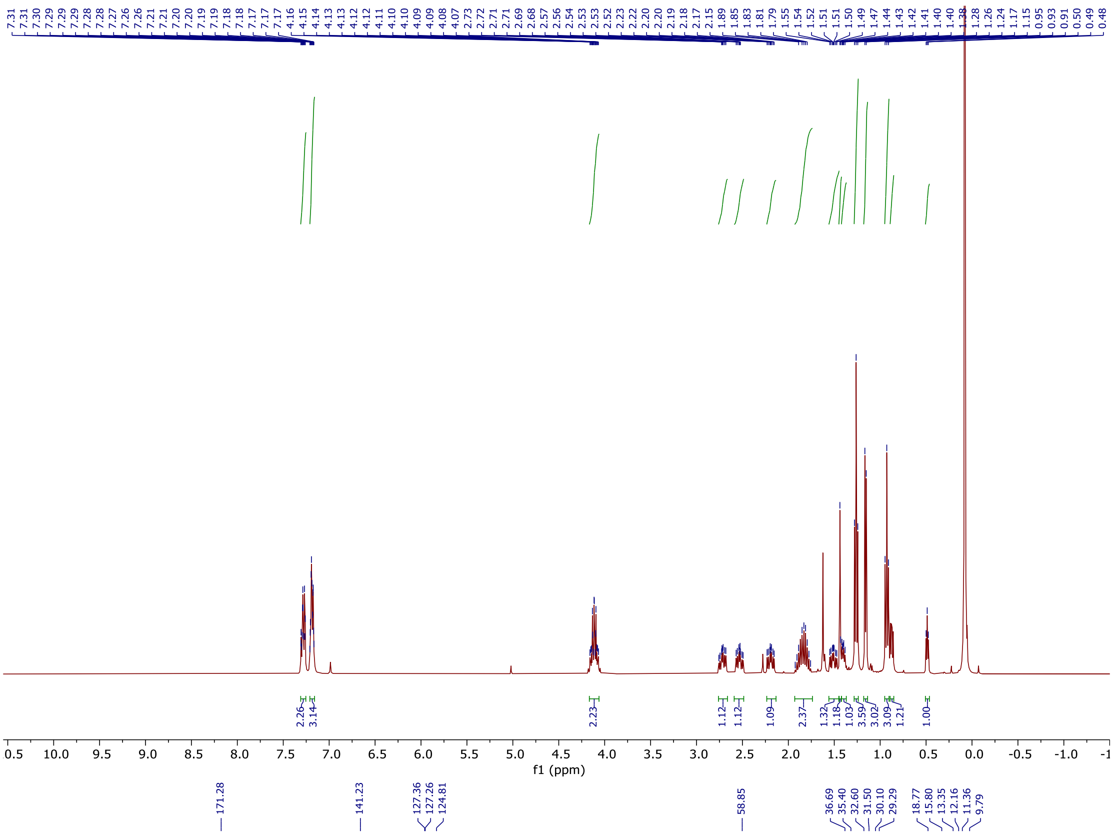

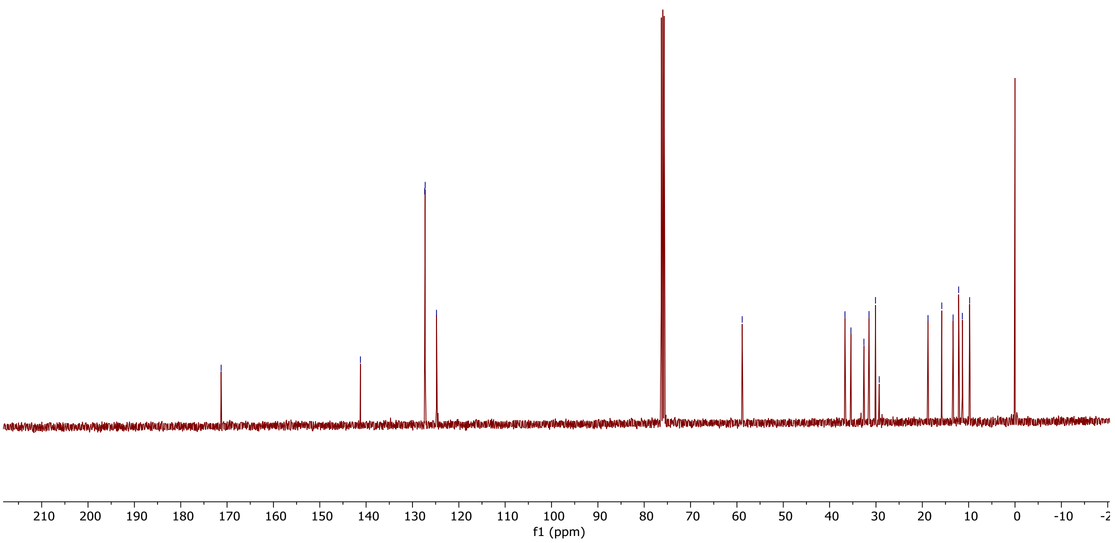

(1R,2R,3R,4S)-ethyl 2-butyl-2-(2-((tert-butyldimethylsilyl)oxy)ethyl)-4-methylspiro [2.2]pentane-1-carboxylate (3a-6)

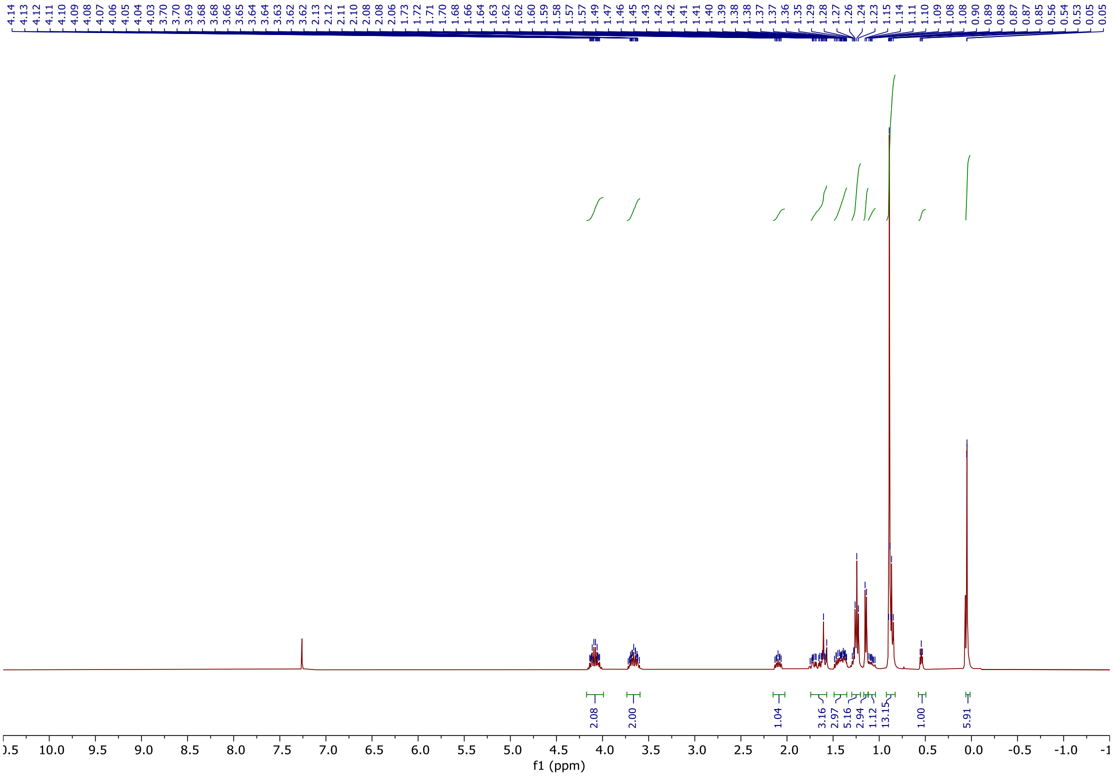

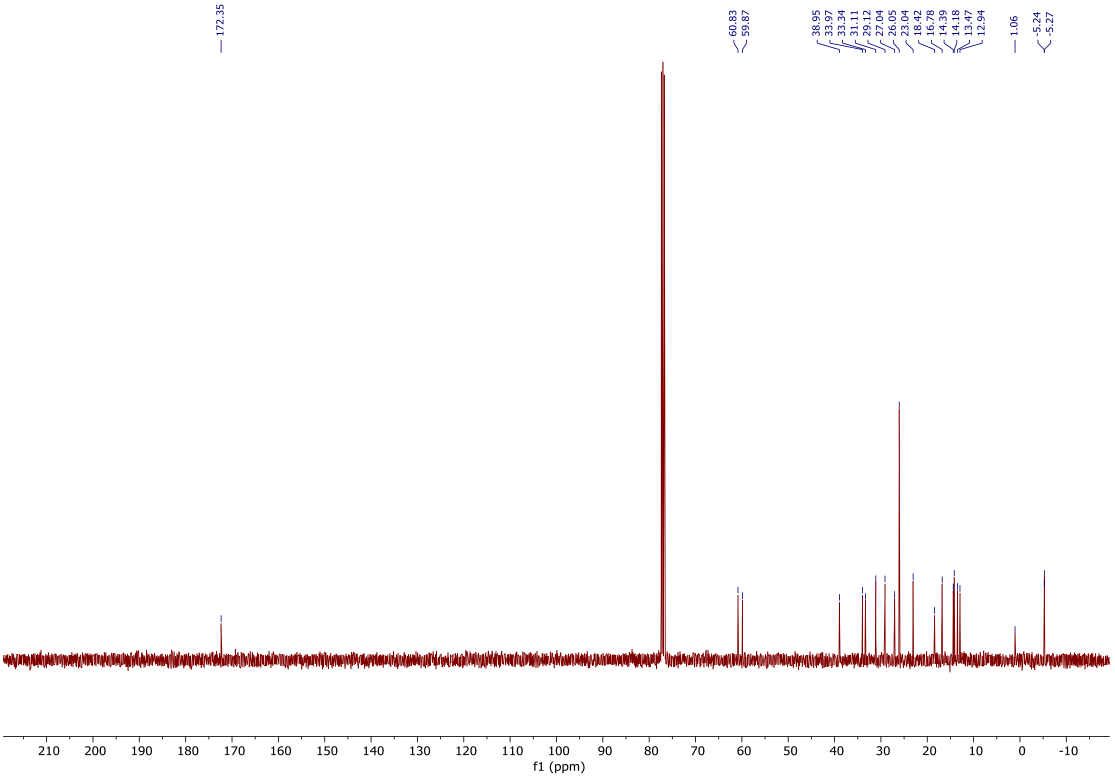

(1S,2S,3R,4S)-ethyl 2-ethyl-4-methyl-2-phenylspiro[2.2]pentane-1-carboxylate (3a-7)

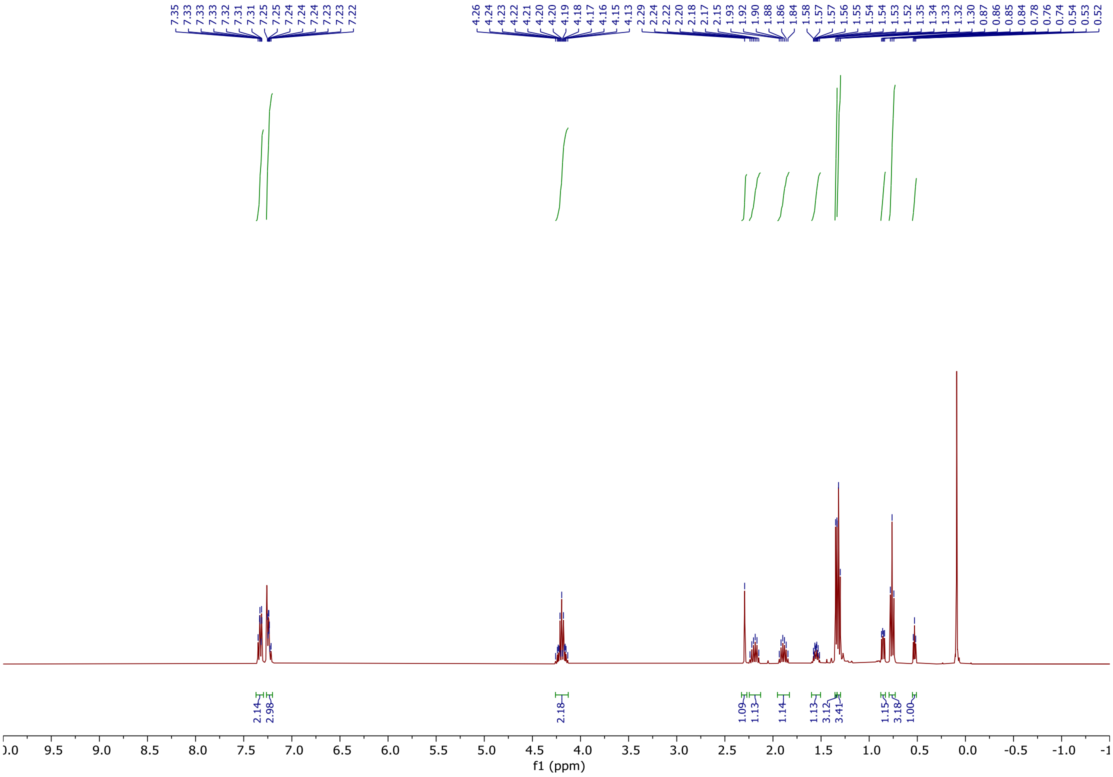

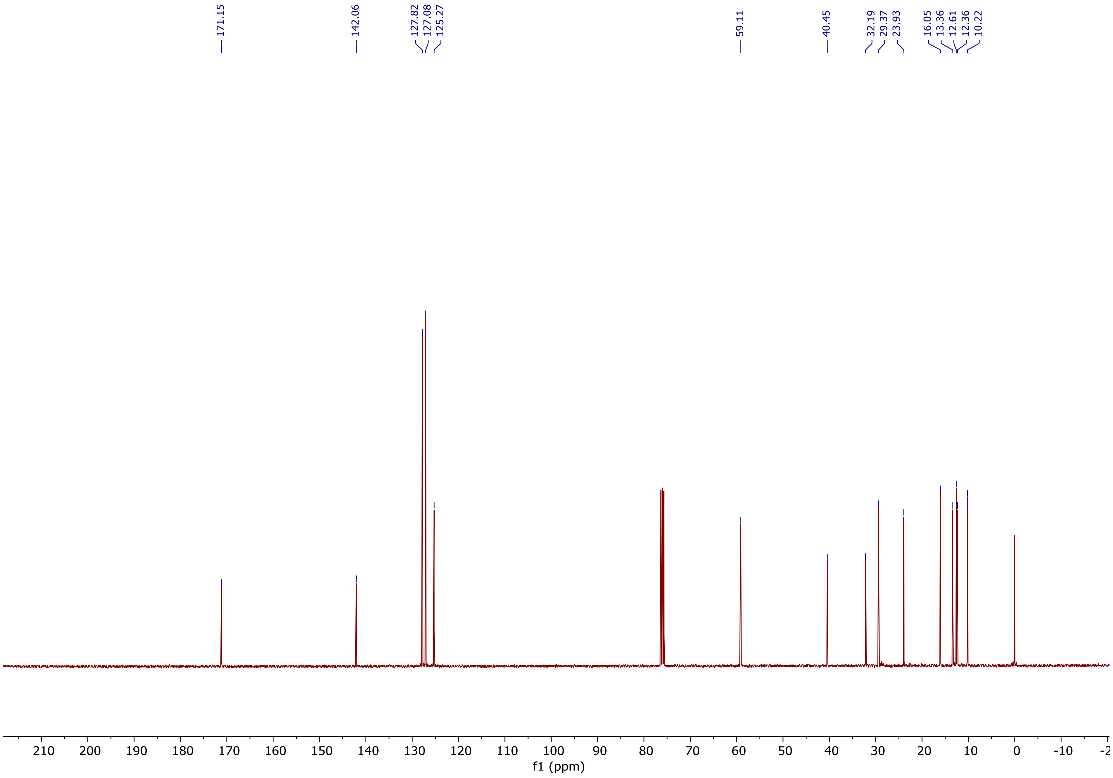

(1R,2R,3R,4S)-ethyl 2-butyl-2,4-diethylspiro[2.2]pentane-1-carboxylate (3a-8)

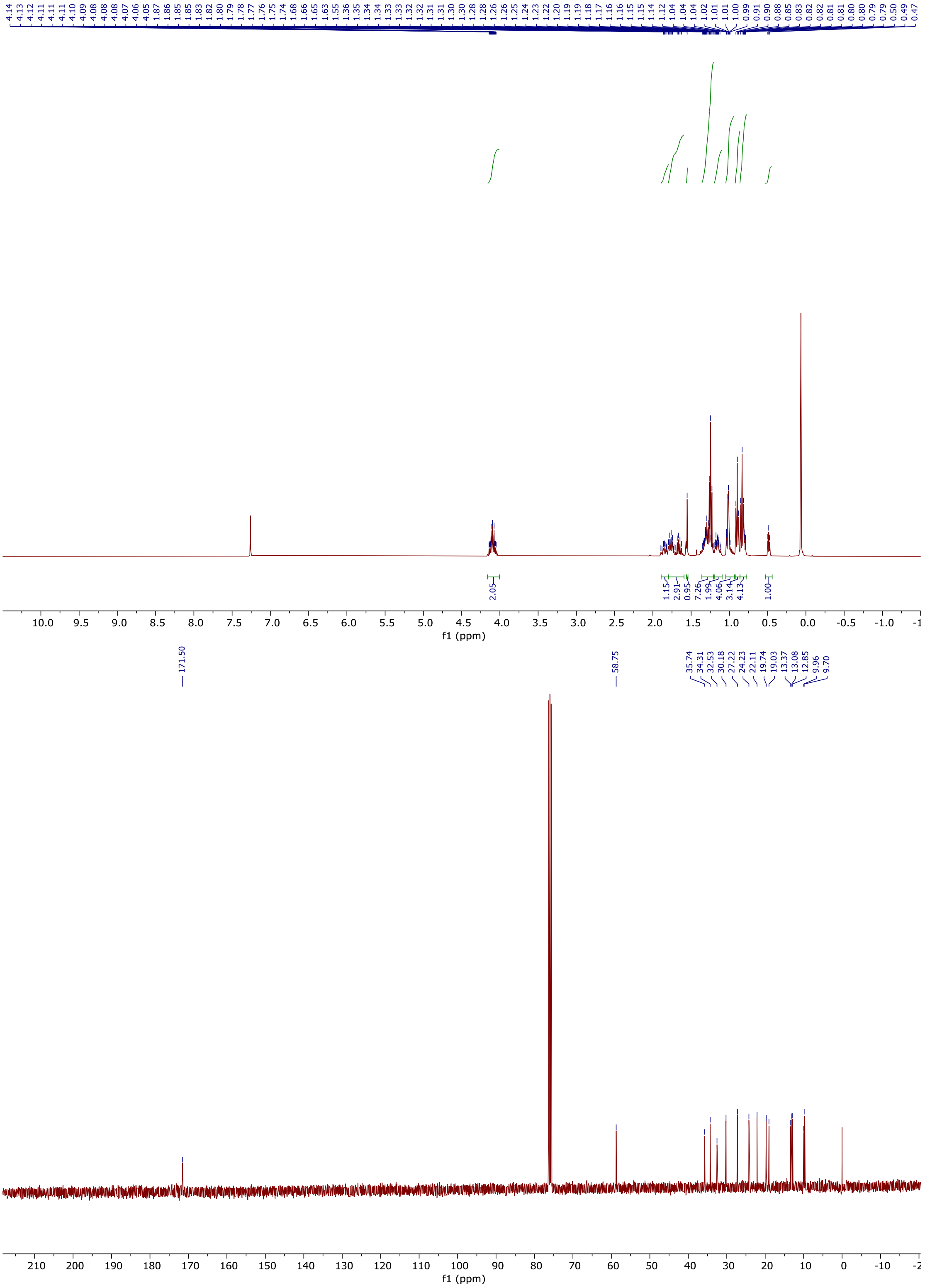

(1R,2R,3R,4S)-ethyl 2-butyl-4-(methoxymethyl)-2-methylspiro[2.2]pentane-1-carboxylate (3a-9)

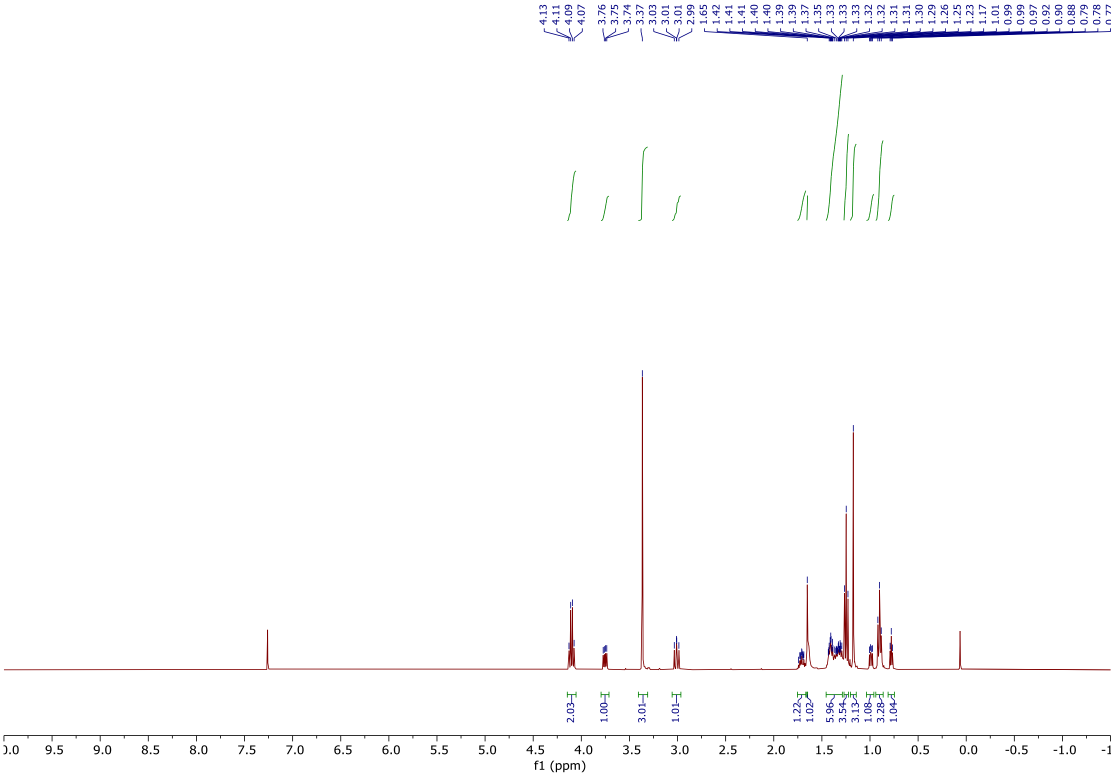

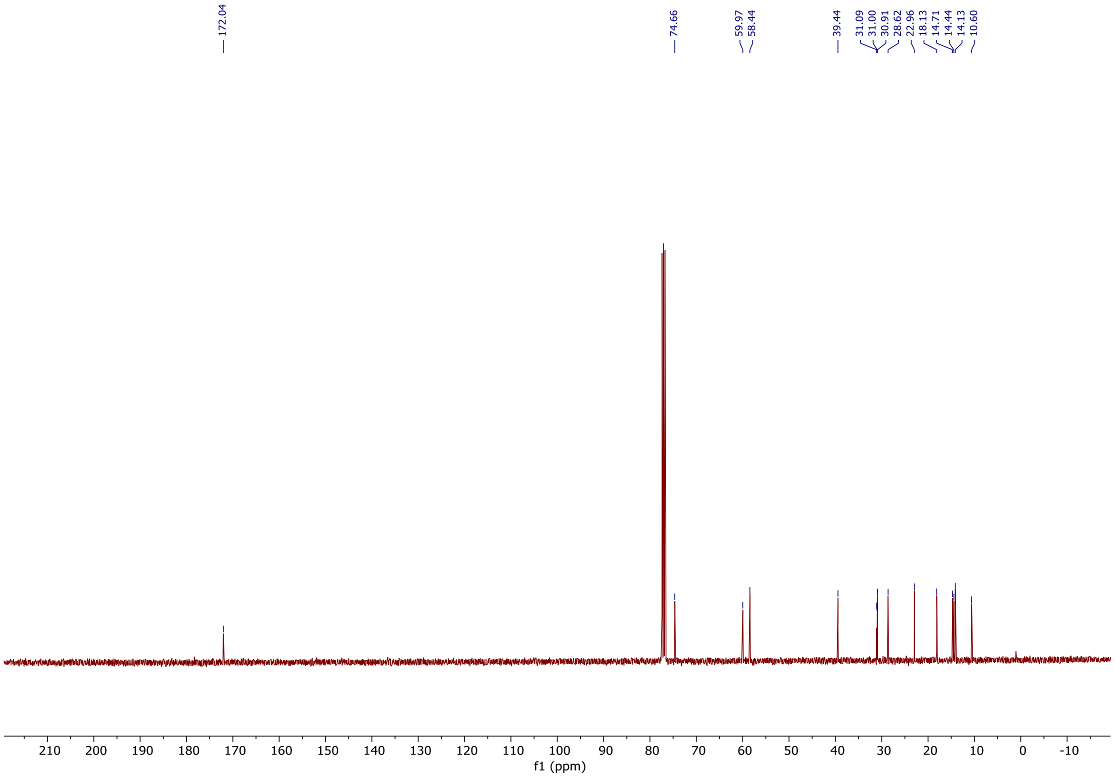

(1R,2R,3s,4R,5S)-ethyl 2-butyl-2-ethyl-4,5-dimethylspiro[2.2]pentane-1-carboxylate (3a-10)

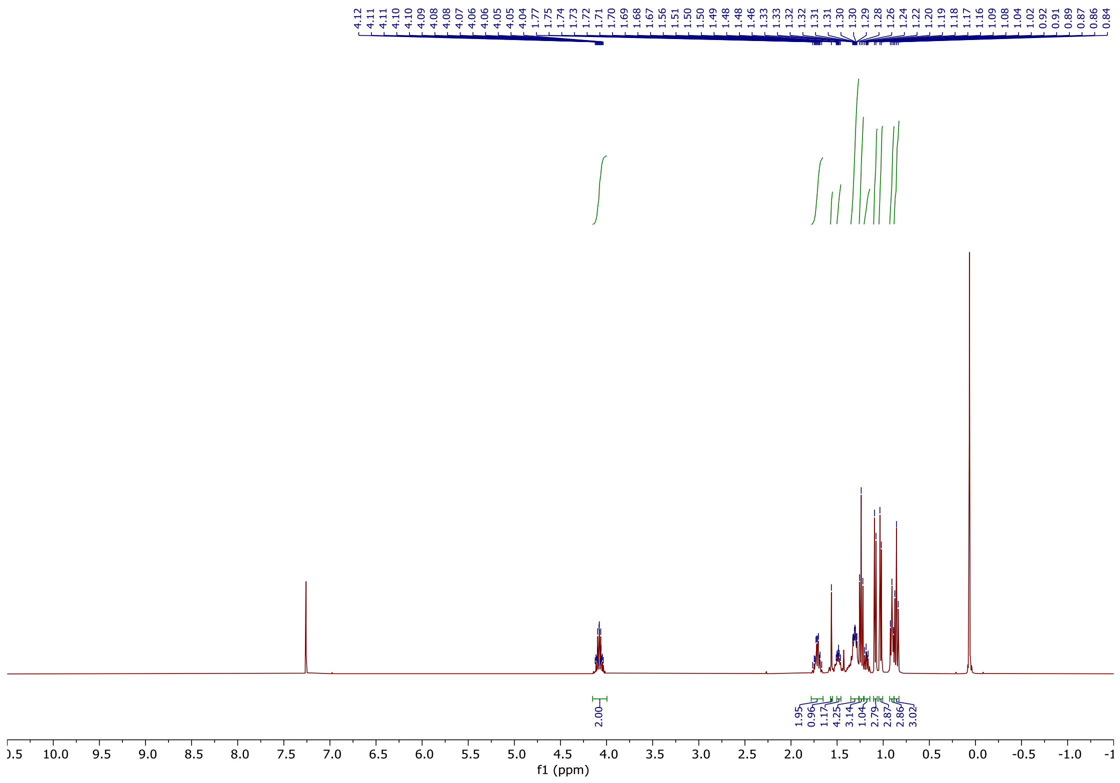

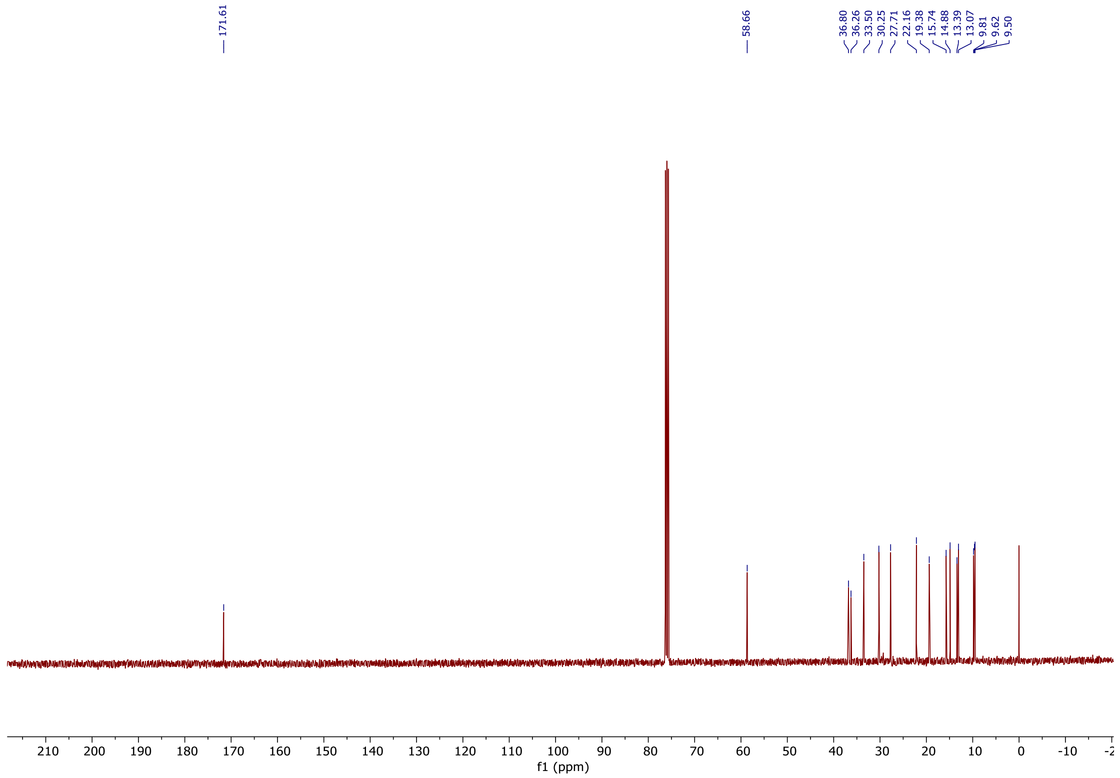

(1R,2S,3S,4S)-ethyl 2-butyl-2-ethyl-1,4-dimethylspiro[2.2]pentane-1-carboxylate (3a-11)

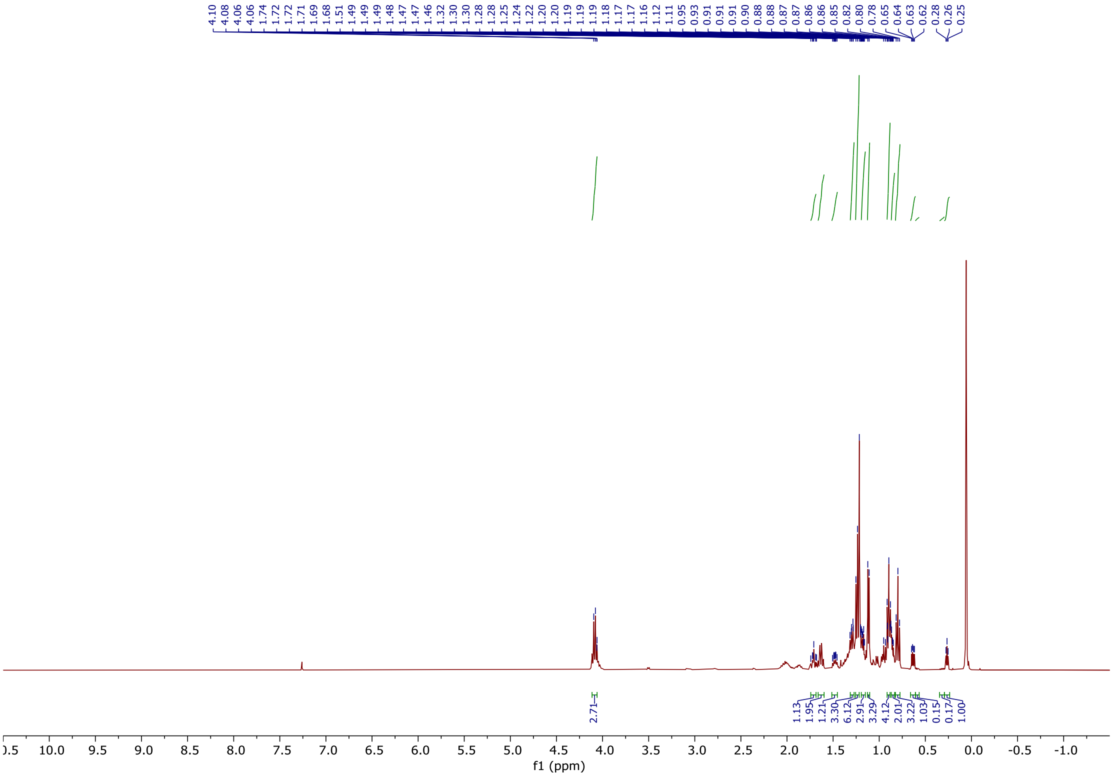

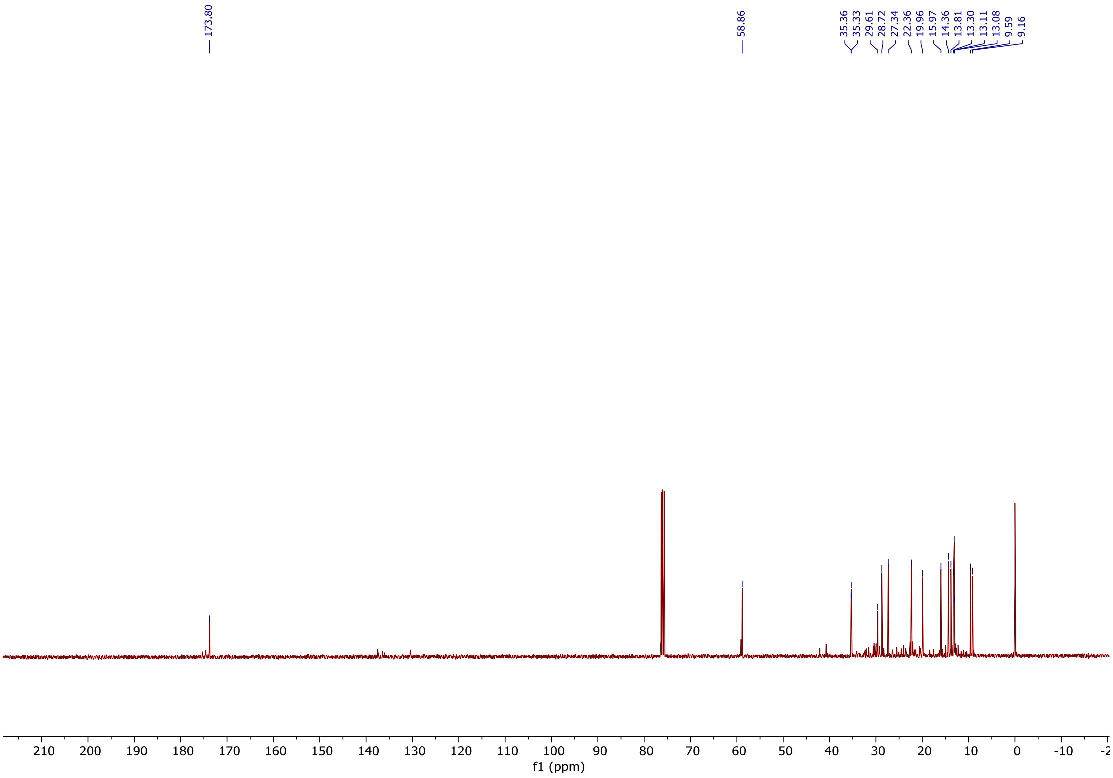

(1R,2S,3S,4R)-ethyl 2-butyl-2-ethyl-1,4-dimethylspiro[2.2]pentane-1-carboxylate (3a-12)

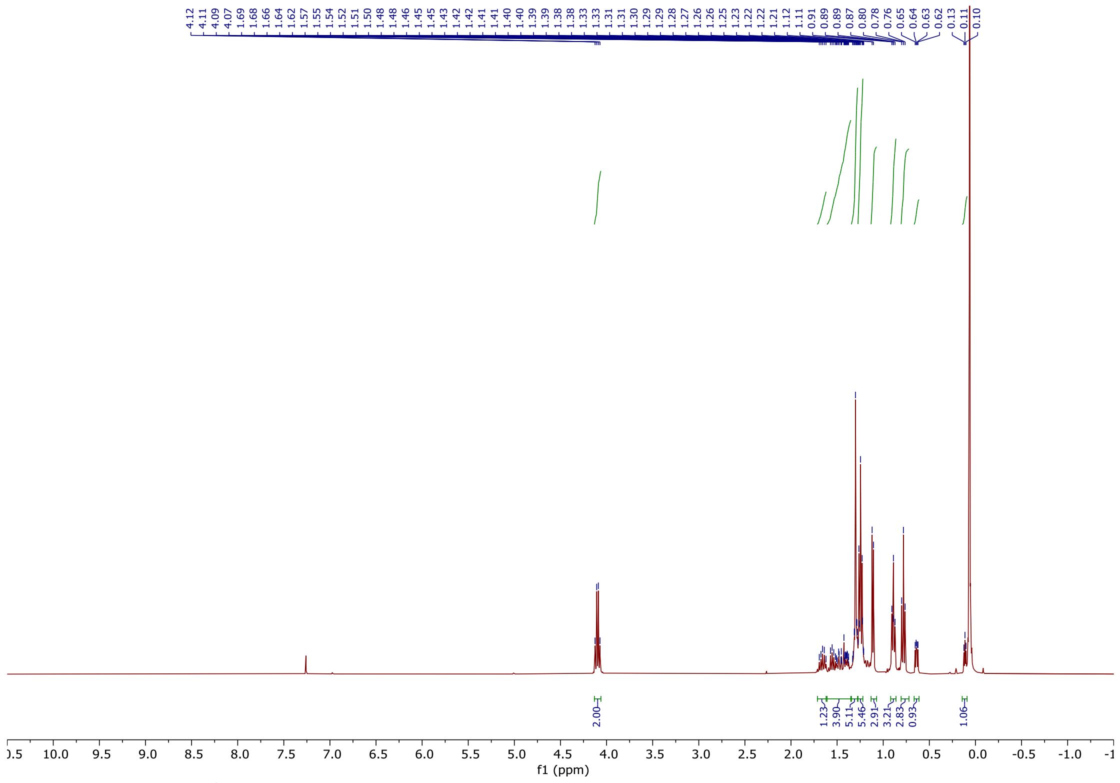

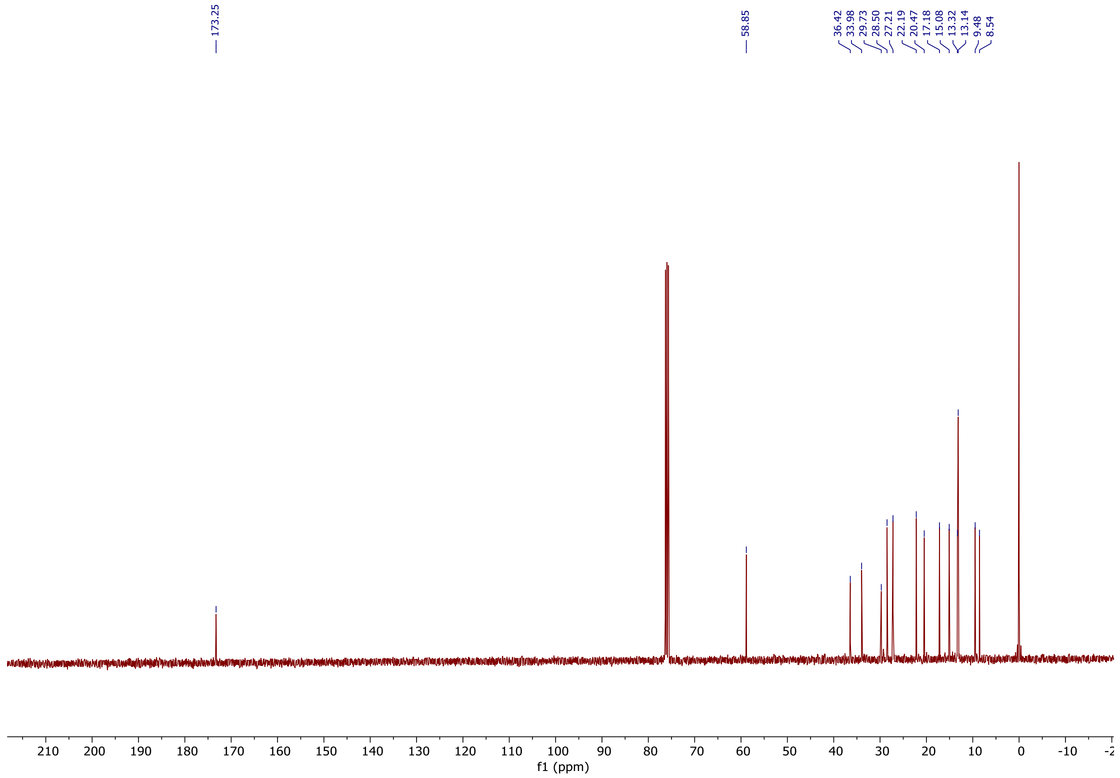

(1S,2S,3S,4S)-ethyl 2-butyl-2-ethyl-4-methylspiro[2.2]pentane-1-carboxylate (3b-1)

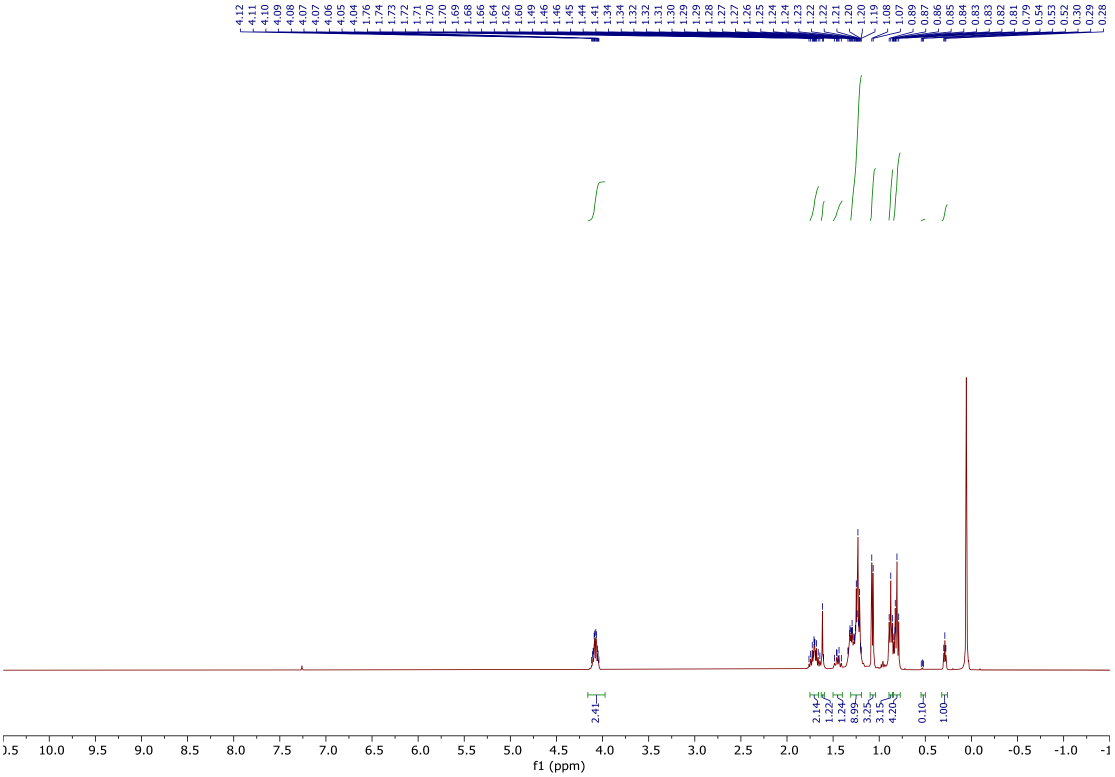

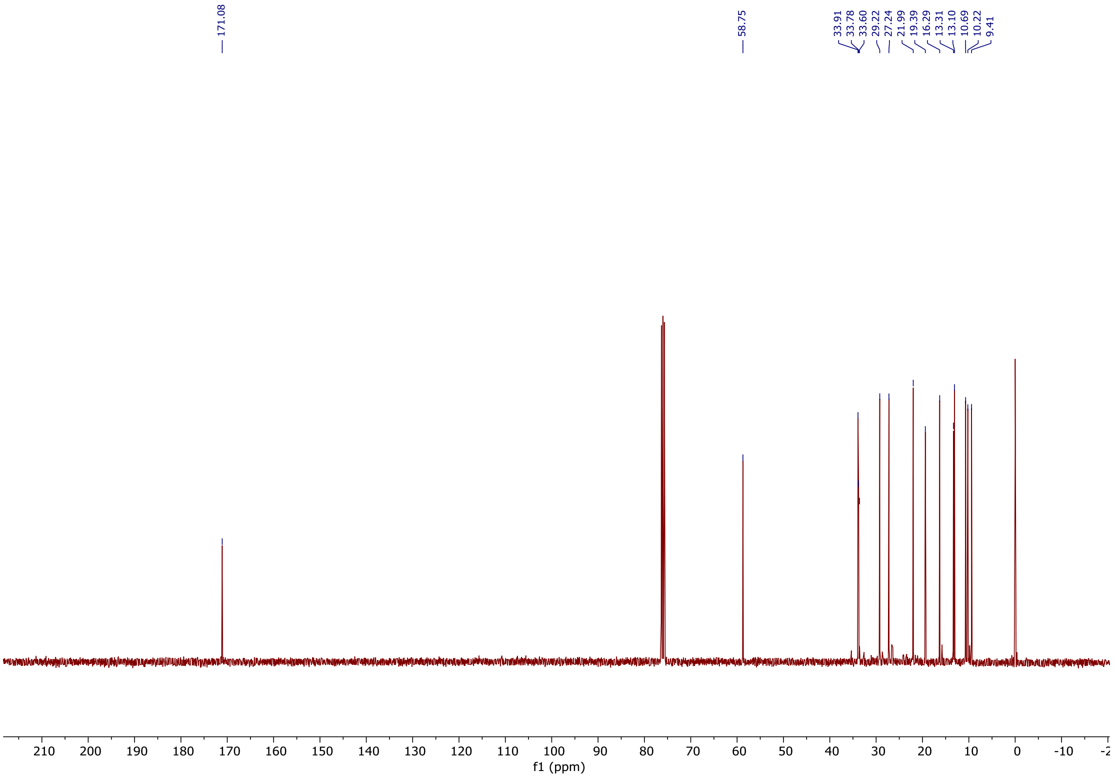

(1S,2S,3S,4S)-ethyl 2-butyl-2-(2-((tert-butyldimethylsilyl)oxy)ethyl)-4-methylspiro [2.2]pentane-1-carboxylate (3b-2)

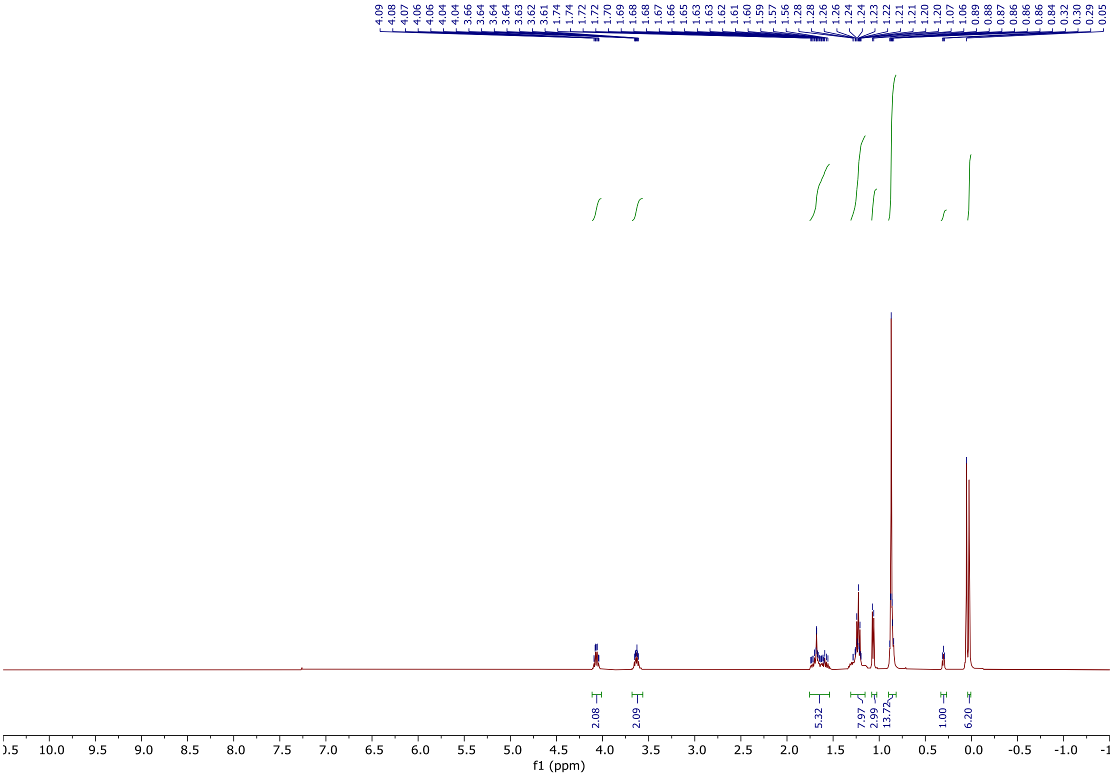

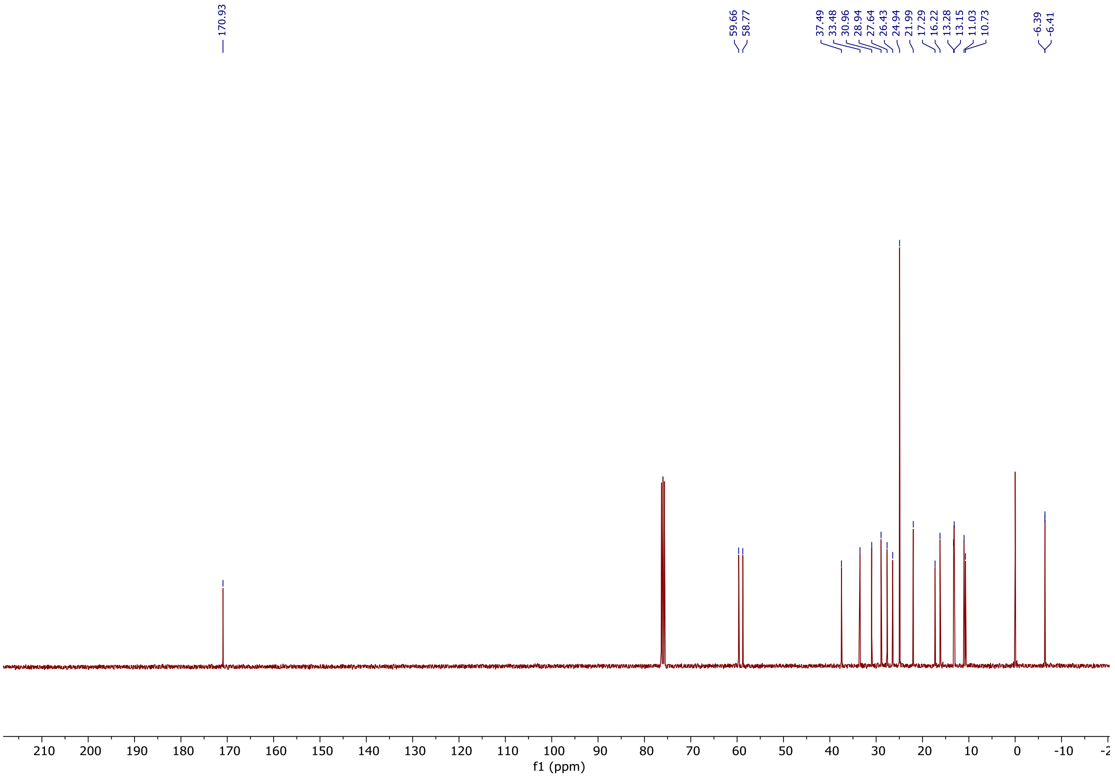

((1S,2S,3S,4S)-2-butyl-2,4-dimethylspiro[2.2]pentan-1-yl)methanol (8b-1)

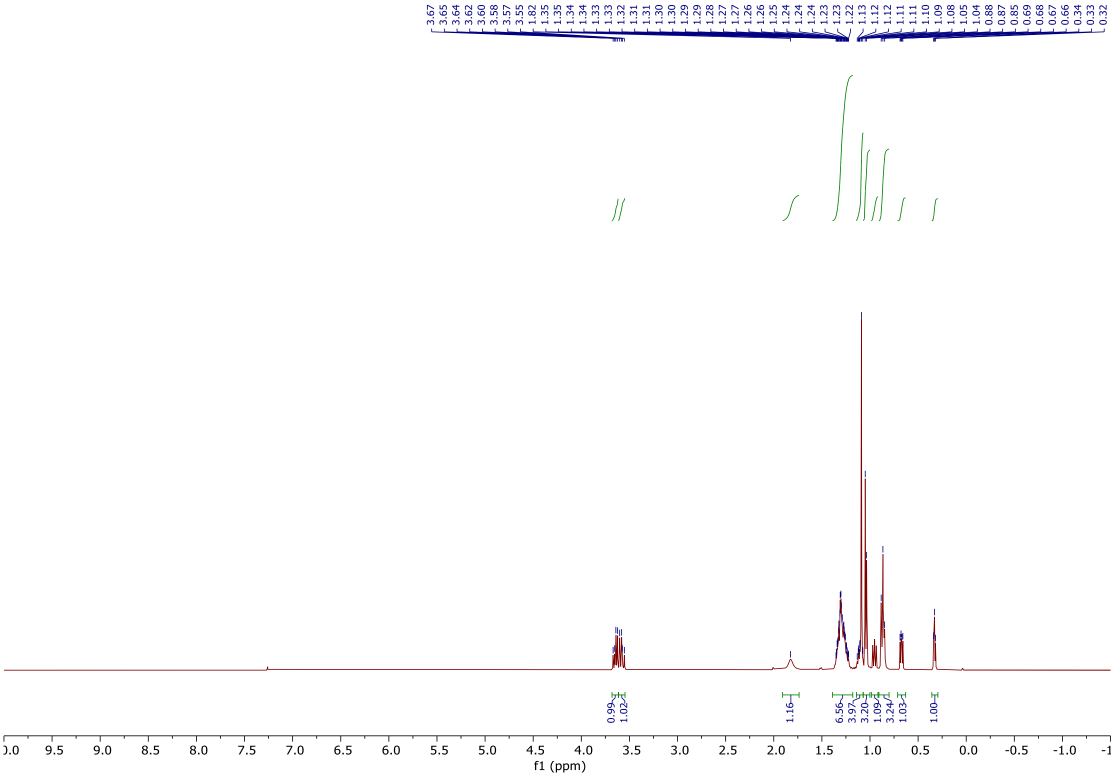

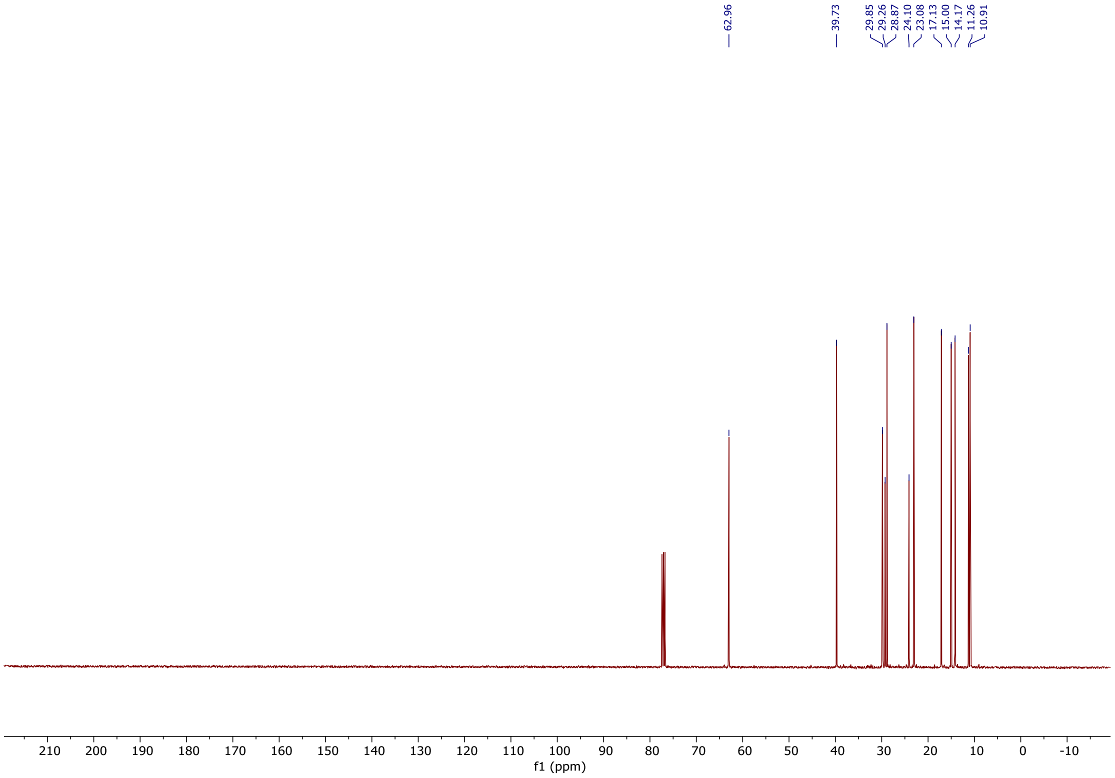

((1R,2R,3R,4S)-2-butyl-2-ethyl-4-methylspiro[2.2]pentan-1-yl)methanol (8a-1)

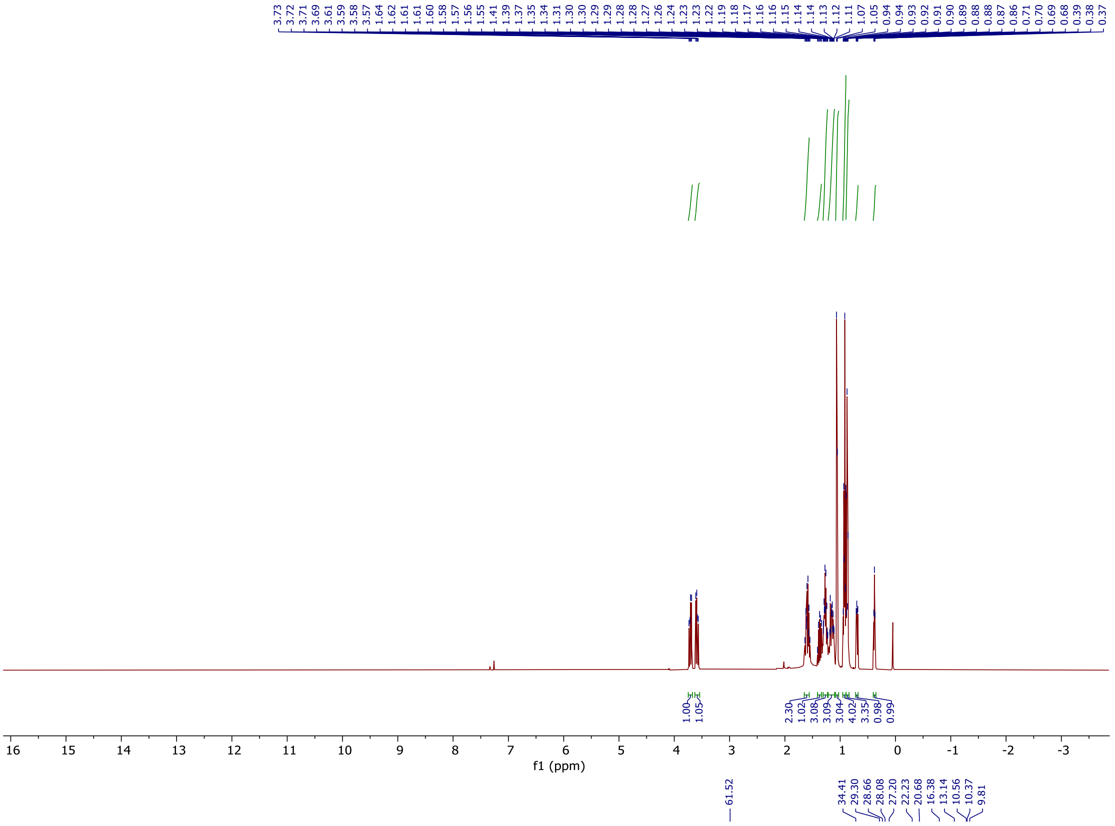

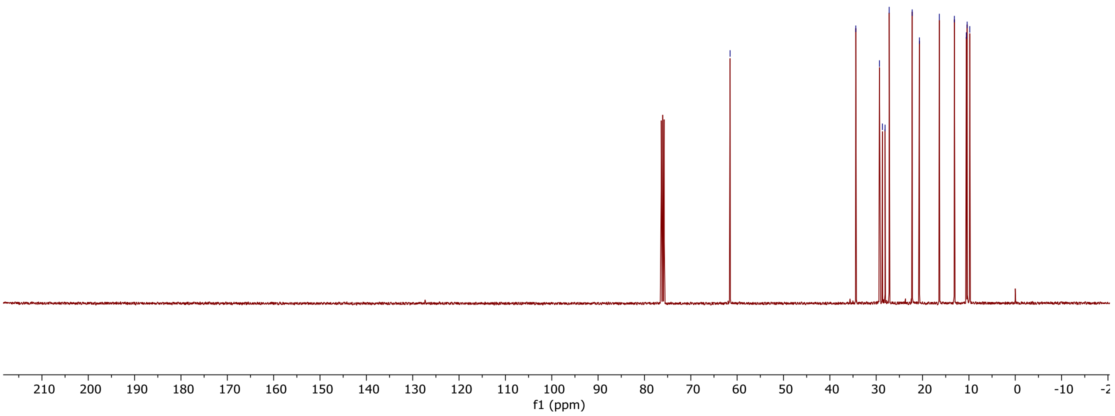

((1R,2S,3R,4S)-2-butyl-2-hexyl-4-methylspiro[2.2]pentan-1-yl)methanol (8a-2)

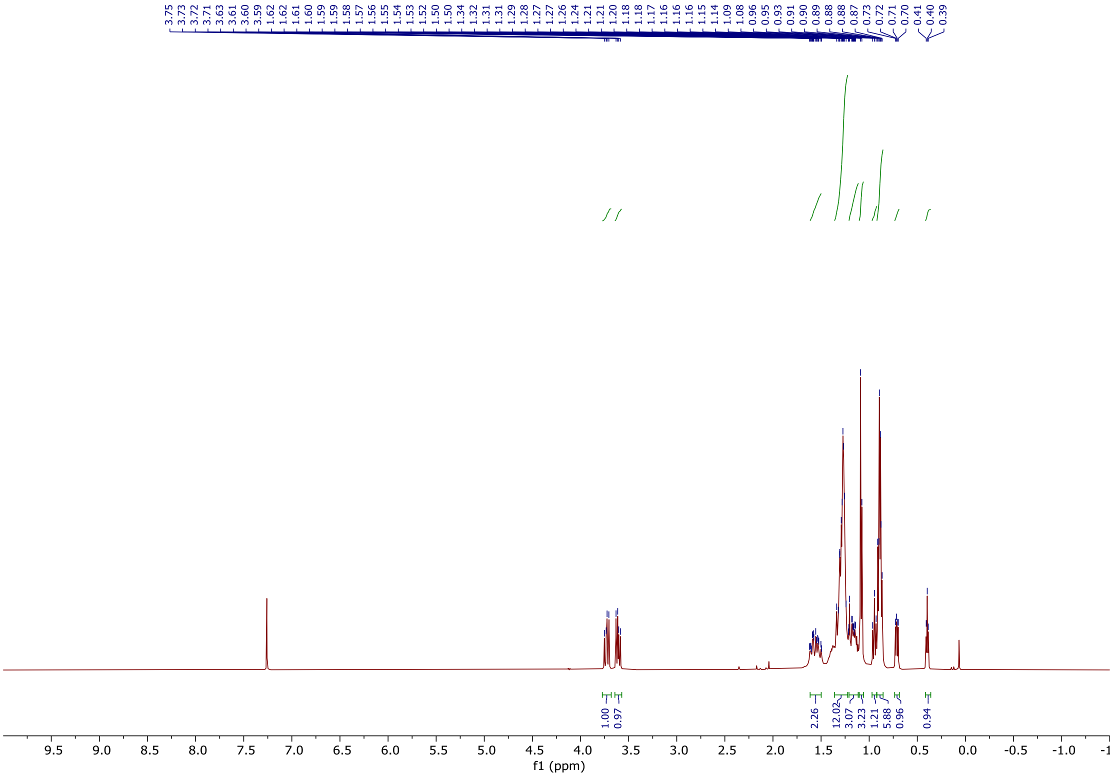

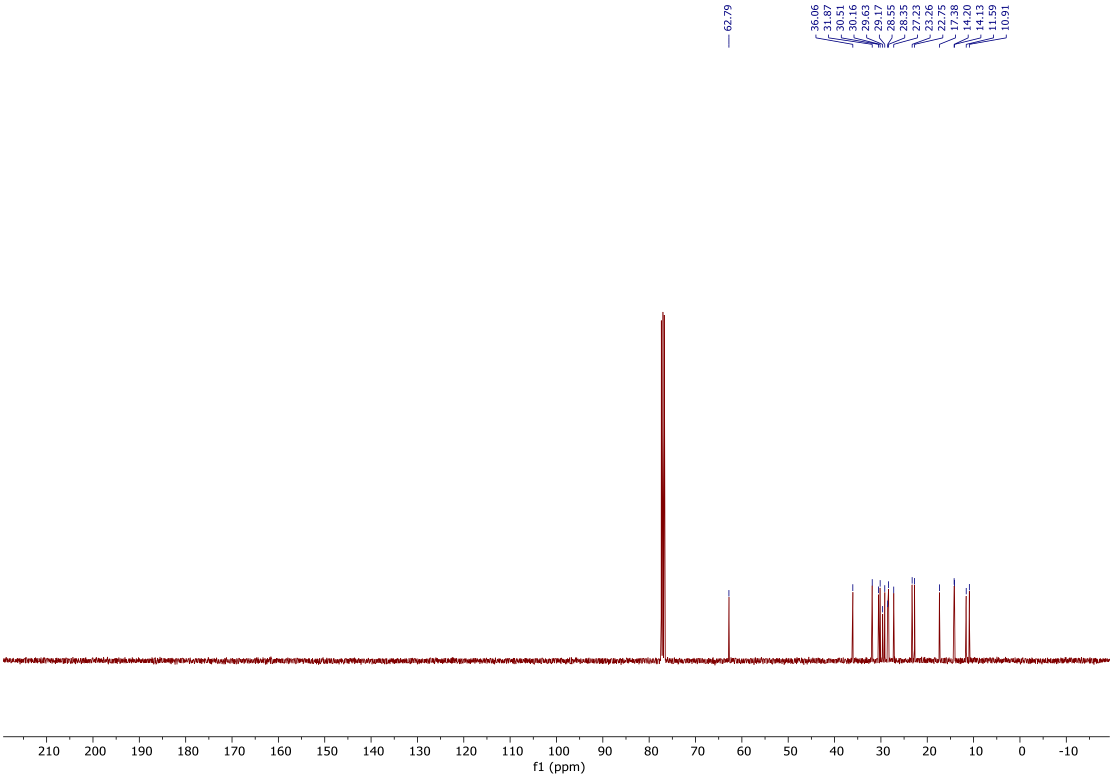

((1R,2R,3R,4S)-2-butyl-2,4-dimethylspiro[2.2]pentan-1-yl)methanol (8a-3)

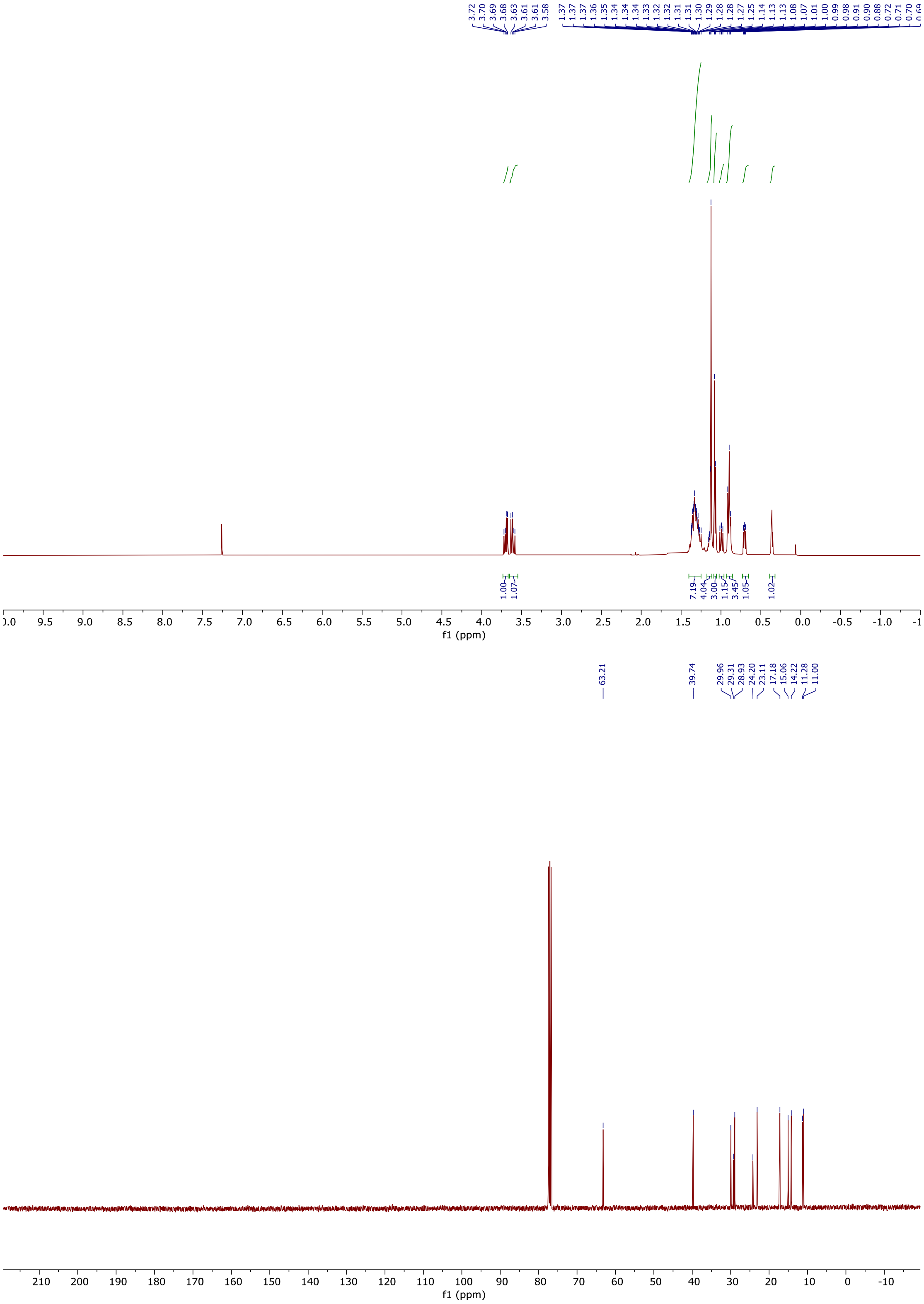

18. NMR spectra of miscellaneous compounds.

Triethyl((5-phenylpent-4-yn-2-yl)oxy)silane (si-1)

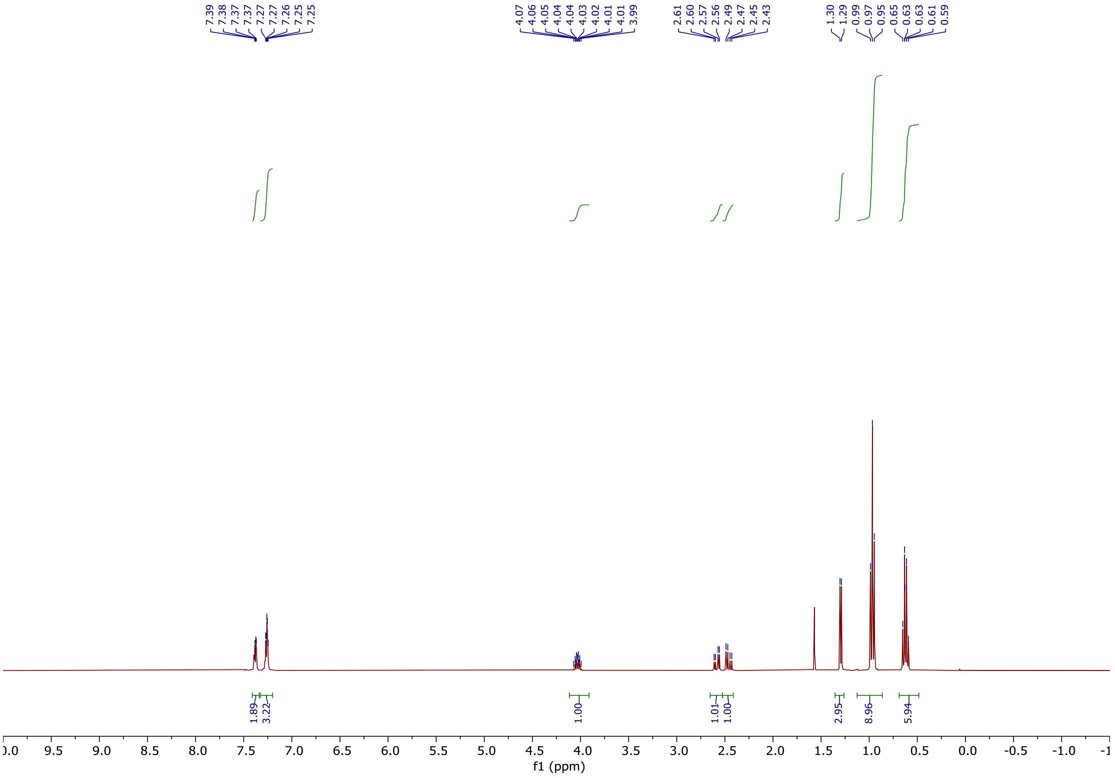

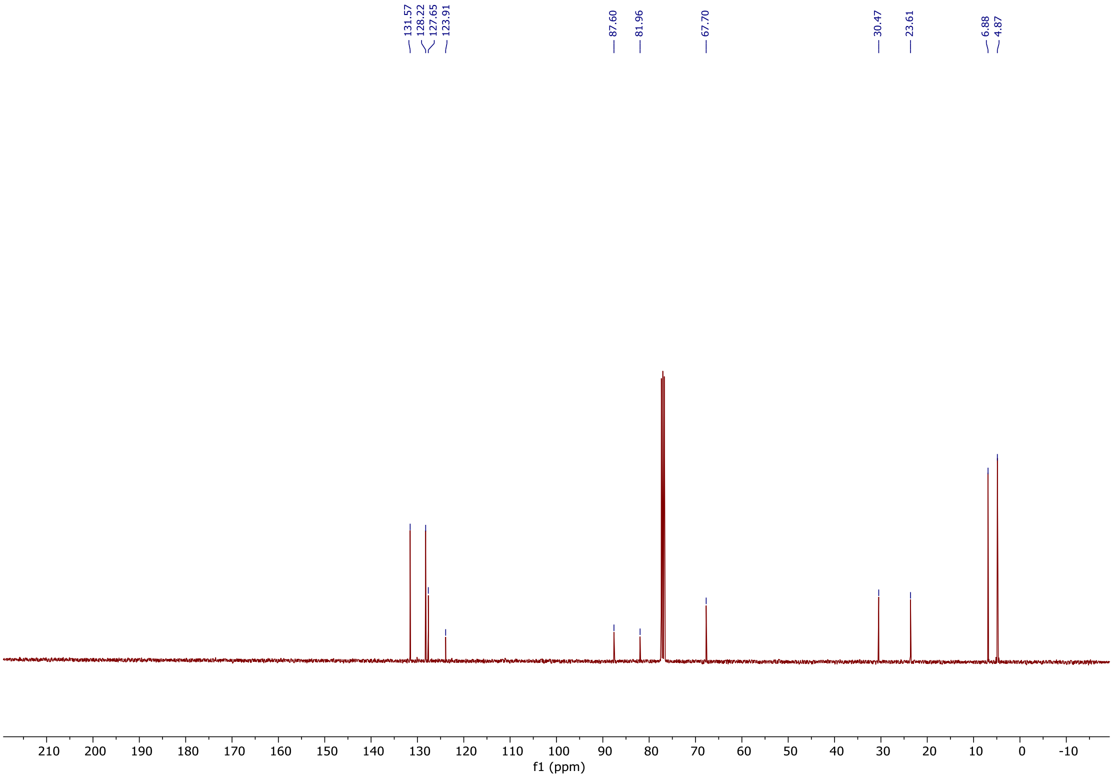

Ethyl 2-phenyl-3-(2-((triethylsilyl)oxy)propyl)cycloprop-2-enecarboxylate (si-2)

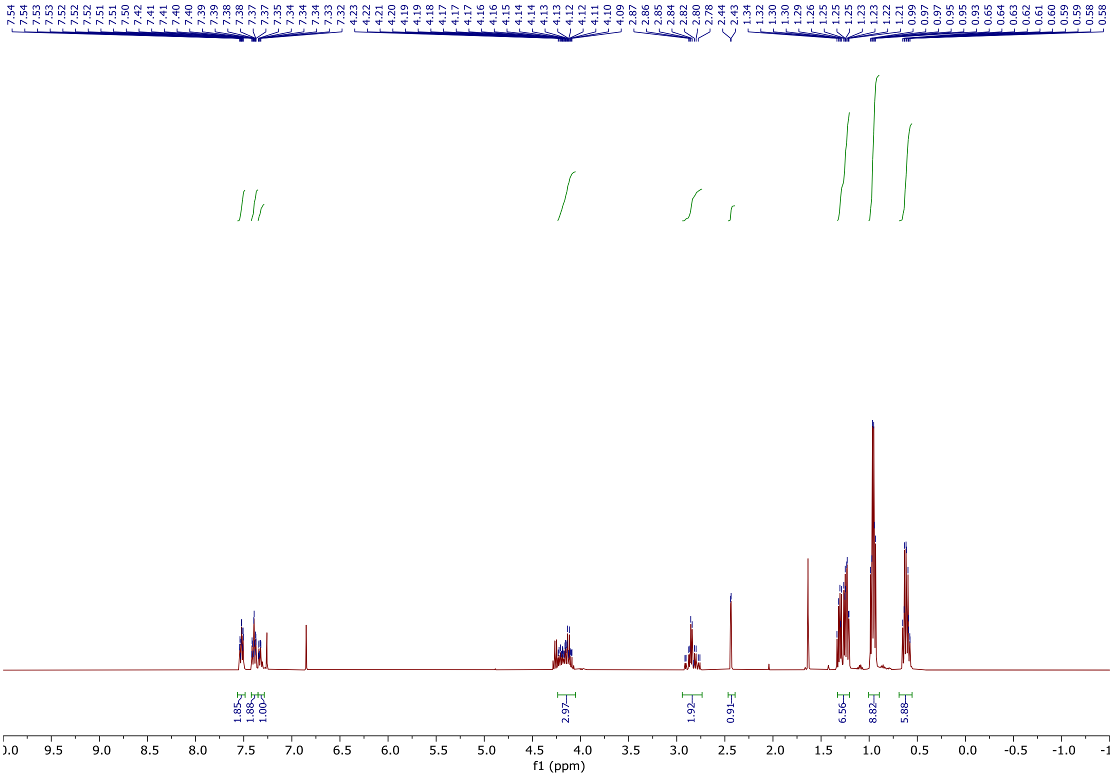

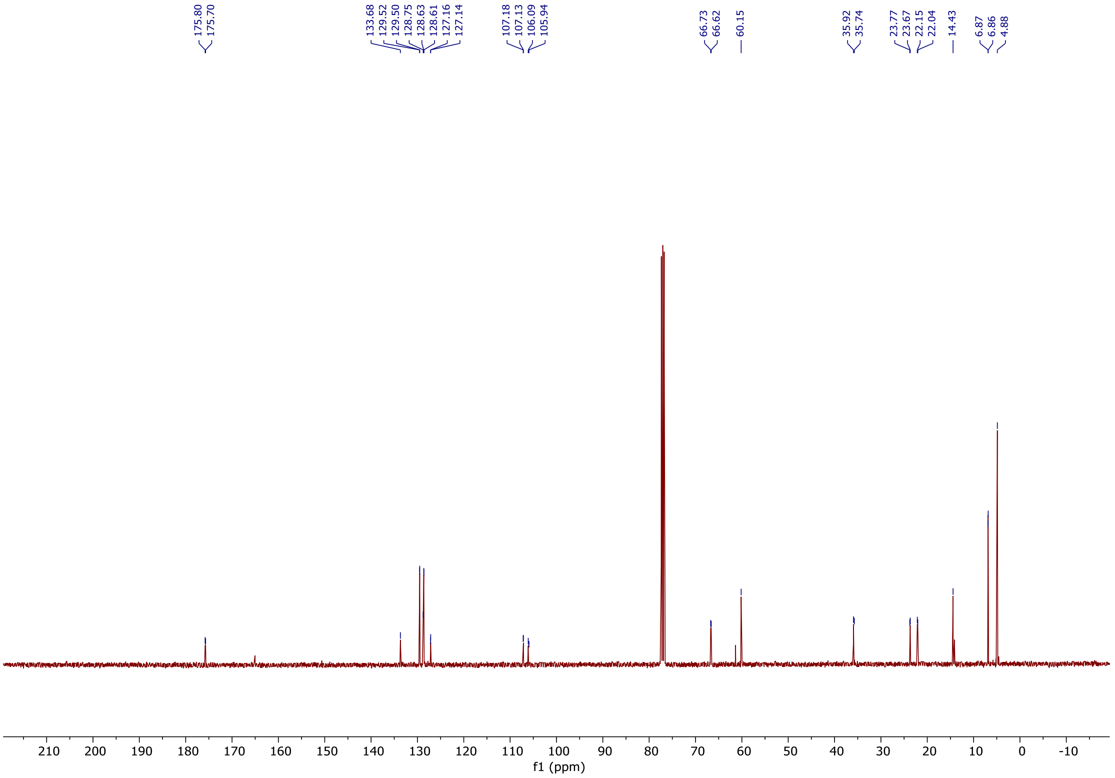

Triethyl(((2R,3S)-3-methylnon-4-yn-2-yl)oxy)silane (si-3)

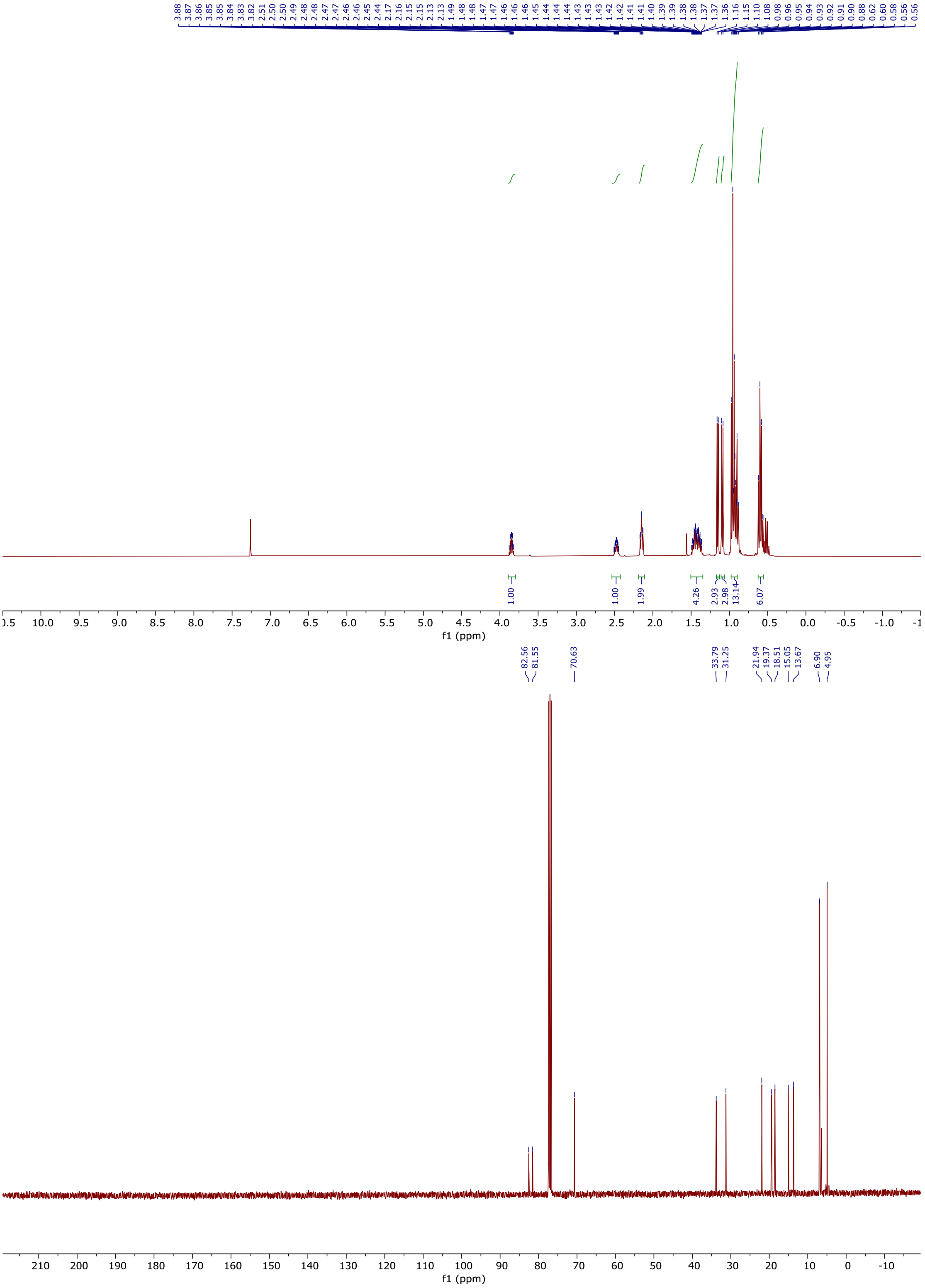

Ethyl 2-butyl-3-((2S,3R)-3-((triethylsilyl)oxy)butan-2-yl)cycloprop-2-enecarboxylate (si-4)

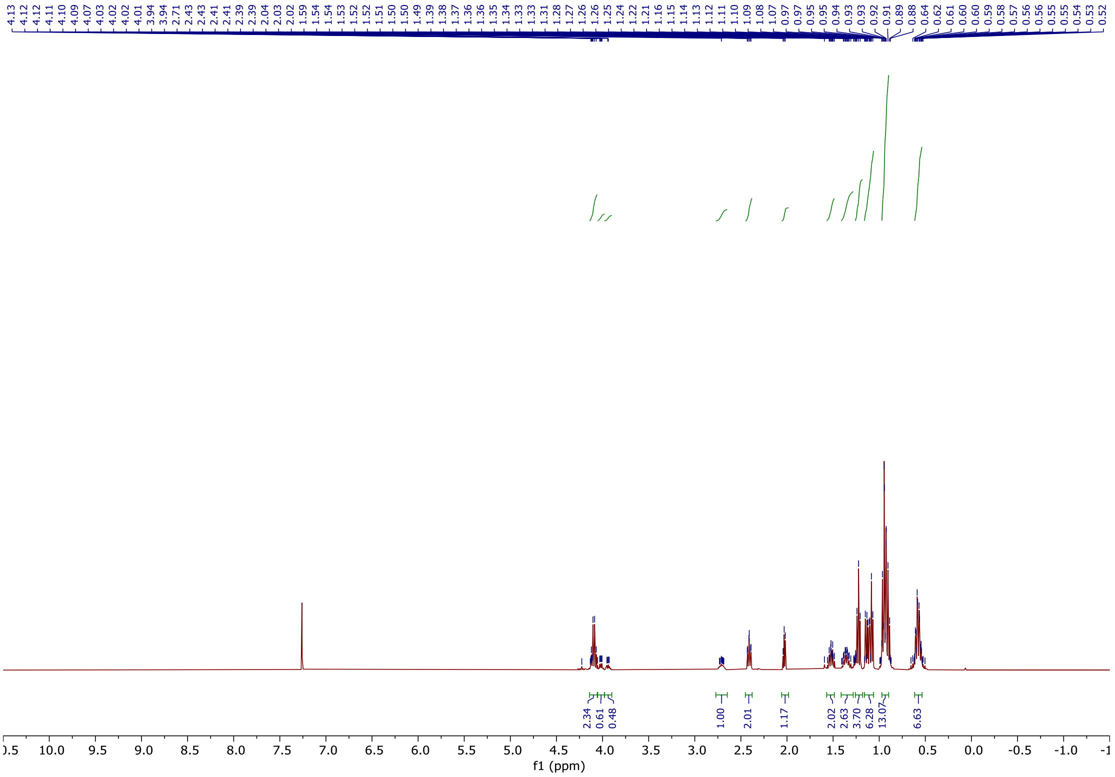

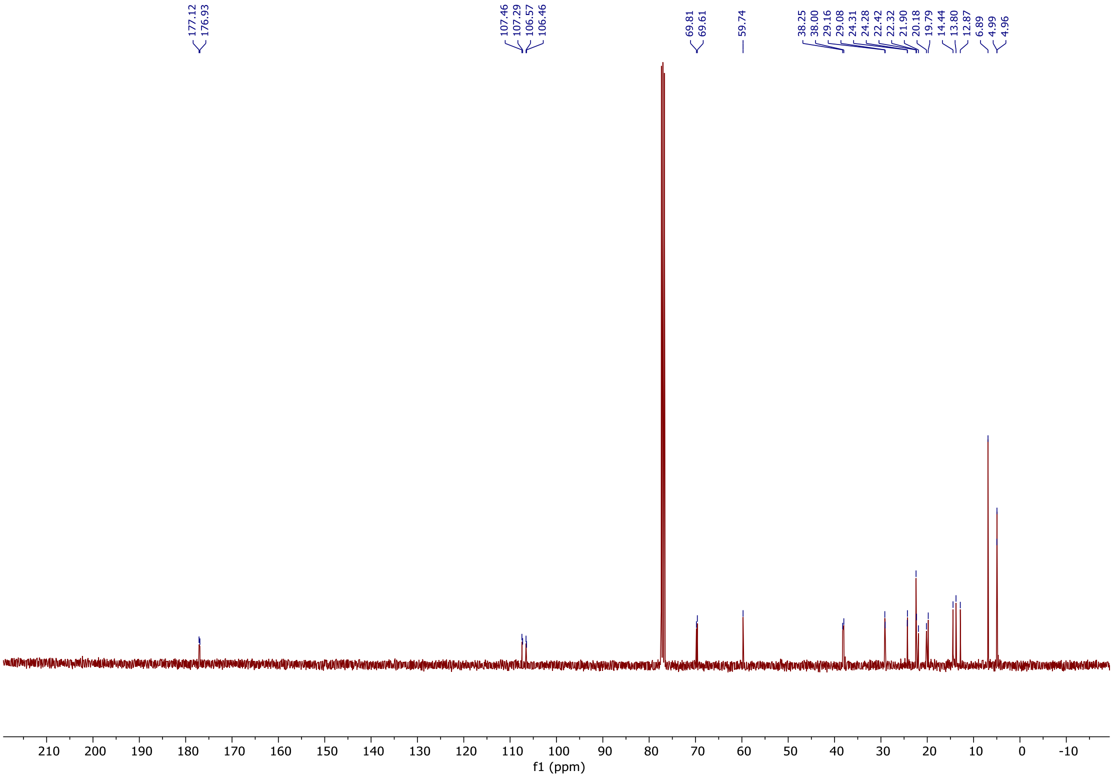

((1S,2S,3R,4S)-2-ethyl-4-methyl-2-phenylspiro[2.2]pentan-1-yl)methanol (si-5)

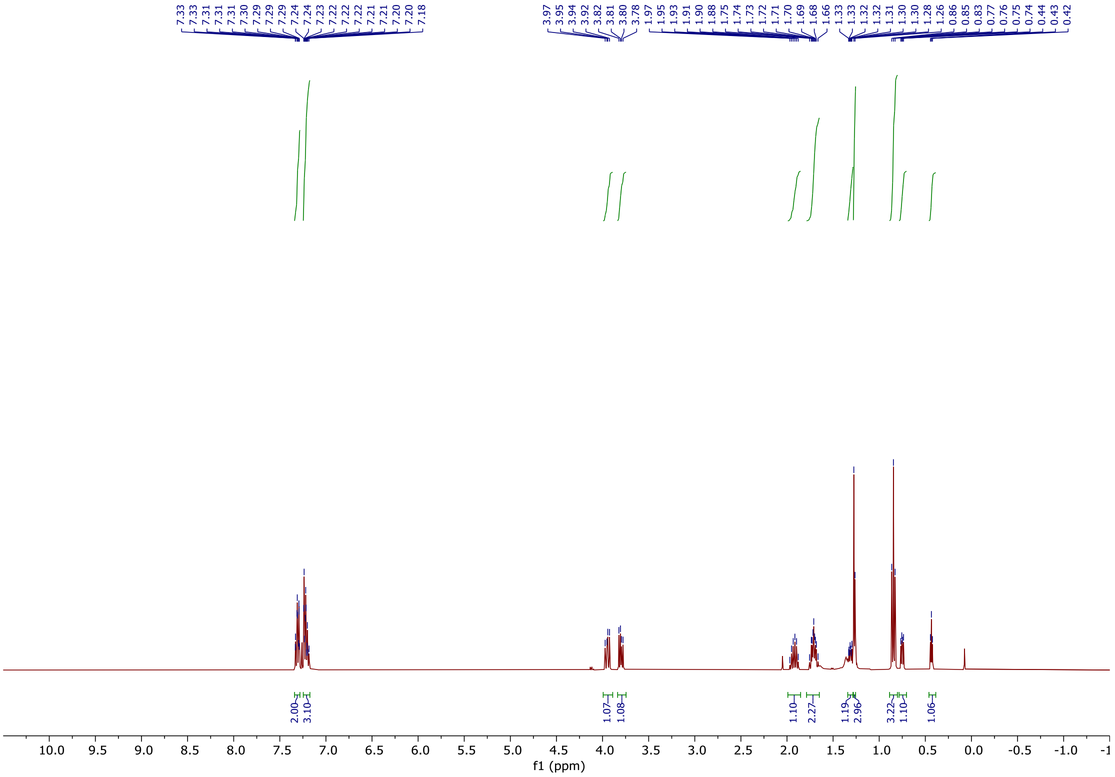

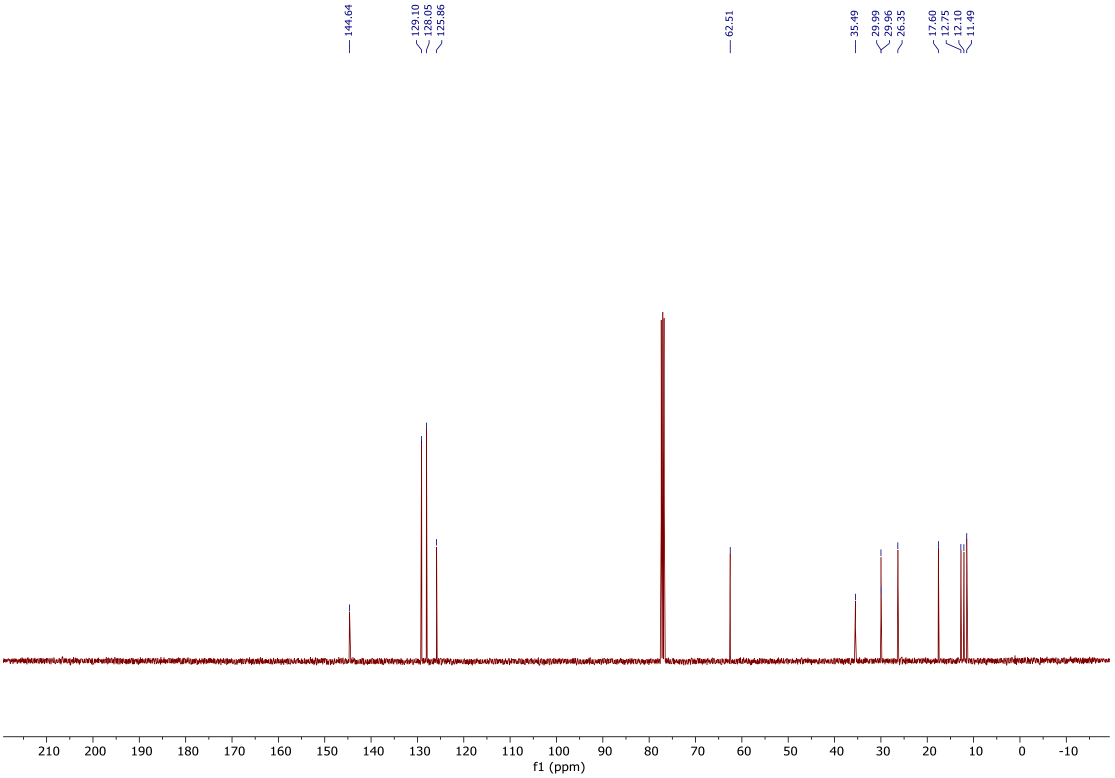

(1S,2S,3R,4S)-2-ethyl-4-methyl-2-phenylspiro[2.2]pentane-1-carbaldehyde (si-6)

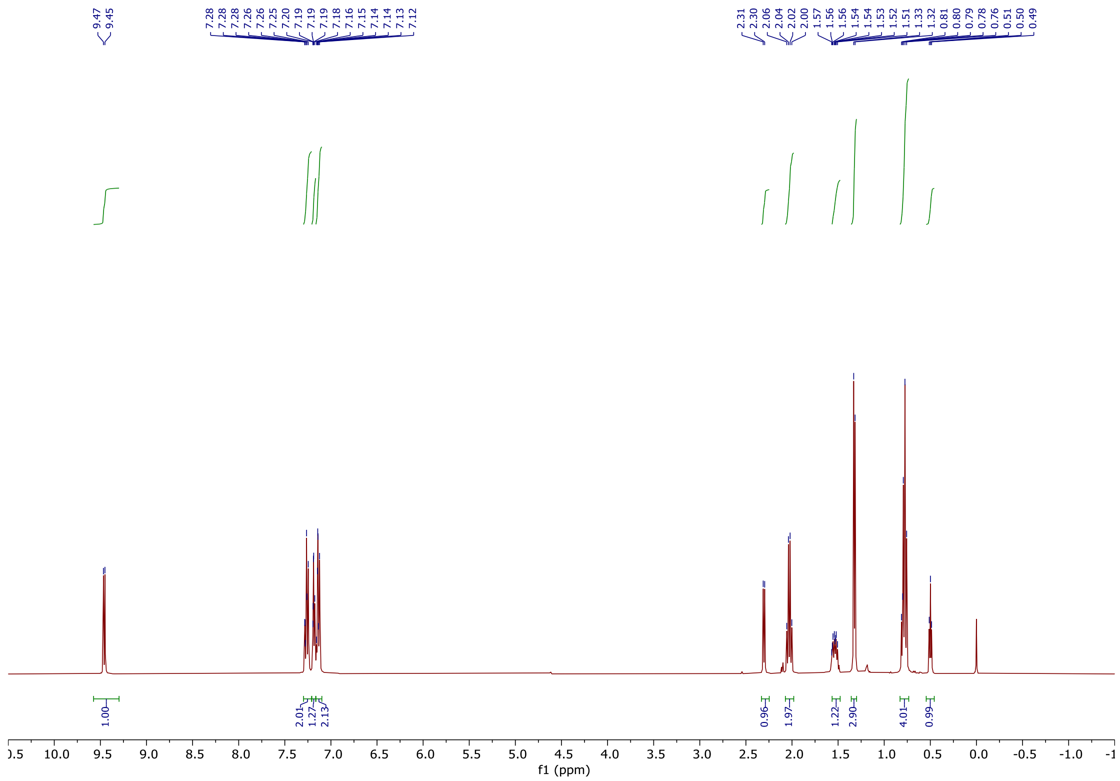

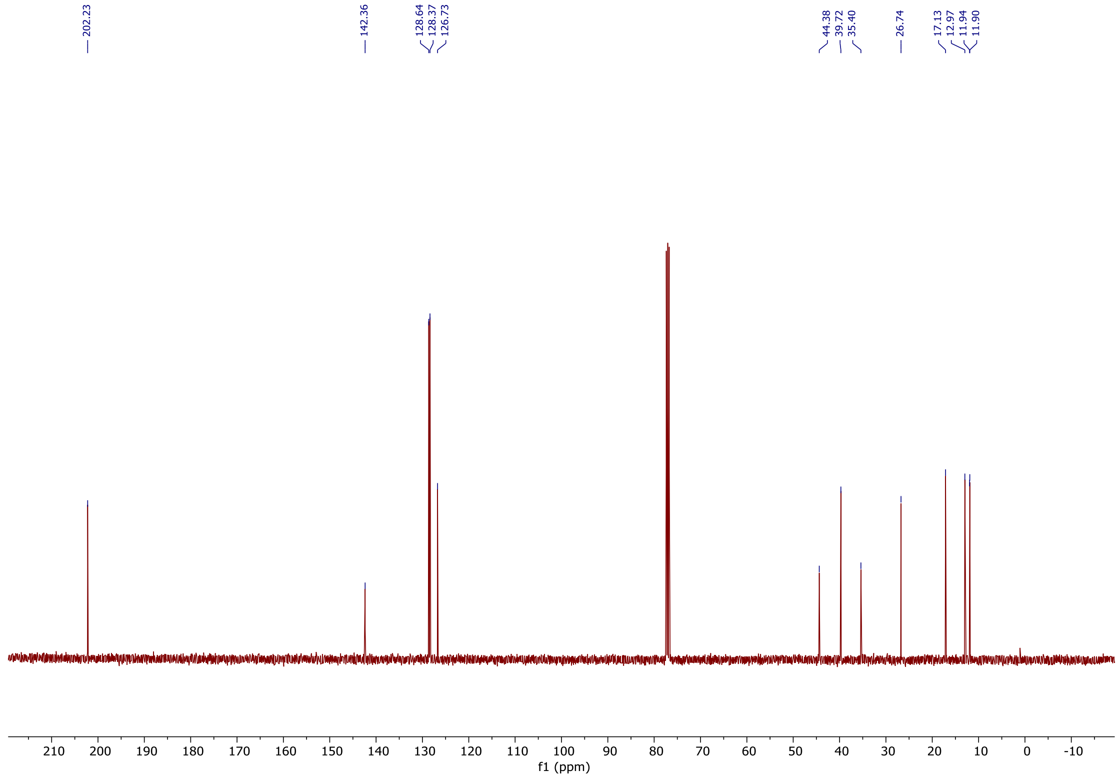

(2,4-dinitrophenyl)-2-(((1S,2S,3R,4S)-2-ethyl-4-methyl-2-phenylspiro[2.2]pentan-1-yl)methylene)hydrazine (si-7)

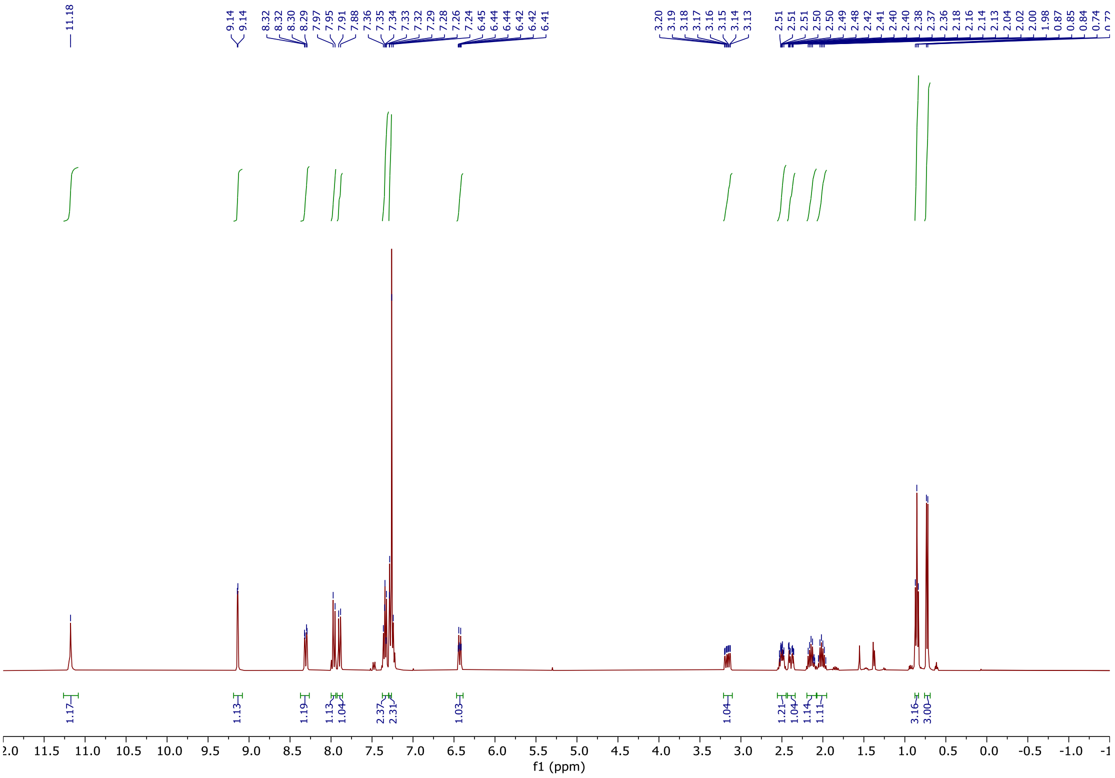

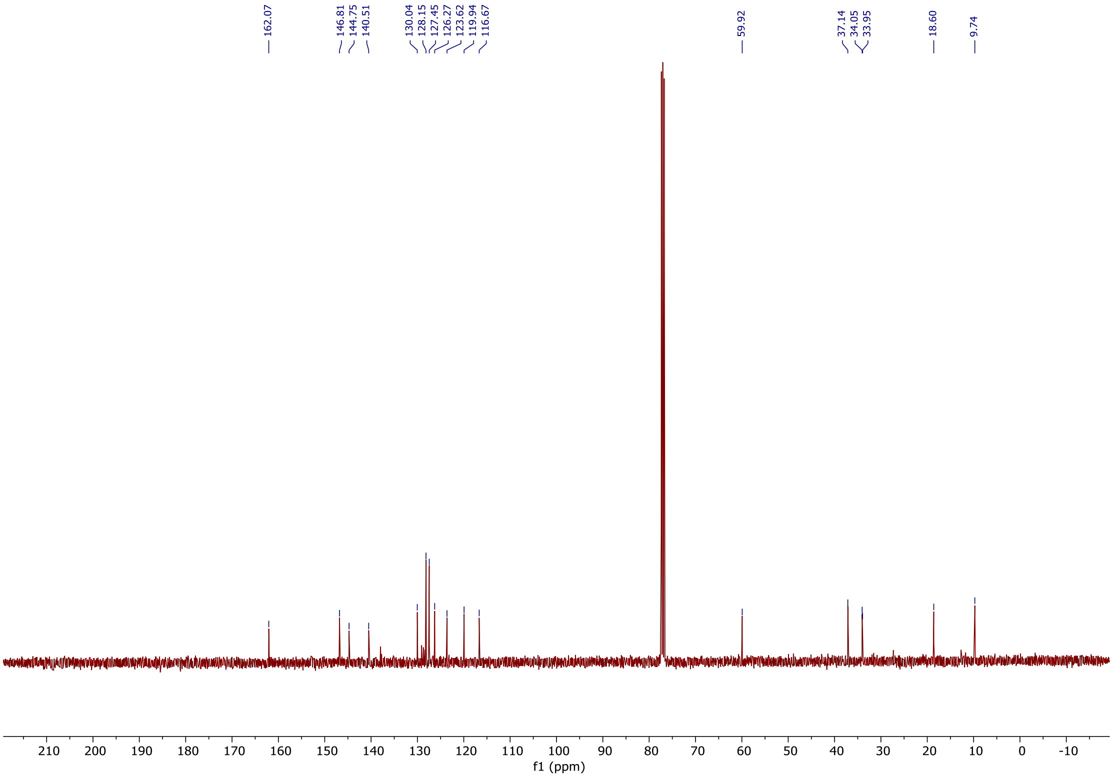

Supplement: Supplementary file 1 — ja2c07370_si_001.pdf [file ja2c07370_si_001.pdf]
